# Focus on single-gene effects limits discovery and interpretation of complex-trait-associated variants

**DOI:** 10.1016/j.ajhg.2026.02.022

**Published:** 2026-03-23

**Authors:** Kathryn A. Lawrence, Tamara Gjorgjieva, Daniel Nachun, Stephen B. Montgomery

**Affiliations:** 1 Department of Genetics, Stanford University School of Medicine, Stanford, CA 94305, USA; 2 Department of Pathology, Stanford University School of Medicine, Stanford, CA 94305, USA; 3 Department of Biomedical Data Science, Stanford University School of Medicine, Stanford, CA 94305, USA

**Keywords:** cis-eQTL, multi-gene analysis, gene clusters, co-expression, principal-component QTL, allelic pleiotropy, allelic proxitropy, GTEx, GWAS colocalization, complex-trait-associated variation

## Abstract

Standard quantitative trait locus (QTL) mapping approaches consider variant effects on a single gene at a time, despite abundant evidence of allelic pleiotropy, where a single variant can affect multiple genes simultaneously. While allelic pleiotropy describes variant effects on both local and distal genes or a mixture of molecular effects on a single gene, here, we specifically investigate allelic expression “proxitropy,” where a single variant influences the expression of multiple, neighboring genes. We introduce a multi-gene expression QTL (eQTL) mapping framework—*cis*-principal-component eQTL (*cis*-pc eQTL or pcQTL)—to identify variants associated with shared axes of expression variation across a cluster of neighboring genes. We perform pcQTL mapping in 13 GTEx human tissues and discover novel loci undetected by single-gene approaches. In total, we identify an average of 1,396 pcQTLs/tissue, 27% of which were not discovered by single-gene methods. These novel pcQTLs colocalized with an additional 176 genome-wide association study (GWAS) trait-associated variants and increased the number of colocalizations by 33% over single-gene QTL mapping. These findings highlight the idea that moving beyond single-gene-at-a-time approaches toward multi-gene methods can offer a more comprehensive view of gene regulation and complex-trait-associated variation.

## Graphical abstract

**Figure:**
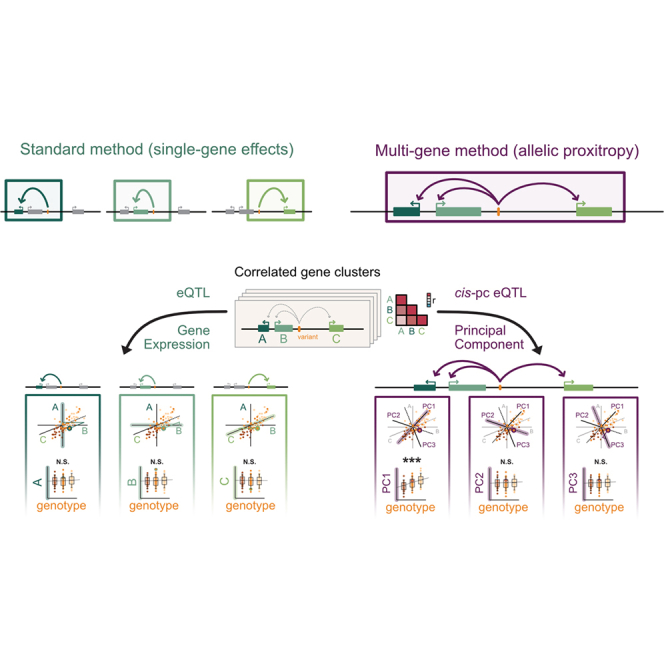

## Teaser

Standard eQTL mapping and GWAS interpretation assume that each variant acts on one gene. However, neighboring genes can exhibit “allelic proxitropy,” where regulatory effects are shared. To identify these effects, we map QTLs on expression principal components of co-expressed neighboring genes. We discover QTLs and GWAS colocalizations missed by single-gene methods.

## Introduction

Co-expression of nearby genes is a widespread phenomenon. Empirically, across human tissues in GTEx, 13%–53% of genes have expression correlated with their neighbor.^1^ This observed correlation can result from several biological mechanisms—for instance, transcription factors co-regulating multiple genes in *trans*, shared proximal regulatory elements such as promoters and enhancers co-regulating multiple genes in *cis*, genes sharing a local chromatin state or epigenetic marks, etc.—or technical artifacts.^2^^,^^3^^,^^4^^,^^5^ Previous work has also revealed abundant allelic pleiotropy, where one variant associates with the molecular phenotypes of multiple nearby genes.^1^^,^^6^ Combined, these observations indicate that a proportion of non-coding genetic effects do not act on only one causal gene.

However, current standard approaches to understanding the effects of genetic variation still focus on one-variant-one-gene mapping approaches, despite observations of co-expression and of sharing of *cis*-regulatory mechanisms among neighboring genes.^7^ For instance, the widely utilized expression quantitative trait locus (eQTL) framework considers only one gene at a time, where the expression of a single gene is regressed on a genetic variant, resulting in an estimate of that genetic variant’s linear effect on gene expression.^8^ We hypothesize that QTL methods jointly considering neighboring genes (*cis* multi-gene QTL mapping) will improve our ability to detect and interpret the impact of common genetic variation on gene expression.

To overcome the limitations of single-gene analyses, we introduce a multi-gene QTL mapping approach—*cis*-principal-component QTLs (*cis-*pc eQTLs, or pcQTLs for brevity)—to jointly analyze clusters of neighboring, co-expressed genes across 13 human tissues from GTEx. Using this approach, we discover novel genetic effects missed by single-gene analyses. We further demonstrate improvements to colocalization with genome-wide association study (GWAS) hits, uncovering 33% additional trait-associated genetic variants missed by the traditional single-gene eQTL approach. Our results demonstrate that jointly analyzing neighboring, co-expressed genes leverages shared regulatory architecture and allelic proxitropy, improving our ability to detect and interpret genetic effects on gene expression and complex human traits.

## Methods

### Processing of GTEx RNA-seq data

Normalized expression data from GTEx v.8 for adipose (subcutaneous), adipose (visceral omentum), tibial artery, cultured fibroblasts, esophagus (mucosa), esophagus (muscularis), lung, skeletal muscle, tibial nerve, skin (not sun exposed), skin (sun exposed), thyroid, and whole blood were downloaded from the GTEx portal (https://storage.googleapis.com/adult-gtex/bulk-qtl/v8/single-tissue-cis-qtl/GTEx_Analysis_v8_eQTL_expression_matrices.tar).^9^ Tissues were chosen based on having the largest sample sizes (number of RNA sequencing [RNA-seq] samples > 400).

Expression data were then residualized on the same covariates that were used in the standard GTEx eQTL pipeline (https://storage.googleapis.com/adult-gtex/bulk-qtl/v8/single-tissue-cis-qtl/GTEx_Analysis_v8_eQTL_covariates.tar.gz): 60 probabilistic estimation of expression residuals (PEER) factors, the top 5 genotype principal components (PCs), sequencing platform, sequencing protocol, and sex.^10^

### Calling clusters of neighboring, correlated genes from RNA-seq data

To call clusters, a stepwise, iterative “sliding window” approach was used to identify stretches of neighboring, co-expressed genes (“gene clusters”). This was done for each tissue individually. Only genes with an expression level higher than the GTEx eQTL expression threshold for that tissue were considered (>0.1 transcripts per million [TPM] in at least 20% of samples and ≥6 reads in at least 20% of samples).^10^

First, for each chromosome, the Spearman's (scipy v.1.10) correlation between normalized and residualized gene expression for each pair of genes was calculated. Positive and negative correlations were both considered. We identified clusters where more than 70% of correlations between pairs of genes within the cluster were significant, such that in a cluster with *n* genes and *m* significant correlations, we require 0.7 ^∗^*C*(*n*,2) ≤ *m*.

Correlations were considered significant at *p* < 0.05 after Bonferroni correction for the total number of pairs of genes on the chromosome, *C*(*N*,2), where *N* is the total number of genes on the chromosome. This is an overly conservative threshold, as in practice our maximum cluster size ensures that we do not consider pairs of genes that are more than the maximum cluster size apart. To find these clusters, we started with a maximum cluster size of *n* = 50 genes and continued until a minimum cluster size of *n* = 2 genes. Algorithmically, assuming *N* genes total on the chromosome *G*_0_, …, *G*_*N*_, for each cluster size of *n*, we did the following:

(1) We checked whether the first *n* neighboring genes on the chromosome, *G*_0_–*G*_*n*_, were sufficiently correlated and did not belong to any existing clusters.
  a. If so, we recorded *G*_0_–*G*_*n*_ as a cluster.
(2) Sliding window 1 along the chromosome, we checked if the next *n* neighboring genes on the chromosome, *G*_1_–*G*_*n*+1_, were sufficiently correlated and did not belong to any existing clusters.
  a. If so, we recorded *G*_1_–*G*_*n*+1_ as a cluster.
(3) We continued with *G*_1_–*G*_*n*+2_, *G*_3_–*G*_*n*+3_, …, *G*_*N*__−__*n*_–*G*_*N*_. At each step, we moved the window of considered genes over by one and then checked whether the genes were sufficiently correlated and did not belong to any existing clusters.
  a. If so, we recorded the genes as a cluster.
(4) After reaching the end of the chromosome, we reduced the cluster size from *n* genes to *n* − 1 genes and then restarted at step 1. We repeated this process until the minimum window size (*n* = 2) was reached.

This approach captured genes in the largest possible cluster with significant within-cluster correlations. As false positive correlations due to cross-mappable reads were a concern, we compared the number of QTLs mapped for clusters with and without any genes containing cross-mappable 75-mers (Figure S1).^11^ As the QTL discovery rate was not statistically different between clusters with and without cross-mappable and non-cross-mappable genes, we continued to consider cross-mappable clusters for further analysis.

### Cluster enrichments for annotations

In order to evaluate whether clusters were enriched for particular annotations (such as “paralogs” or “shared GO terms”), we calculated cluster enrichments for all correlated clusters of 2, 3, 4, or 5 genes against a background of null clusters. Null clusters were all sets of 2, 3, 4, or 5 neighboring genes in each tissue that were not part of a correlated cluster in that tissue, sampled at a rate to match the relative distributions of 2, 3, 4, or 5 gene clusters in the correlated cluster set. Correlated and null clusters were assigned a boolean label: 0 if the cluster did not belong to an annotation category or 1 if the cluster did belong to an annotation category. Annotation categories are not mutually exclusive. These labels were used as the dependent variable in logistic regression to calculate enrichment odds ratios. The number of genes in the cluster and the log length of the cluster (defined by the outer edges of any transcript in the cluster in bp) were included as covariates. If the expected frequency in any cell of the contingency table was less than 5, enrichment calculations were skipped.

For each correlated and null cluster, the cluster was assigned a label of 1 for the given annotation if it fulfilled the following requirements:

(1) Shared opposite-strand promoter (bidirectional promoter): if, for any pair of genes A/B in the cluster, the 5′ end of any annotated GENCODE v.26 transcript for gene A was within 1,000 bp of the 5′ end of any annotated transcript for gene B and the genes in the pair were on opposite strands.^12^
(2) Shared same-strand promoter: if, for any pair of genes A/B in the cluster, the 5′ end of any annotated GENCODE v.26 transcript for gene A was within 1,000 bp of the 5′ end of any annotated transcript for gene B and the genes in the pair were on the same strand.^12^
(3) Overlapping opposite strand: if, for any pair of genes in the cluster, the genes overlapped, were on opposite strands, and did not share a promoter.
(4) Overlapping same strand: if, for any pair of genes in the cluster, the genes overlapped, were on the same strand, and did not share a promoter.
(5) Enhancer: if any pair of genes in the cluster shared a “genic” or an “intergenic” class enhancer with a score of >0.1 in ABC enhancer-gene predictions, for any cell type.^13^ ABC enhancer-gene connections were downloaded from the Engriez lab website (https://mitra.stanford.edu/engreitz/oak/public/Nasser2021/AllPredictions.AvgHiC.ABC0.015.minus150.ForABCPaperV3.txt.gz) and converted to hg38 with liftOver (default settings).^14^
(6) Paralogs: if any pair of genes in the cluster were listed as paralogs. Paralog information was obtained from the BioMart webtool for Ensembl 97 (https://www.ensembl.org/info/data/biomart/index.html).^15^
(7) Shared Gene Ontology (GO) term: if any pair of genes in the cluster shared a biological process (BP) GO term. GO terms for each gene were obtained from the BioMart webtool for Ensembl 97 (https://www.ensembl.org/info/data/biomart/index.html).^15^
(8) Cross CCCTC-binding factor (CTCF) peak: if a CTCF peak fell within the window defined by the outer edges of any transcript in the cluster. ENTEx CTCF chromatin immunoprecipitation (ChIP)-seq peaks from tissue-matched GTEx samples were used (https://www.encodeproject.org/entex-matrix/?type=Experiment&status=released&internal_tags=ENTEx).^16^ Specific file IDs are given on GitHub https://github.com/kal26/pcqtls/blob/main/references/ctcf_matched_gtex.txt).
(9) Cross transcriptionally associated domain (TAD) boundary: if the edge of a TAD fell within the window defined by the outer edges of any transcript in the cluster. TAD boundaries calculated from Hi-C data in GM12878 with a directionality index (DI) at a 10 kb resolution were downloaded from TADKB (http://dna.cs.miami.edu/TADKB/)^17^ and converted to hg38 with liftOver (default settings).^14^

### Cluster PCs

For each cluster of genes, principal-component analysis (PCA) (sklearn v.1.3.2, default settings) was used to find shared axes of expression variance. PCs were constructed as a linear combination of the normalized, residualized expression of the genes in the cluster.

### PC-normalized shared variance

To find the shared variance explained by the primary PC (PC1), given a cluster of *n* genes, we first summed the variance explained for each gene in the cluster by PC1:

$$\sum_{i=1}^{n}\;{{\hat{l}}_{i}}^{2}\text{,}$$

where the variance explained by PC1 for gene *i* is the squared loading ${{\hat{l}}_{i}}^{2}$ for PC1 onto that gene. We then subtracted 1 and normalized to cluster size so that shared variance was

$$\frac{\sum_{i=1}^{n}\;{{\hat{l}}_{i}}^{2}-1}{n-1}*\;100\text{.}$$

This rescales the value so that regardless of cluster size, if the expression of all genes is uncorrelated, the normalized shared variance is 0%, and if the expression of all genes is perfectly correlated, the normalized shared variance is 100%.

### Discovery and fine-mapping of pcQTLs and eQTLs

SuSiE (susieR v.0.12.35)^18^ (https://stephenslab.github.io/susieR/) was used for discovery and fine-mapping of independent loci and their 95% credible sets of variants. SuSiE was run with default parameters (default uniform prior across variants, L = 10, purity filter min_abs_corr = 0.5) and applied to individual-level genotype and expression or PC data. Only 3 single-gene expression phenotypes and 1 PC phenotype yielded the maximum of 10 credible sets, indicating that setting L = 10 was sufficient to capture the number of independent loci underlying our single-gene expression and PC phenotypes (Figure S2).

In each tissue independently, SuSiE was run on the following input phenotypes:

(1) eQTL mapping: the expression of each gene in each cluster.
(2) pcQTL mapping: each of the PCs derived from the expression of the genes in the cluster.

SuSiE was run on each PC and expression phenotype as defined above for all clusters, without pre-filtering for single-gene expression and PC phenotype significance to avoid possible thresholding effects that a false discovery rate (FDR) filter could introduce.

For both eQTL and pcQTL mapping, we considered all SNPs with a minor-allele frequency (MAF) of 0.05 or more within the variant window defined by ±1 Mb around the union (min start to max end) of the collapsed gene annotation locations of all genes in the cluster. This ensured that both eQTL and pcQTL mappings were applied to the same set of variants. Variant call files of genotype data were obtained from dbGaP (dbGaP: phs000424.v8) based on the GRCh38/hg38 reference.

### Fine-mapping calibration

To assess the calibration of pcQTL and eQTL fine-mapping, we performed a genotype-shuffling null analysis in fibroblasts. We analyzed phenotypes derived from 861 gene clusters (1,872 PC-based cluster expression phenotypes and 1,872 single-gene expression phenotypes). For each phenotype, we generated a null dataset by randomly permuting the individual-to-genotype matching while keeping phenotypes fixed, removing true genotype-phenotype associations but preserving genotype correlation structure and phenotype distributions. We then ran SuSiE fine-mapping on the shuffled-null data using the same workflow and parameters as in the real-genotype analyses (Figure S3).

### Phenotype-level permutation-based FDR

TensorQTL (https://github.com/broadinstitute/tensorqtl)^19^ was run with default settings in *cis* mode to generate permutation-based empirical *p* values and *q* values, enabling phenotype-level FDR estimation. We calculated phenotype-level FDR for three conditions.

(1) Single-gene eQTLs individually.
(2) pcQTLs individually.
(3) Both single-gene eQTLs and pcQTLs together.

Significant single-gene expression and PC phenotypes at a 5% FDR are those with *q* < 0.05.

### QTL-to-transcription start site distance

Closest gene (cluster) distance was calculated as the minimum distance from any lead variant in the credible set group to the transcription start site (TSS) of any cluster gene transcript. Note that for eQTLs, this may not be the eGene on which the eQTL was mapped. The closest gene (eGene) distance was calculated as the minimum distance from any lead variant in the credible set group to the TSS of any cluster gene that was an eGene for an eQTL credible set in the group. For genes on the positive strand, the distance was calculated as variant position − TSS, and for genes on the negative strand, the distance was calculated as TSS − variant position. The TSS of a transcript was the start, if the transcript was on the positive strand, or the end, if the transcript was on the negative strand. All transcripts associated with a gene were used.

### Posterior inclusion probability-weighted log_2_aFC

TensorQTL (https://github.com/broadinstitute/tensorqtl)^19^ was run with default settings in *cis_nominal* mode to get summary statistics (stats) for all variant-phenotype pairs. These summary stats were used to calculate the log2 allelic fold change (log_2_(aFC)). To investigate the extent to which a given pcQTL or eQTL credible set influenced each gene within a target cluster individually, we calculated the posterior inclusion probability (PIP)-weighted log_2_(aFC).^20^

An estimate of the effect of a given variant *i* on an individual gene *A* is given by the $\log_{2}\left(a{FC}_{i}^{A}\right)$ for that variant. To estimate the effect of a credible set of *j* variants, *i* = 1, …, *j*, on gene *A*, we multiplied the $\log_{2}\left(a{FC}_{i}^{A}\right)$ for each variant in the credible set by the PIP probability *PIP*_*i*_, then summed those PIP-weighted effects and divided by the sum of the PIP probabilities in the credible set:

$$\log_{2}\left(a{\text{FC}}^{A}\right)=\frac{\sum_{i=1}^{j}\;PIP_{i}\;*\;\log_{2}\left(a{\text{FC}}_{i}^{A}\right)}{\sum_{i=1}^{j}\;PIP_{i}}\text{.}$$

To get the marginal effect that a credible set discovered as a pcQTL had on the expression of gene *A*, we used the credible set of variants and PIP probabilities from the pcQTL fine-mapping and thelog_2_(aFC) for gene *A*. To get the marginal effect that a credible set discovered as an eQTL had on the expression of gene *A*, we used the credible set of variants and PIP probabilities from the eQTL fine-mapping and the log_2_(aFC) for gene *A*. This allowed us to compare the effects of pcQTL credible sets and eQTL credible sets on gene expression for each gene in a cluster.

To compare regulatory effect magnitudes for credible sets, we used maximum absolute values of |log_2_(aFC)| across all genes in a cluster.

To quantify how concentrated or distributed the regulatory effects were across the gene cluster, we calculated the coefficient of variation (CV) of absolute |log_2_(aFC)| values. A CV of 0 indicates perfectly equal effects, while higher values indicate increasingly concentrated effects on fewer genes.

### PIP-weighted variant annotations

Ensembl Variant Effect Predictor (Ensembl VEP v.114)^21^ was used to annotate each variant with a likely function. Rather than assigning a boolean label to each credible set for each annotation, we calculated an annotation probability for each credible set and annotation. The credible set annotation probability was a PIP-weighed sum over the boolean indicator for each variant in each annotation. These probabilities were used as the dependent variable in logistic regression to calculate enrichment odds ratios.

The candidate *cis*-regulatory elements (cCREs) used for credible set overlap were in matched EN-TEx tissues (https://downloads.wenglab.org/cCRE_decoration.matrix.1.gz).^22^

### Colocalization of pcQTL, eQTL, and GWAS hits

We used coloc.SuSiE (v.5)^18^^,^^23^ to colocalize pcQTL, eQTL, and GWAS signals. GWAS summary stats from a publicly available resource of 114 GWASs (for 74 distinct traits, including cardiometabolic, hematologic, neuropsychiatric, and anthropometric features from the UKBB and GIANT) harmonized and imputed to GTEx variants were used, summary stats are available on Zenodo (Zenodo: 3629742).^24^ Linkage disequilibrium (LD) reference matrices for GWAS summary data were constructed using GTEx samples with PLINK (v.1.90b7.7, default settings).^25^

In each tissue independently, for each cluster, all eQTL, pcQTL, and GWAS credible sets were colocalized with all other eQTL, pcQTL, and GWAS credible sets. We considered a GWAS hit linked to at least one single-gene eQTL in a given tissue if the GWAS hit had a posterior probability of colocalization greater than 0.75 (*PP*_H4_ ≥ 0.75) for its colocalization with any single-gene eQTL. We considered a GWAS hit colocalized with a pcQTL but not with any single-gene eQTL in a given tissue if, for at least one pcQTL, *PP*_H4_ ≥ 0.75 but for all single-gene eQTL, *PP*_H4_ < 0.75. To calculate the number of unique GWAS hits across tissues colocalized by eQTLs or by pcQTLs, we collapsed hits by GWAS variant-trait pairs. Specifically, if the same GWAS variant for the same trait colocalized with eQTLs or by pcQTL in multiple tissues, it was counted only once. However, if the same variant was associated with multiple GWAS traits, each variant-trait pair was counted separately.

### Creation of credible set groups

A custom script (https://github.com/kal26/pcqtls/blob/60f0d295d11b58bdf4035b4bbf8493828d09dd7f/workflow/scripts/group_signals.py) was used to create credible set groups. In each tissue independently, an undirected graph was constructed with each credible set as a node and an edge connecting two nodes if the probability of colocalization between those credible sets was greater than 0.75 (*PP*_H4_ ≥ 0.75). Credible set groups were the set of connected components of the graph. Credible set groups were classified as “pcQTL only” if they contained only pcQTL credible sets, “eQTL only” if they contained only eQTL credible sets, or “both” if they contained both eQTL and pcQTL credible sets. “Both” credible set groups can contain eQTLs for one or more genes in the cluster.

## Results

### Transcriptome-wide identification of clusters of co-expressed neighboring genes

We started by identifying clusters of co-expressed neighboring genes (gene clusters) across human tissues using GTEx data. We focused on 13 tissues with large sample sizes (*N* > 400), as these are best powered for eQTL analyses. As gene expression data often have global structured variance due to technical and known biological covariates, latent factor correction using tools such as surrogate variable analysis,^26^ global PCA, or PEER^27^ are often employed to improve power to discover *cis*-regulatory effects.^8^ We found that residualization of latent factors (60 PEER factors) also improved detection of co-expression of neighboring genes in GTEx data. While the un-residualized data showed frequent long-range correlations (median gene-gene distance of 40 Mb), after residualization, significant correlations are at the smaller scale of shared *cis*-regulation (median gene-gene distance of 290 kb) (Figure S4). This suggests that latent factor correction, by removing long-range interactions driven by global technical and biological covariates, increases the detection power for co-expression at a localized scale, thereby highlighting significant correlations driven by shared *cis*-regulatory mechanisms.

**Figure 1:**
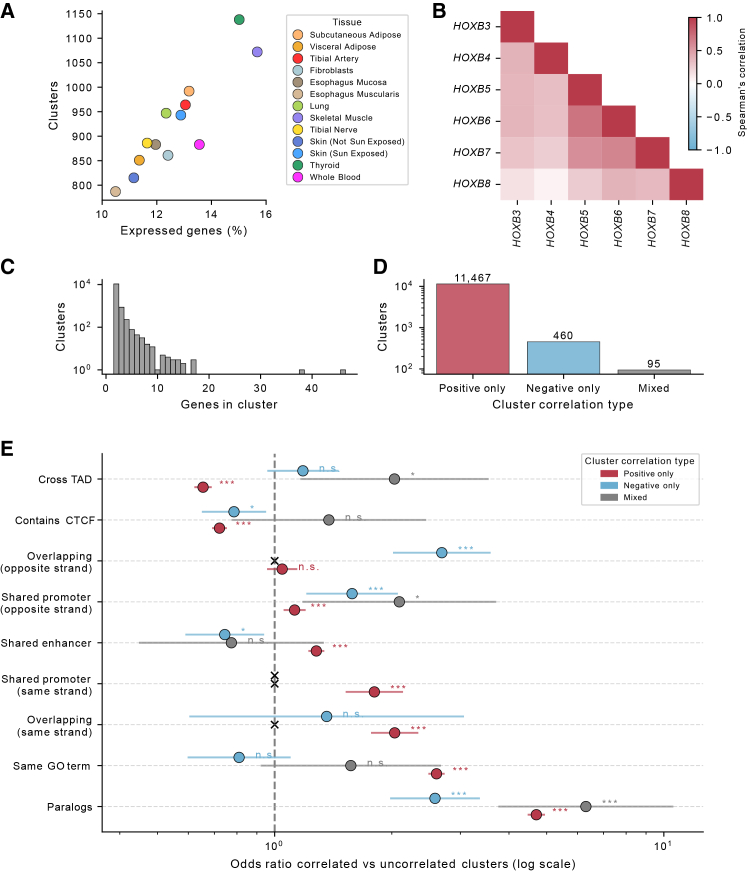
Discovery of correlated neighboring gene clusters (A) The number of clusters in each tissue vs. the percentage of all expressed genes in the tissue that are in a cluster. (B) Example heatmap of a cluster of six *HOXB* genes with correlated expression in esophagus muscularis, colored by Spearman's correlation coefficient. (C) Distribution of the number of genes per cluster. (D) Number of clusters with genes that are only positively correlated to one another, only negatively correlated, or have a mix of positive and negative correlation. (E) Enrichment of correlated clusters vs. uncorrelated null clusters (methods), split for clusters with all positive correlation, all negative correlation, or a mix of positive and negative correlation. Categories where the expected frequency was less than 5 were skipped. Error bars represent 95% confidence intervals on odds ratios for logistic regression (X: skipped, ns *p* > 0.05, ^∗^0.05 > *p* > 10^−2^, ^∗∗^10^−2^ > *p* > 10^−3^, and ^∗∗∗^*p* < 10^−3^).

We performed a transcriptome-wide analysis to identify clusters of nearby correlated genes; we did this for each tissue independently (methods). This resulted in 787–1,138 clusters across tissues—a total of 12,022 clusters—with between 10.5% and 15.7% of expressed genes in each tissue belonging to a cluster (Figures 1A, S5, and S6). The majority of clusters (89.2%) were pairs, 7.2% had 3 genes, and 3.6% had 4 or more genes (Figure 1C). We observed that genes are more frequently positively correlated than negatively correlated, although 4.6% of all clusters across tissues (*n* = 555) contained at least one pair of genes with a significant negative correlation (Figure 1D). Large clusters often represent known functionally related groups of genes, such as the Hox cluster of 6 genes on chromosome 17 or the Keratin gene cluster (with 38 genes) on chromosome 17 (Figures 1B and S6C). We further found that positively correlated gene clusters are enriched for similar biological functions and regulatory architecture. They are more likely to include paralogs, belong to the same GO term, and have shared enhancers and shared promoters (both bidirectional promoters and same-strand promoters), but they are less likely to cross a TAD boundary or contain a CTCF site. Negatively correlated clusters are depleted for shared enhancers and enriched for pairs of genes with opposite strands that overlap or share a bidirectional promoter (Figure 1E).

### A multi-gene QTL framework leverages common variation to detect shared genetic effects

**Figure 2:**
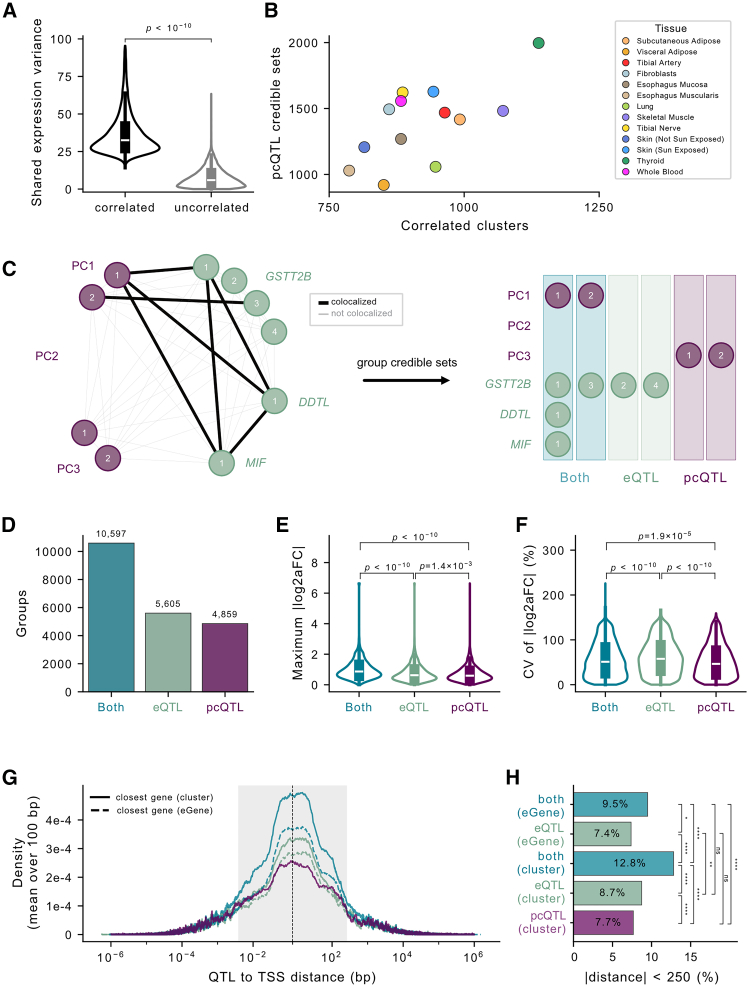
Novel pcQTL discovery (A) Normalized shared variance (methods) explained by PC1 for correlated clusters vs. neighboring genes not called as clusters in fibroblasts; the *p* value is from a two-sample *t* test. (B) Number of pcQTL credible sets vs. number of gene clusters across tissues. (C) Example of credible set groups from a three-gene cluster. Nodes are credible sets, and edges are colored according to colocalization between the credible sets. (D) Number of credible set groups of each type across all clusters. (E) The maximum PIP-weighted |log_2_(aFC)| for any single gene in the cluster; *p* values are from a two-sample *t* test. (F) The CV of PIP-weighted |log_2_(aFC)| across genes in the cluster (quantifying how concentrated vs. distributed the effect is); *p* values are from a two-sample *t* test. (G) For each credible set group, the minimum credible set lead variant-TSS distance to any gene in the cluster, and for credible set groups containing an eQTL credible set, the minimum credible set lead variant-TSS distance to any eGene in the cluster (gene with a significant eQTL credible set in the group). The gray shaded area is |distance| < 250 bp. Lines are colored by credible set group type. (H) Proportion of lead variants with |distance| < 250 bp for credible set group-type and distance-type categories in (G). *p*values are from Fisher’s exact test (ns *p* > 0.05, ^∗^0.05 > *p* > 10^−2^, ^∗∗^10^−2^ > *p* > 10^−3^, and ^∗∗∗^*p* < 10^−3^).

To allow us to jointly consider gene clusters in QTL analysis, we first calculated cluster PCs from the normalized gene expression data for each gene cluster (methods). As expected for PCA on pre-selected correlated variables, variables have some proportion of shared variance. To summarize the degree to which gene clusters’ expression variance is shared, we calculated the average variance explained across genes in each cluster by PC1, normalized by cluster size such that a cluster with completely correlated gene expression for all genes would have a shared variance of 100% and a cluster with completely uncorrelated expression would have a shared variance of 0%. Clusters have significantly higher (*p* < 10^−10^) shared variance (36.6%) than non-correlated neighbor gene pairs (7.6%) (Figures 2A and S7). We then use PCs as the dependent variable in QTL mapping (pcQTL). This pcQTL framework allows us to estimate the effect of genetic variants on a shared axis of expression variance across the gene cluster.

We mapped and fine-mapped pcQTLs with SuSiE, using all PCs from each gene cluster as input phenotypes (PC1, PC2, …, PC(*n*) for an *n*-gene cluster). Across tissues, we identified 920–1,997 pcQTL credible sets per tissue (Figures 2B, S2, and S8). For comparison, we ran SuSiE analogously using the expression of each gene within a cluster as the phenotype. A permutation-based, phenotype-level FDR analysis confirmed that any PC or single-gene expression phenotype with one or more mapped credible sets was significant at a 5% FDR (Figure S9).

In order to identify how many of the pcQTLs are novel, we colocalized each pcQTL credible set to every eQTL credible set for each cluster (Figure 2C). We found that 98% of credible set groups with two or more eGenes were also tagged by a pcQTL (Figures S10 and S11). pcQTL credible sets that did not colocalize to any eQTL credible sets for any gene in the cluster were considered novel. Across tissues, we found 4,859 novel pcQTLs (26.7% of all pcQTL credible sets) that were not discovered by any single-gene eQTL analysis (Figures 2D and S8).

To investigate why the pcQTL approach identifies novel signals, we calculated the log_2_(aFC) effect of each pcQTL credible set on the expression of each gene within its cluster (methods). This gives us an estimate of the effect of a given QTL on the expression of each gene individually. Compared to eQTLs, we found that novel pcQTLs from PC1 signals had a smaller effect on any single gene in their cluster (*p* = 1.4 × 10^−3^) (Figure 2E) but that effects were more distributed across genes (*p* < 10^−10^) (Figures 2F and S12), indicating that, as expected, PC1s summarized smaller-size effects distributed across all genes in their correlated cluster.

### Multi-gene pcQTLs colocalize with new GWAS hits

**Figure 3:**
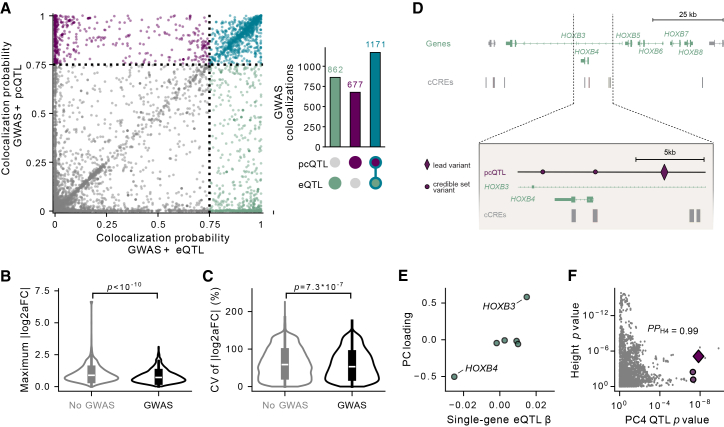
pcQTL colocalization with complex-trait-associated variants (A) Maximum posterior probability of a shared causal variant (*PP*_H4_) underlying the GWAS hit and any pcQTL credible set for the cluster vs. for any eQTL credible set in the cluster. *PP*_H4_ = 0.75 is shown as a dotted line. UpSet plot (right): total GWAS colocalizations across all clusters and tissues. (B) For all credible set groups, the maximum PIP-weighted |log_2_(aFC)| for any single gene in the cluster, split by whether or not the credible set group colocalizes with a GWAS hit; the *p* value is from a two-sample *t* test. (C) For all credible set groups, the CV of PIP-weighted |log_2_(aFC)| across genes in the cluster (quantifying how concentrated vs. distributed the effect is), split by whether or not the credible set group colocalizes with a GWAS hit; the *p* value is from a two-sample *t* test. (D) *HOXB3*, *HOXB4*, *HOXB5*, *HOXB6*, *HOXB7*, and *HOXB8* gene cluster and cCRE regulatory elements in esophagus muscularis; close-up of region with fine-mapped novel PC4 pcQTL credible set. (E) Marginal effect of pcQTL on each eGene vs. PC4 loading onto each eGene. (F) Nominal *p* values for height GWAS and PC4. Posterior probability of colocalization between the GWAS hit and the pcQTL credible set *PP*_H4_ is 0.99.

Most trait-associated variants in GWASs lie in non-coding regions and are thought to affect traits by regulating gene expression, yet identified eQTLs and sQTLs only colocalize with a small percentage of GWAS hits (47% linked to target genes in GTEx across all tissues).^24^ We sought to investigate whether our newly discovered pcQTLs can help explain additional GWAS hits. To do this, we colocalized our pcQTLs with GWAS hits for 74 traits, including cardiometabolic, hematologic, neuropsychiatric, and anthropometric features from the UKBB and GIANT.^24^ For comparison, we also colocalized GWAS hits and eQTLs for each gene in each cluster. Using single-gene eQTL mapping alone, 2,033 GWAS hits could be linked to at least one single-gene eQTL in a cluster in a given tissue (representing 792 unique GWAS hits across tissues). With multi-gene mapping on cluster PCs, an additional 677 GWAS hits were colocalized with a pcQTL but not with any single-gene eQTLs with a cluster in a given tissue (representing 176 unique GWAS hits) (Figures 3A and S13; Table S1). This represents a 33% increase in colocalizations (22% increase in unique GWAS hits) compared with single-gene eQTLs alone.

Consistent with evidence that large-effect QTL variants are less likely to colocalize with GWAS hits because stronger regulatory effects at crucial genes tend to be purged by negative selection,^28^ we found that larger-effect QTLs in our analysis were less likely to colocalize with GWAS hits (*p* < 10^−10^) (Figure 3B). Previous work has demonstrated that GWAS hits tend to have lower allele frequencies and relative depletion near gene TSSs relative to single-gene eQTLs^13^; pcQTLs also share these properties (Figures 2G, 2H, and S14). QTLs with smaller effects on any single gene but with effects distributed across multiple genes (quantified by lower CV |log_2_(aFC)|; methods) showed higher rates of colocalization (*p* = 7.3 × 10^−7^; Figure 3C) and were more likely to be pleiotropic (*p* = 0.023; Figure S15). These weak, multi-gene effects are precisely the type of signal that pcQTL mapping can capture more effectively.

An example of one such novel pcQTL credible set colocalization illustrates how PCs can boost power by summarizing distributed effects across genes. The colocalization is between PC4 for a 6-gene *HOXB* cluster (*HOXB3*, *HOXB4*, *HOXB5*, *HOXB6*, *HOXB7*, and *HOXB8*) in esophagus muscularis tissue and a GWAS hit for height (Figure 3D). Although the *HOXB* transcription factors play a well-established role in development^29^ and single-gene eQTL analysis maps an eQTL for *HOXB3*, this single-gene *HOXB3* eQTL did not colocalize with the height GWAS hit (*PP*_H4_ = 4.7 × 10^−5^). When we instead used PC4 for pcQTL mapping, we detected a novel locus that colocalized with the GWAS hit (*PP*_H4_ = 0.99) (Figure 3F).

Based on PC loadings, PC4 primarily captured inverse variation between *HOXB3* and *HOXB4* (Figure 3E). The two genes are positively correlated (Figure 1B), but this positive correlation is summarized in the first 3 PCs, allowing PC4 to capture a subtler inverse effect. Fine-mapping established a three-variant credible set for the pcQTL (Figure 3D), and while the credible set variants have below-threshold nominal *p* values for some genes individually, none reach significance on their own (Figure S16). One variant of the credible set overlaps a cCRE region in esophagus muscularis (Figure 3D), and notably, enhancer-promoter links from the ABC model indicate that the regulatory element acts as both the promoter of *HOXB4* and an enhancer for *HOXB3*, the same two genes that load onto PC4. Multi-gene pcQTL analysis can reveal this kind of joint regulatory effect, which would be missed by single-gene analysis, providing potential insights into loci that manifest as coordinated changes in gene expression relationships rather than strong individual gene effects.

A second novel pcQTL colocalization further demonstrates how pcQTL analysis can reveal loci missed by single-gene eQTLs. A pcQTL from PC2 for a two-gene cluster of interleukin 18 (IL-18) receptor complex genes (*IL18RAP* and *IL18R1*) in sun-exposed skin tissue colocalizes with a pleiotropic GWAS hit for eczema (*PP*_H4_ = 0.87), dermatitis (*PP*_H4_ = 0.87), lymphocyte count (*PP*_H4_ = 0.78), and inflammatory bowel disease (IBD) (*PP*_H4_ = 0.76) (Figure S17). The IL-18 cytokine is a pro-inflammatory factor, previously implicated in a wide range of inflammatory skin disorders, with elevated IL-18 levels observed in patients with eczema, dermatitis, or IBD.^30^^,^^31^^,^^32^ As in the previous example, single-gene analysis maps individual eQTLs, one for *IL18RAP* and one for *IL18R1*, but neither colocalizes with the inflammatory disease GWAS signals (max *PP*_H4_ = 7.6 × 10^−4^; Figure S17).

The novel pcQTL fine-maps to an 87-variant credible set in strong LD (LD *r*^2^: 0.86–1.0), with variants that overlap the *IL18RAP* promoter and five ABC enhancer elements linked to both *IL18R1* and *IL18RAP*. The complex-trait associations may result from only a single causal variant or from multiple causal variants on the haplotype,^33^ but only by considering the variants’ combined effect on both genes are we able to map the pcQTLs. These examples highlight how complex-trait associations can emerge from coordinated effects on co-regulated genes—effects that would be overlooked when genes are analyzed in isolation but become detectable through multi-gene approaches such as pcQTLs.

## Discussion

eQTL studies have focused on single-gene-at-a-time analyses despite evidence of molecular pleiotropy, where variants impact the molecular function of multiple genes. To detect such pleiotropic variants, previous work has primarily focused on mapping single-gene eQTLs and reporting instances where a variant is independently associated with multiple genes rather than leveraging shared effects.^1^^,^^6^ We demonstrate that when we move from considering genes as discrete functional units to jointly considering multiple, neighboring genes, we detect novel QTLs and new colocalizations with complex-trait-associated variation.

Because multi-gene regulatory effects can operate through diverse causal mechanisms—affecting one gene or multiple genes simultaneously or creating gene-gene interactions—an additional layer of complexity exists for the interpretation of pcQTLs. These complexities can be hidden when analyzing single-gene eQTLs when each gene is considered in isolation. We expect that using a *cis-*multi-gene approach will clarify the existence of more complex mechanisms, presenting opportunities for expanding experimental validation and functional follow-up approaches when studying genetic risk factors.

In our study, pcQTLs represent a proof-of-concept methodology for *cis-*multi-gene eQTL mapping to uncover shared local gene regulation. This approach builds on a wide range of previous work where grouping of related phenotypes has been a versatile approach to uncover novel signals, such as the grouping of GWAS traits to leverage phenotypic pleiotropy,^34^^,^^35^ the grouping of spatially correlated epigenomic signals,^36^^,^^37^ or the grouping of co-expressed genes genome wide to capture *trans*-network and pathway effects.^38^^,^^39^^,^^40^^,^^41^ By focusing specifically on neighboring genes, pcQTLs enrich for shared *cis*-regulation. Further work will be required to determine which clustering methods and statistical tools best capture shared *cis*-regulation, and different aspects of multi-gene QTL discovery may require different strategies. However, the dramatic increase in discoveries and complex-trait-associated variation even with a methodologically straightforward approach such as pcQTLs highlights that leveraging shared *cis*-regulation and subsequent co-expression of neighboring genes is an important aspect of QTL mapping that can elucidate new trait and disease biology.

Combined, this approach recognizes the complexity of gene regulation, leveraging the expression correlations among nearby genes to capture novel signals of molecular pleiotropy. However, as molecular pleiotropy can describe both local and distal effects, we classify variants with effects on multiple, proximal genes as having molecular proxitropy. We expect that future studies will benefit from moving from a classical genetics view of a causal variant impacting a single causal gene to one integrating the complexity of local regulation.

## Data and code availability

The accession number for the correlated gene clusters, PC and expression inputs for QTL mapping, eQTL and pcQTL summary stats, eQTL and pcQTL SuSiE credible sets, QTL-QTL and QTL-GWAS colocalizations, and credible set groups reported in this paper is [Zenodo]: 18320563.

Scripts used to call clusters, pipelines for data processing, links to all annotation files, and notebooks to generate all figures are available at https://github.com/kal26/pcqtls.

## Acknowledgments

We thank the donors and their families for their generous gifts of biospecimens to the GTEx research project. The GTEx project was supported by the Common Fund of the Office of the Director of the 10.13039/100000002National Institutes of Health (NIH) (http://commonfund.nih.gov/GTEx). Additional funds were provided by the 10.13039/100000054National Cancer Institute (NCI); the 10.13039/100000051National Human Genome Research Institute (NHGRI); the 10.13039/100000050National Heart, Lung, and Blood Institute (NHLBI); the 10.13039/100000026National Institute on Drug Abuse (NIDA); the 10.13039/100000025National Institute of Mental Health (NIMH); and the 10.13039/100000065National Institute of Neurological Disorders and Stroke (NINDS). This research was supported by NIH grants R01MH12524, U01AG072573, and U01HG012069 to S.B.M. K.A.L. is supported by the Stanford Genome Training Program (SGTP; NIH/NHGRI T32HG000044). T.G. is supported by the Knight-Hennessy Scholars fellowship. The funders had no role in the study design, data collection and analysis, decision to publish, or preparation of the manuscript.

## Author contributions

S.B.M., T.G., D.N., and K.A.L conceived and designed the study. T.G. designed the cluster-calling algorithm. K.A.L. performed the remainder of the analyses. S.B.M., T.G., D.N., and K.A.L. wrote the manuscript.

## Declaration of interests

S.B.M. is on the scientific advisory board of MyOme and PhiTech.

## Declaration of generative AI and AI-assisted technologies in the writing process

During the preparation of this work, the authors used OpenAI-o4 and Claude-4 to improve language clarity and figure aesthetics. After using this tool/service, the authors reviewed and edited the content as needed and take full responsibility for the content of the publication.

## Web resources

Zenodo, https://zenodo.org/records; dbGaP, https://dbgap.ncbi.nlm.nih.gov/home/

## Supplemental information

Document S1. Figures S1–S17

Table S1. GWAS colocalizations across tissues and traits The total number of GWAS hits near clusters is the number of unique GWAS lead variants fine-mapped within 1MB of a gene cluster. The total number of GWAS hits near clusters per tissue is the number of unique GWAS lead variants fine-mapped within 1MB of a gene cluster in each tissue. The number of GWAS hits colocalized per GWAS trait in each tissue is given for eQTLs and for novel pcQTLs (pcQTLs not colocalized by an eQTL).

Document S2. Article plus supplemental information
