# Supplementary material for: Focus on single-gene effects limits discovery and interpretation of complex-trait-associated variants
Source: Am J Hum Genet. 2026 Mar 23;113(4):842–51. doi: 10.1016/j.ajhg.2026.02.022 (PMC13087463; doi:10.1016/j.ajhg.2026.02.022)
Supplement: Document S1. Figures S1–S17 [file mmc1.pdf]

The American Journal of Human Genetics, Volume *113*

Supplemental information

Focus on single-gene effects limits discovery and interpretation of complex-trait-associated variants

Kathryn A. Lawrence, Tamara Gjorgjieva, Daniel Nachun, and Stephen B. Montgomery

Supplemental Figures

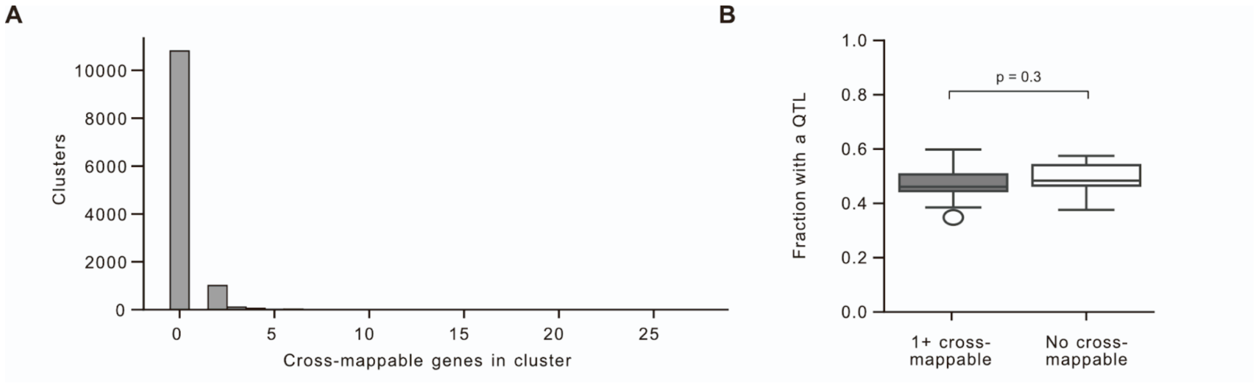

Figure S1: Cross-mappability of clusters.
(A) Histogram of the number of genes in each cluster with more than 100 cross-mappable 75-mers.
(B) Fraction of gene clusters with at least one significant QTL for clusters with at least one cross-mappable gene pair or with no cross mappable gene pairs; the *p* value is from a two-sample *t* test.

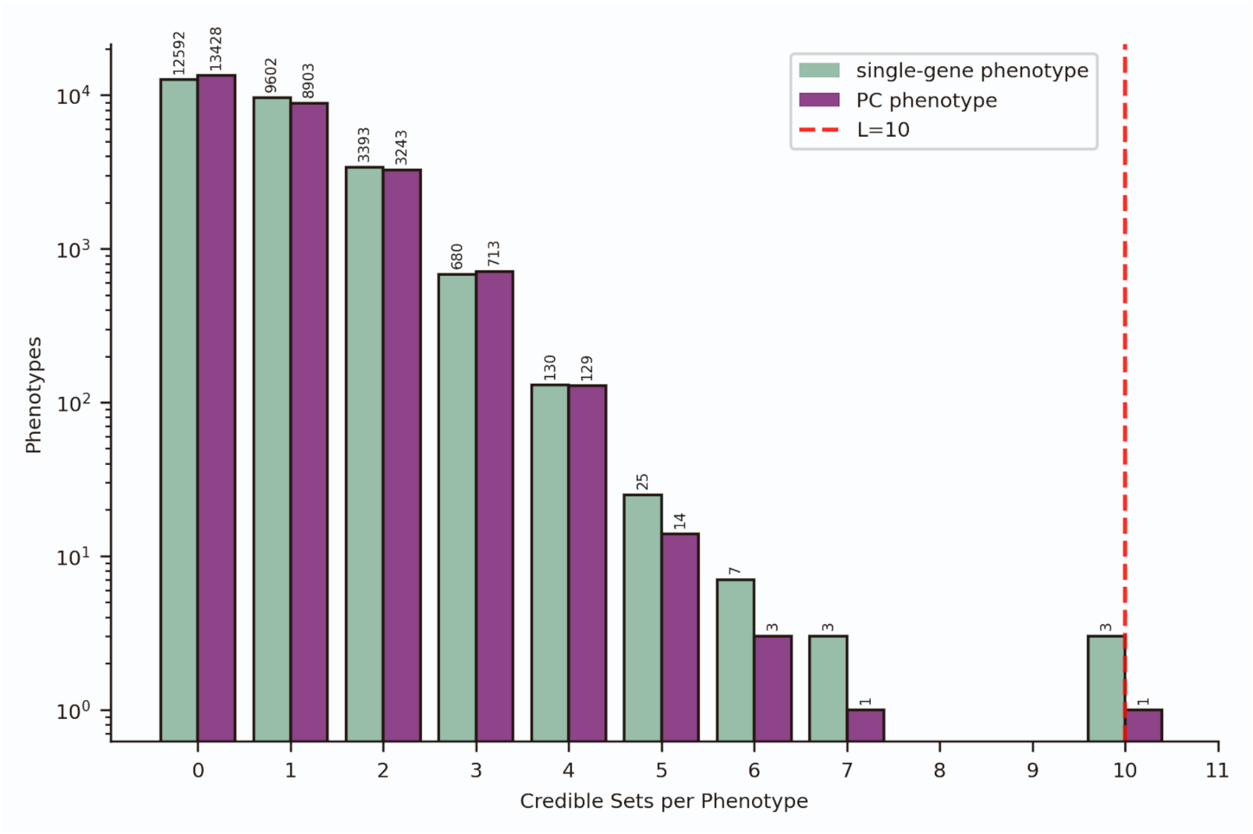

Figure S2: Credible sets per phenotype.
Number of credible sets mapped per PC phenotype (pcQTL) and expression phenotype (eQTL). The dotted red line shows 10 credible sets, the maximum number of credible sets possible for a single phenotype with SuSiE run with L = 10.

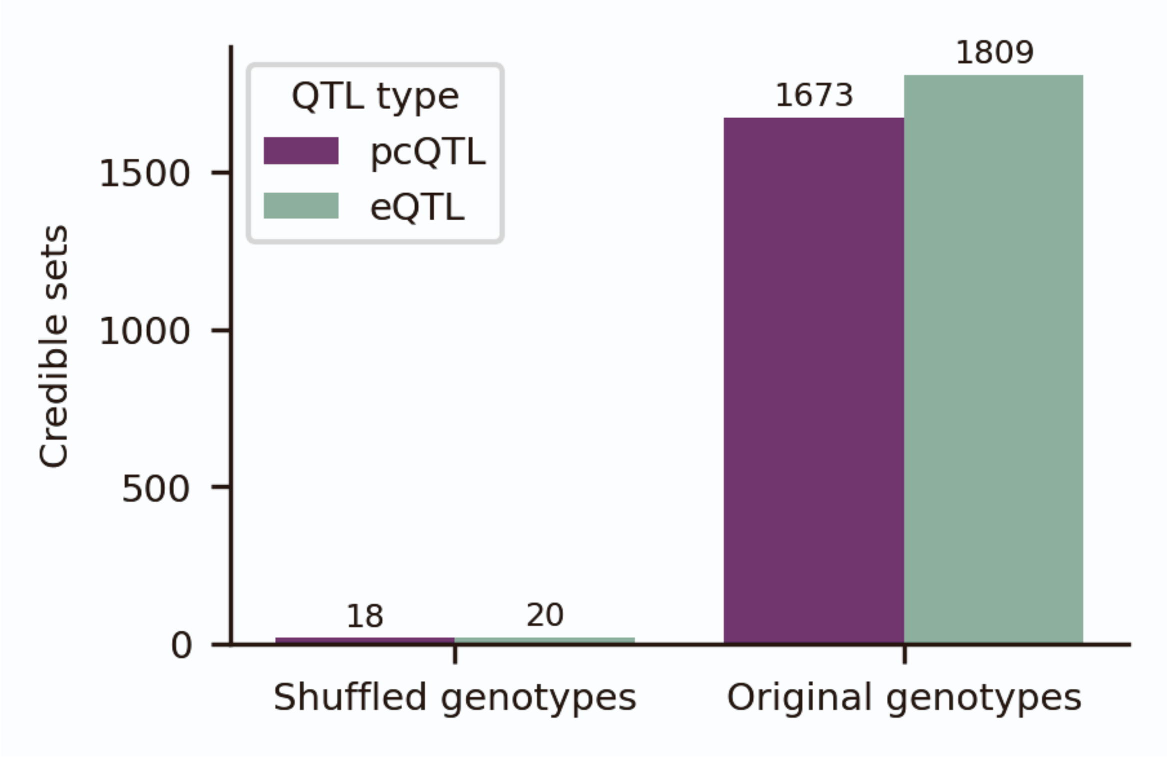

Figure S3: pcQTL mapping null with shuffled genotypes.
For each single-gene expression and PC phenotype for clusters in fibroblast tissue, the genotypes at each variant position were shuffled. SuSiE was then run with the shuffled genotype and with the real genotypes to map pcQTLs and eQTLs. PC phenotypes were not significantly more likely to map false positive pcQTLs with the shuffled genotype than single-gene expression phenotypes were to map false positive eQTLs with the shuffled genotype and eGenes; Fisher's exact $p = 1.0$.

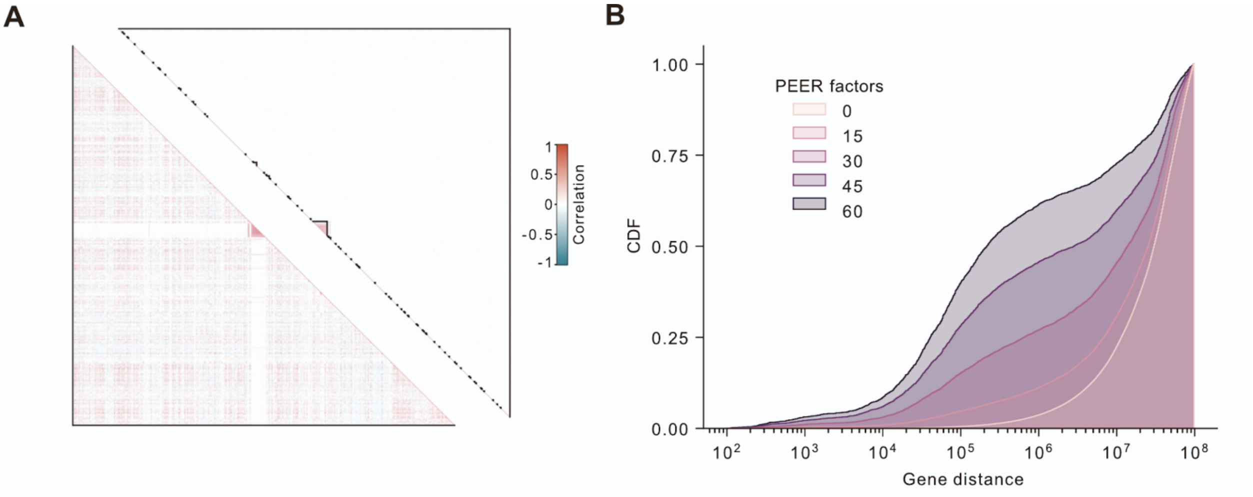

Figure S4: PEER factor residualization's effect on correlation.

(A) Correlation matrix of gene expression in chromosome 17 for sun exposed skin; Spearman's correlation before (lower) and after (upper) residualization with 60 PEER factors. Correlations with Bernoulli-corrected $p < 0.05$ are shown. Clusters are outlined in black on the upper matrix.

(B) CDF of pairwise gene distances of genes with significantly correlated gene expression profiles after residualization with 0, 15, 30, 45, and 60 PEER factors.

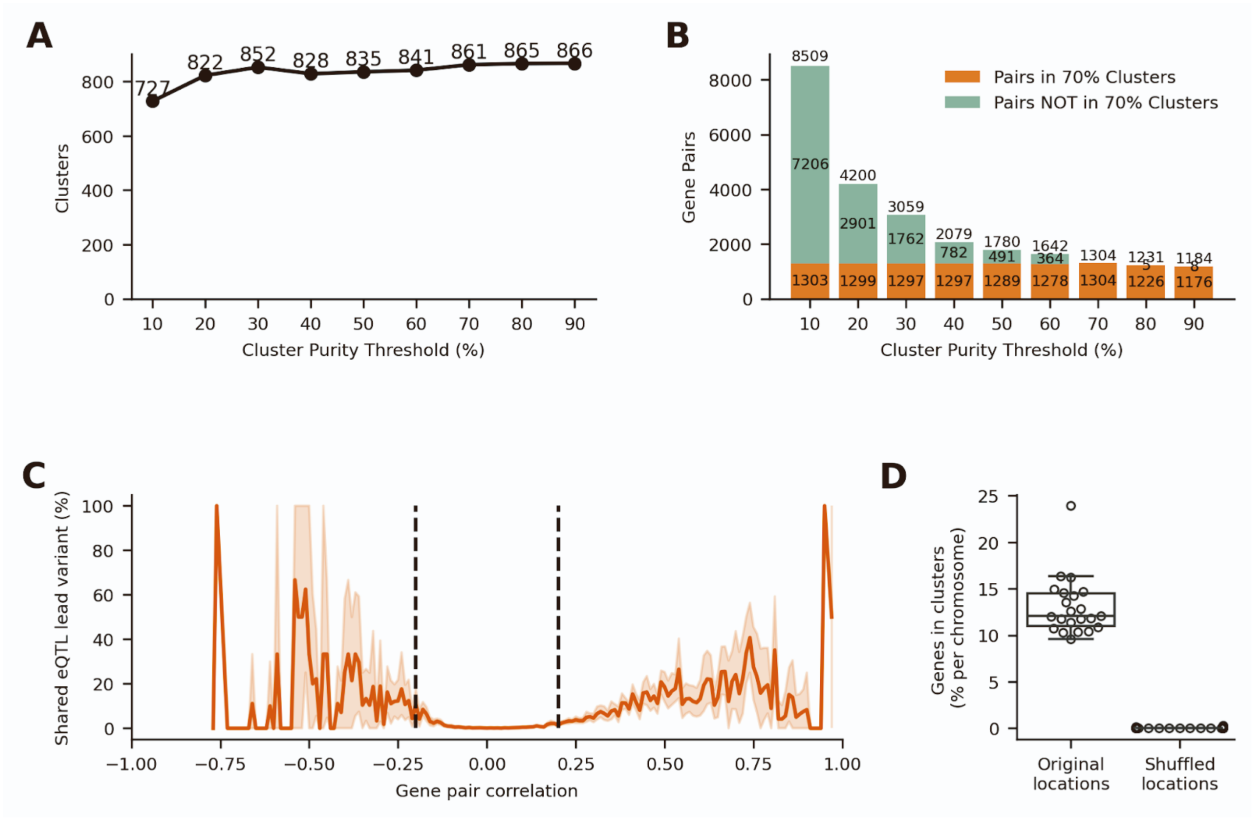

Figure S5: Clustering sensitivity to hyperparameters.

(A) Number of clusters in fibroblasts vs clustering purity thresholds from 10–90%.

(B) Gene pairs in fibroblasts clusters vs clustering purity thresholds from 10–90%, colored by whether the gene-pair was included in clusters at a 70% purity threshold used in this analysis.

(C) Fraction of gene-pairs with a shared single-gene eQTL lead variant vs gene-pair correlation for all neighboring gene pairs. The dotted lines represent the mean correlation cutoffs used for our clusters.

(D) Fraction genes in clusters called on expression data for fibroblasts with relative gene locations shuffled. Each point is a chromosome.

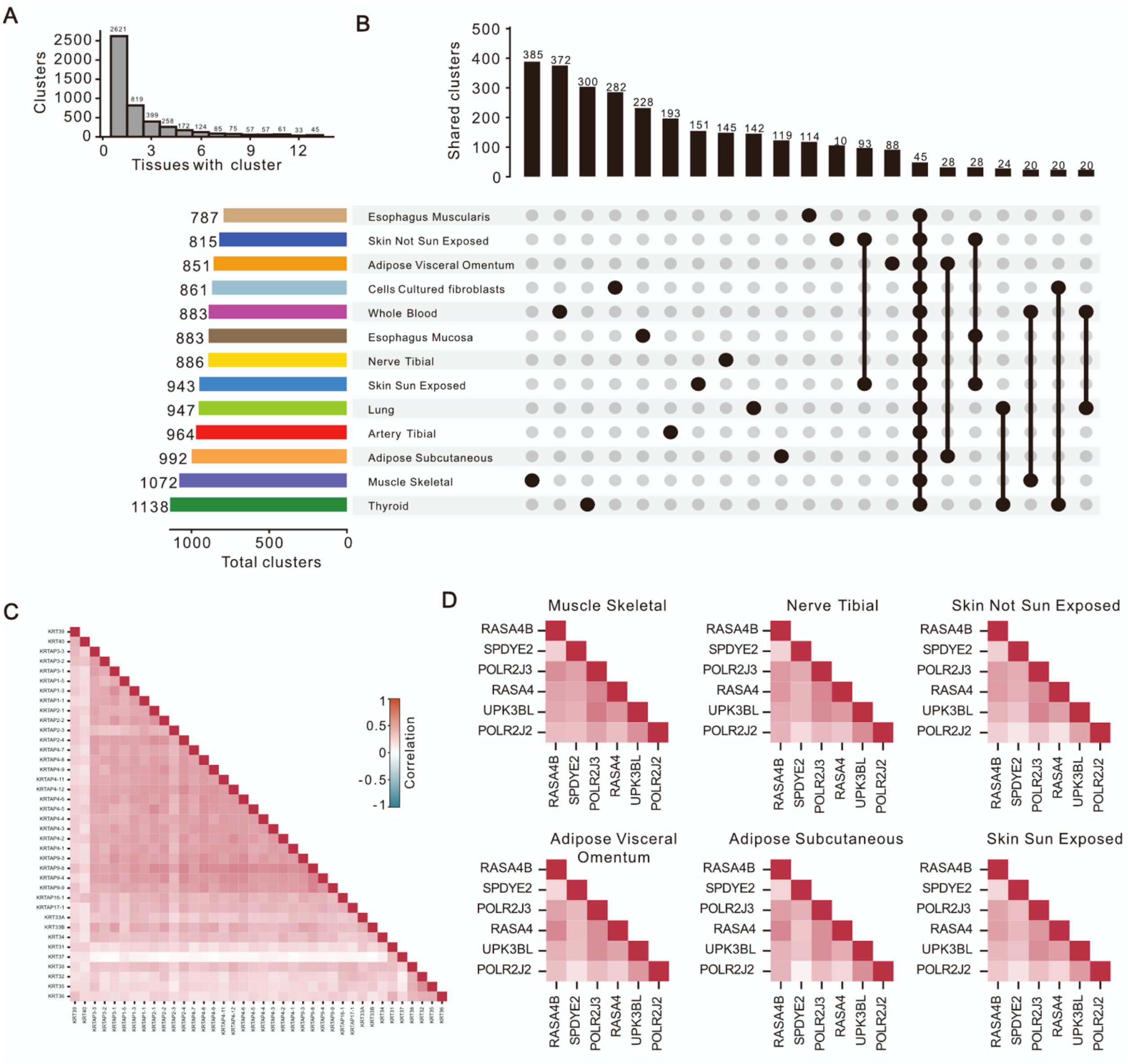

Figure S6: Tissue specificity of clusters.
(A) Histogram of the number of tissues a cluster is in.
(B) Upset plot detail of which tissues a cluster is shared across for all tissue combinations with 20 or more clusters.
(C) A tissue-specific thirty-eight gene cluster on chromosome 17 from sun-exposed skin gene expression. Color is Spearman’s correlation of expression.
(D) A six gene cluster on chromosome 6, shared across 6 tissues. Color is Spearman’s correlation of expression.

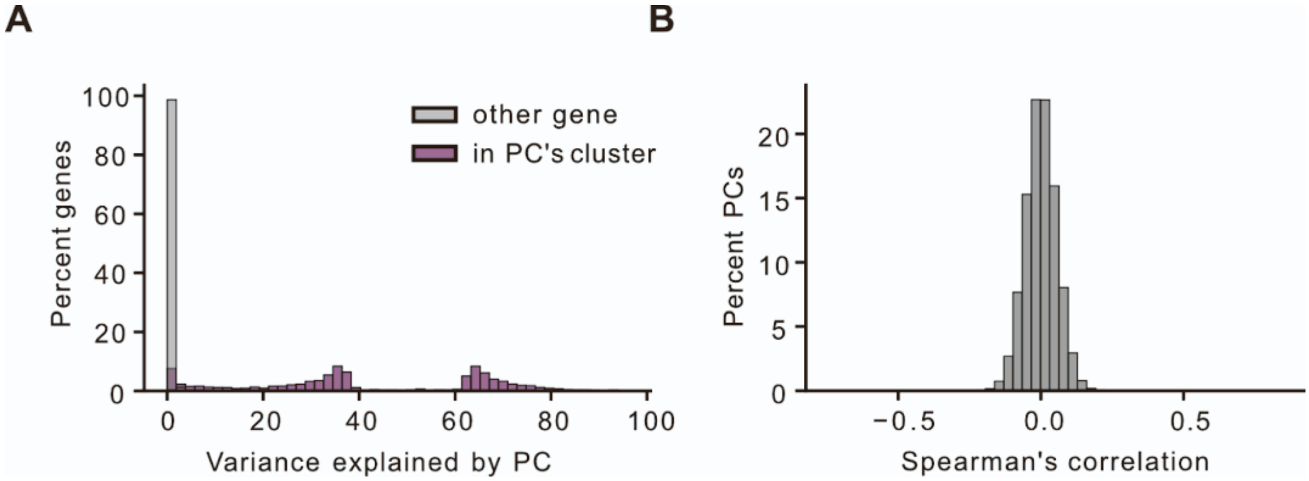

Figure S7: Local PC properties.
(A) Variance explained by each PC phenotype for all genes split by whether or not the gene is in the cluster whose expression was used to calculate the PC.
(B) Distribution of Spearman's correlation from each PC to all other PCs for all fibroblast PCs.

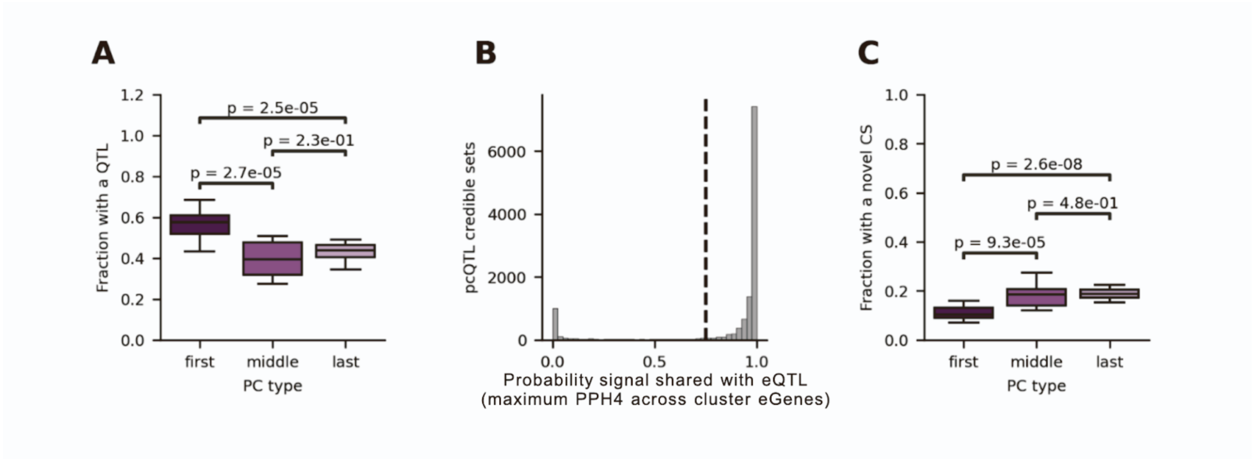

Figure S8: pcQTL discoveries by PC order.
(A) The fraction of PC phenotypes across tissues with at least one significant QTL split into the primary (first) PC, the last PC, and all other PCs (middle); *p* values are from a two-sample *t* test.
(B) Distribution of the maximum PP_{H4} colocalization probability for each pcQTL credible set with any cluster eQTL credible set. Dotted line $PP_{H4} = 0.75$.
(C) The fraction of PC phenotypes across tissues with at least one novel significant QTL split into the primary (first) PC, the last PC, and all other PCs (middle); *p* values are from a two sample *t* test.

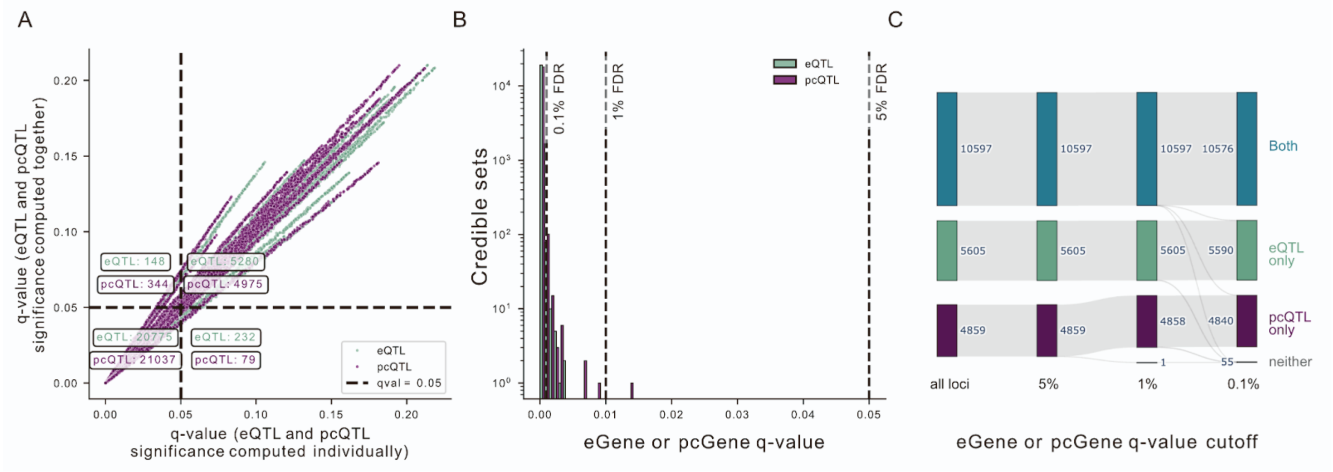

Figure S9: Phenotype filtering by FDR prior to fine-mapping.
(A) *q* values from permutation mode of tensorQTL for single-gene expression phenotypes and PC phenotypes each run together, vs each run individually. Boxes give the number of single-gene expression and PC phenotypes in each quadrant, with significance cutoffs of $q < 0.05$, a 5% FDR.
(B) For each single-gene expression or PC phenotype with a significant fine-mapped SuSiE credible set, the distribution of tensorQTL permutation *q* values from combined mapping on single-gene expression and PC phenotypes together. Dotted lines represent *q* values for 5%, 1% and 0.1% FDR cutoffs. All single-gene expression and PC phenotypes with a credible set would pass 5% FDR. All single-gene expression phenotypes and all but one PC phenotype with a credible set would pass 1% FDR.
(C) Discovery of credible-set-groups at various combined *q* values thresholds in a two-step QTL mapping process. At each *q* values cutoff, we subset to only credible sets mapped on single-gene expression and PC phenotypes with $q <$ cutoff. Then, pcQTL and eQTL credible sets are again grouped by colocalizations and the groups are classified according to whether the contain only pcQTL credible sets, only eQTL credible sets, both eQTL and pcQTL credible sets, or no longer contain any credible sets.

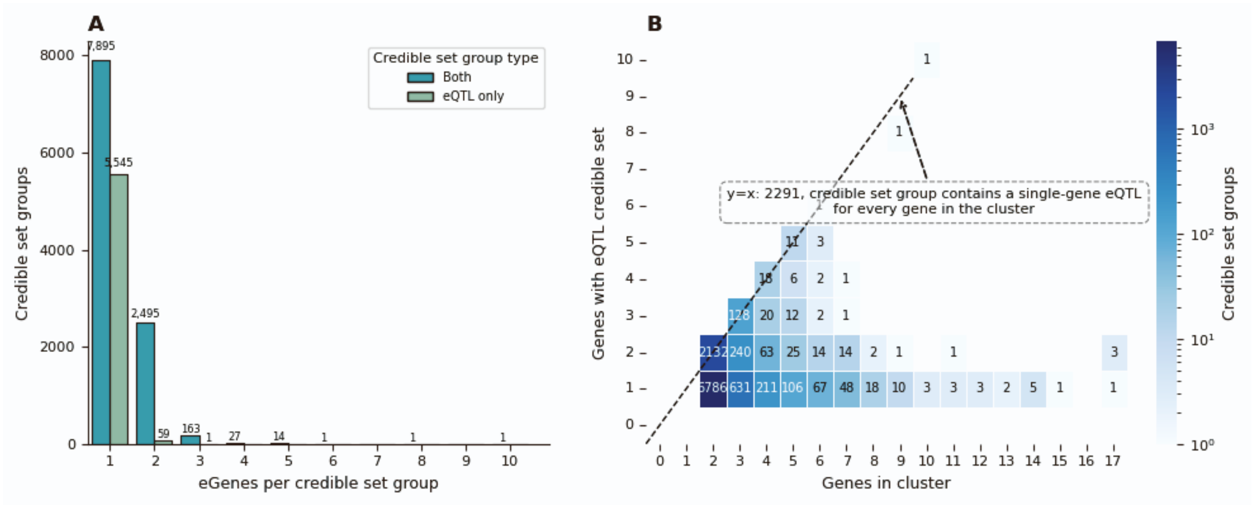

Figure S10: eGene colocalization.
(A) Number of eGenes colocalized in each credible set group split by whether the group is eQTL only or both a pcQTL and eQTL.
(B) Distribution of the number of genes in a cluster vs the number of genes that are single-gene eQTLs for credible set groups that contain both eQTLs and pcQTLs. 2,291 credible set groups contain a single-gene eQTL for every gene in the cluster.

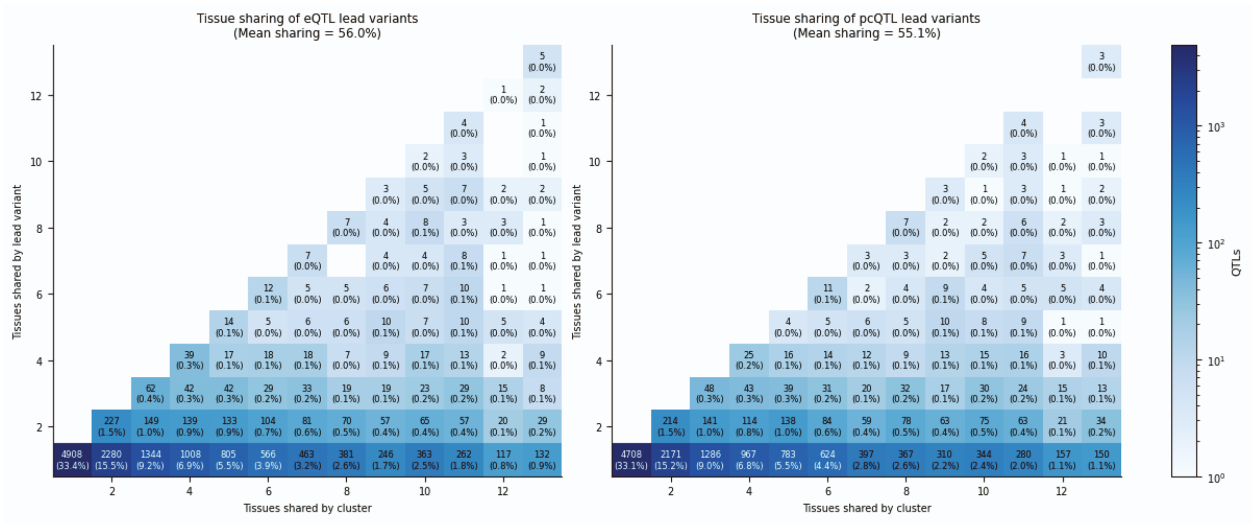

Figure S11: Lead variant sharing.

For eQTL lead variants (left) and pcQTL lead variants (right), the number of tissues that share the lead variant vs the number of tissues in which the QTL's cluster was mapped. For each lead variant, percent sharing is computed as the number of tissues with the shared lead variant divided by the number of tissues with the shared cluster multiplied by 100. The mean sharing is the mean percent sharing across lead variants. pcQTLs are slightly, but statistically significantly, more tissue specific, with lower sharing; Mann-Whitney *U* test $p = 0.011$.

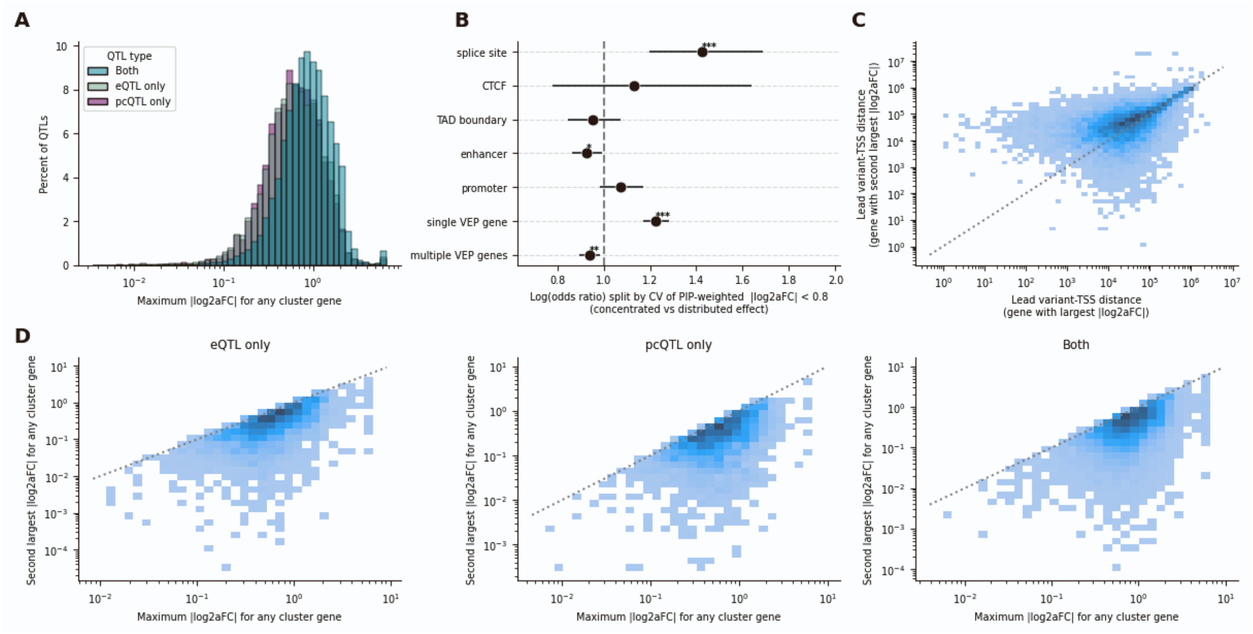

Figure S12: Distribution of single-gene $\log_2$aFC effects.

(A) Maximum PIP-weighted $|\log_2 aFC|$ for any gene in a cluster, split by if the QTL is discovered as both a pcQTL and eQTL, an eQTL only, or a pcQTL only. Maximum $|\log_2 aFC|$ for credible sets discovered as pcQTLs only or eQTLs only were not significantly different (p = 0.333) from each other. Maximum $|\log_2 aFC|$ for credible sets discovered as both pcQTLs and eQTLs were significantly higher than those discovered as pcQTLs only ($p < 10^{-10}$) or eQTLs only ($p < 10^{-10}$); p values are from Mann-Whitney U test.

(B) Annotation enrichments for PIP-weighted variant effect predictor categories for QTL credible sets split by if the CV of PIP-weighted $|\log_2 aFC| < 0.8$. Error bars are 95% confidence intervals on odds ratios for logistic regression (*$0.05 > p > 10^{-2}$, **$10^{-2} > p > 10^{-3}$, and ***$p < 10^{-3}$).

(C) The distance from the lead variant of the QTL credible set to the TSS of the gene with the second largest $|\log_2 aFC|$ vs the distance from the lead variant to the TSS of the gene with the largest $|\log_2 aFC|$ gene.

(D) The second largest PIP-weighted $|\log_2 aFC|$ for each QTL on any eGene in a cluster vs the largest PIP-weighted $|\log_2 aFC|$ on any eGene in a cluster, split by if the QTL is discovered as both a pcQTL and eQTL, an eQTL only, or a pcQTL only.

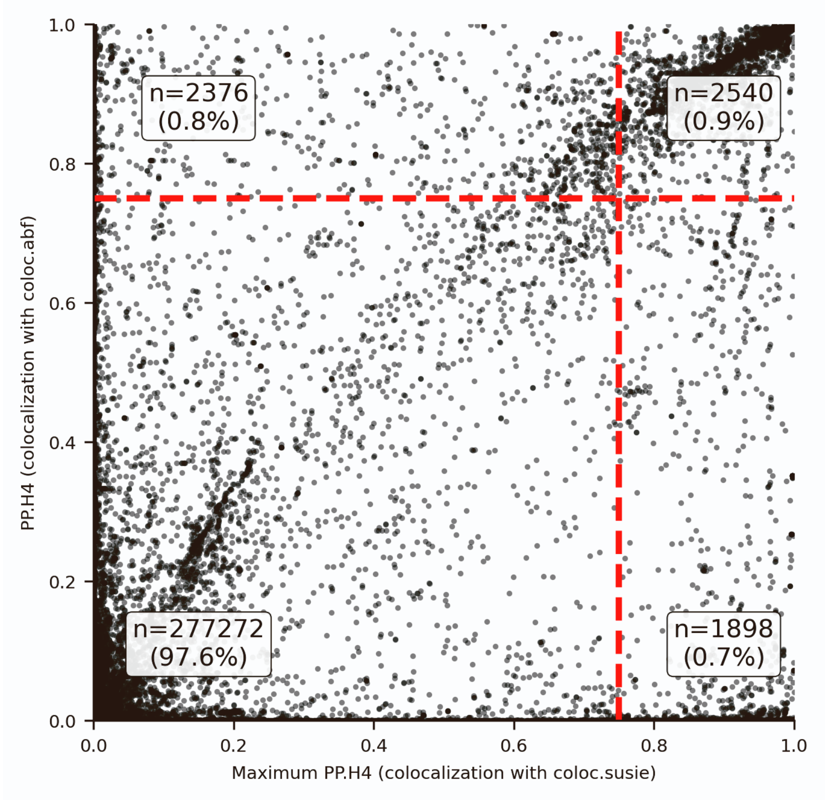

Figure S13: Single-causal variant colocalization.

For each single-gene expression phenotype or PC phenotype and GWAS trait, posterior probability of colocalization (PP_{H4}) between GWAS and QTL phenotypes assuming a single-causal variant (coloc.abf) vs maximum PP_{H4} over all credible sets for colocalization with run SuSiE, allowing for multiple causal variants (coloc.susie). Dotted line PP_{H4} = 0.75. Boxes show the number and percent of GWAS-phenotype pairs in each quadrant.

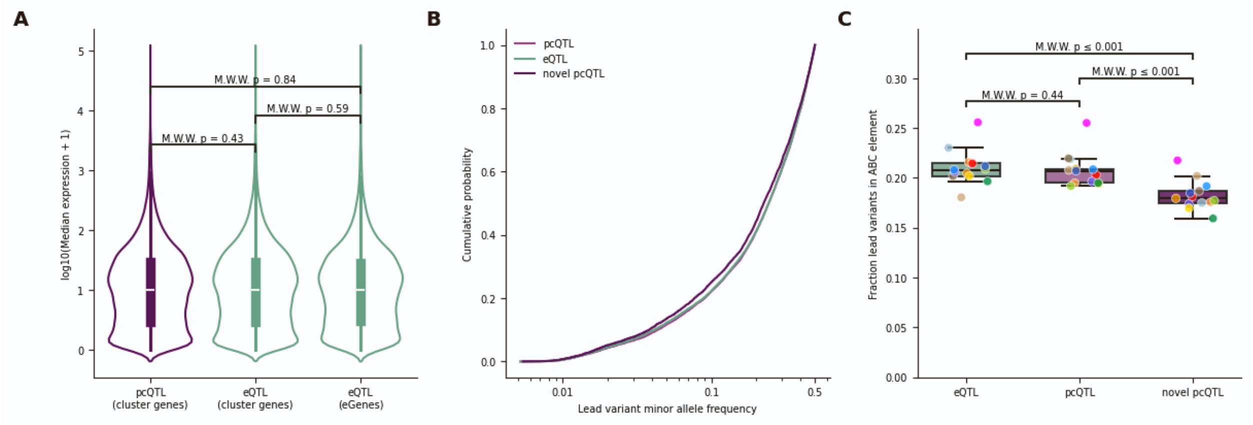

Figure S14: Properties of pcQTLs and eQTLs.

(A) In each tissue, the distribution of median expression of all genes in a cluster that had a significant pcQTL, the distribution of median expression of all genes in a cluster that had a significant eQTL, and the distribution of median expression of eGenes from clusters that had a significant eQTL; *p* values are from Mann-Whitney *U* test.

(B) Minor allele frequencies of lead variants for QTL credible sets discovered as pcQTLs or eQTLs are not significantly different ($p = 0.164$). Minor allele frequencies for novel pcQTL lead variants are lower than eQTL lead variants ($p = 1.9*10^{-4}$), and all pcQTL lead variants ($p = 1.3*10^{-6}$); *p* values are from Mann-Whitney *U* test.

(C) For each lead variant for a QTL credible set, the fraction that falls within an ABC “genic” or “intergenic” regulatory element. eQTL is all eQTL credible set lead variants, pcQTL is all pcQTL credible set lead variants, novel pcQTL is lead variants from pcQTL credible sets that did not colocalize with any eQTL credible sets; *p* values are from Mann-Whitney *U* test.

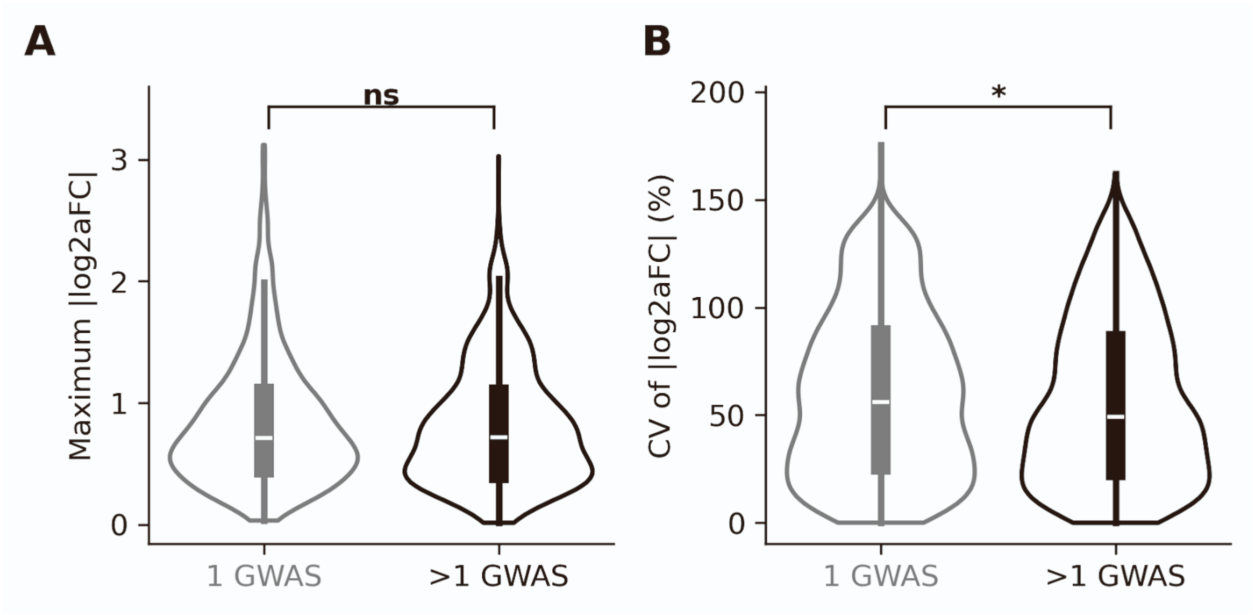

Figure S15: GWAS pleiotropy.

(A) For all credible set groups that colocalize with at least one GWAS hit, the maximum PIP-weighted $|log_2aFC|$ for any single gene in the cluster, split by whether or not the credible set group colocalizes with more than one GWAS; two-sample *t* test $p = 0.38$.

(B) For all credible set groups that colocalize with at least one GWAS hit, the CV of PIP-weighted $|log_2aFC|$ across genes in the cluster (quantifying how concentrated vs distributed the effect is) split by whether or not the credible set group colocalizes with multiple GWAS hits; two-sample *t* test $p = 0.02$.

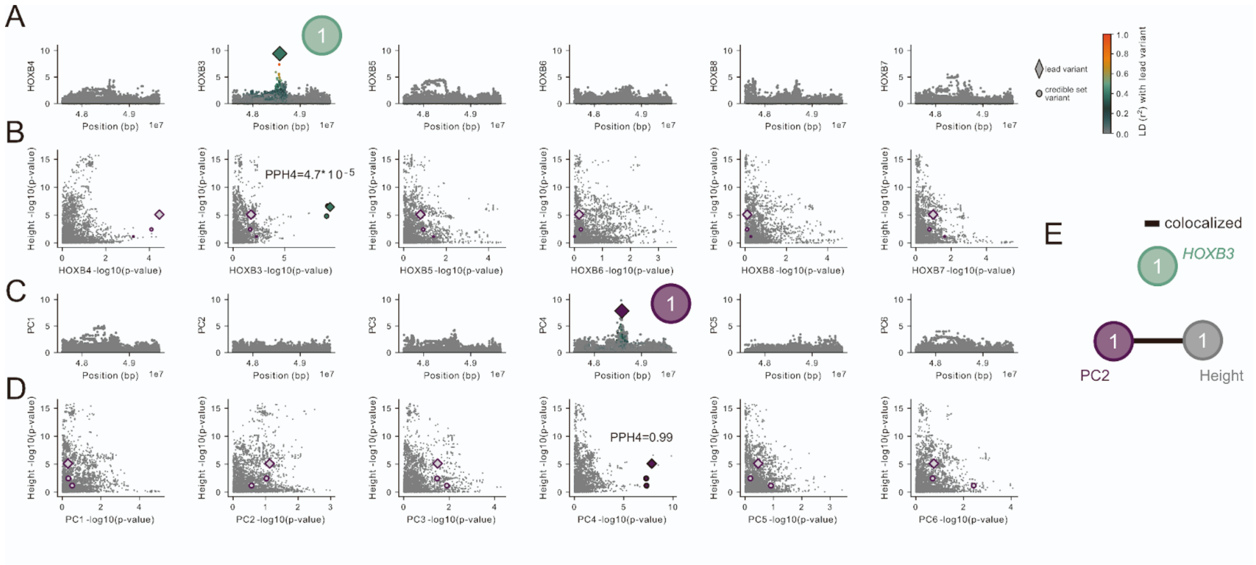

Figure S16: Height colocalization for a *HOXB* cluster eQTL and pcQTL.

(A) QTL nominal *p* values for each gene's expression vs genomic position. If the phenotype has any significant credible sets, credible set variants are highlighted and colors given by variant LD with the credible-set lead variant.

(B) GWAS variant nominal *p* values vs QTL nominal *p* values for each gene's expression. If the phenotype has any significant credible sets, credible set variants are highlighted filled diamonds/circles and the highest PP_{H4} (probability of colocalization) is given between QTL credible set and any GWAS credible sets. The novel pcQTL credible set is highlighted with outlined diamonds/circles.

(C) Same as (A), but for each PC phenotype.

(D) Same as (B), but for each PC phenotype.

(E) Colocalizations between credible sets in the cluster, colocalized is $PP_{H4} > 0.75$.

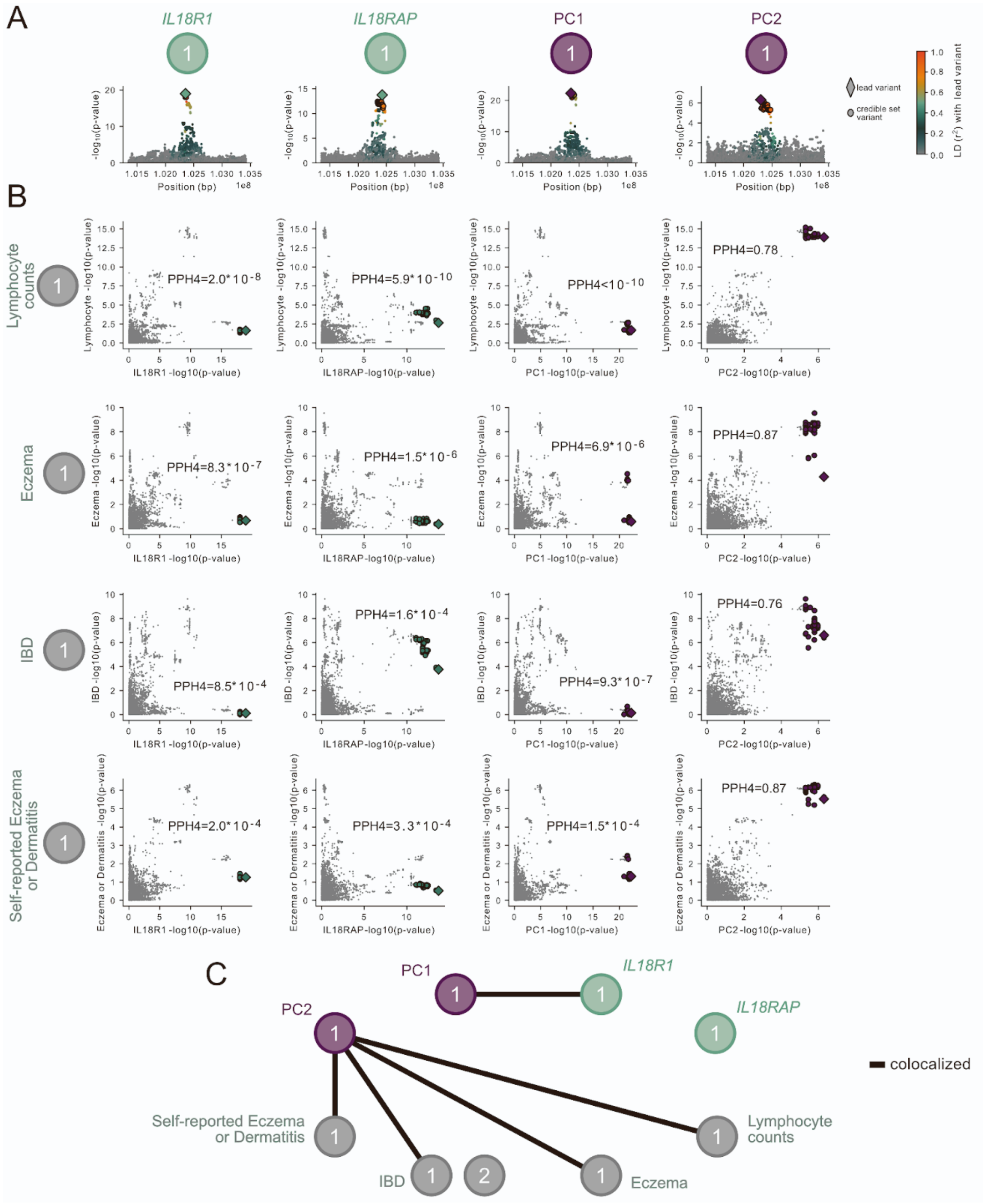

Figure S17: Lymphocyte count, eczema, IBD, and dermatitis colocalization for *IL18R1* and *IL19RAP* eQTL and pcQTL.

(A) QTL nominal *p* values for each gene's expression and each PC vs genomic position. If the phenotype has any significant credible sets, a plot is shown for each credible set, with credible set variants highlighted and colors given by variant LD with the credible-set lead variant.

(B) GWAS variant nominal p-values vs QTL nominal *p* values for each gene's expression and each PC, with credible set variants highlighted and the highest probability of colocalization (PP_{H4}) between any QTL phenotype credible set and any GWAS credible set.

(C) Colocalizations between credible sets in the cluster, colocalized is $PP_{H4} > 0.75$.

Table S1: GWAS colocalizations across tissues and traits. The total number of GWAS hits near clusters is the number of unique GWAS lead variants fine-mapped within 1MB of a gene cluster. The total number of GWAS hits near clusters per tissue is the number of unique GWAS lead variants fine-mapped within 1MB of a gene cluster in each tissue. The number of GWAS hits colocalized per GWAS trait in each tissue is given for eQTLs and for novel pcQTLs (pcQTLs not colocalized by an eQTL).
