# Supplementary material for: Focus on single-gene effects limits discovery and interpretation of complex-trait-associated variants
Source: Am J Hum Genet. 2026 Mar 23;113(4):842–51. doi: 10.1016/j.ajhg.2026.02.022 (PMC13087463; doi:10.1016/j.ajhg.2026.02.022)
Supplement: Document S2. Article plus supplemental information [file mmc3.pdf]

Focus on single-gene effects limits discovery and interpretation of complex-trait-associated variants

Graphical abstract

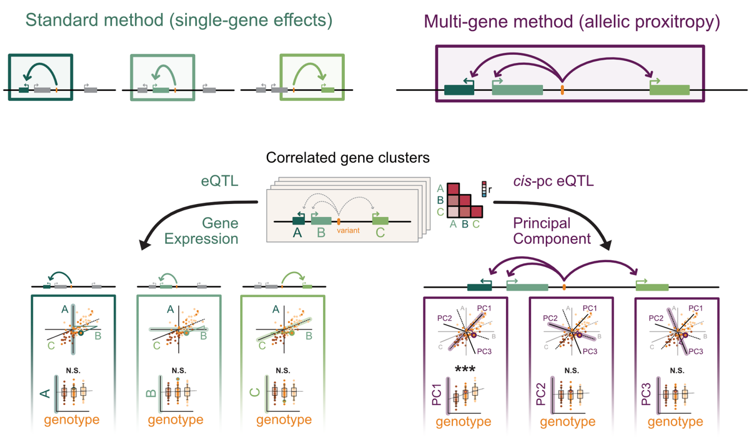

Authors

Kathryn A. Lawrence,
Tamara Gjorgjieva, Daniel Nachun,
Stephen B. Montgomery

Correspondence

klawren@stanford.edu (K.A.L.),
smontgom@stanford.edu (S.B.M.)

Standard eQTL mapping and GWAS interpretation assume that each variant acts on one gene. However, neighboring genes can exhibit "allelic proxitropy," where regulatory effects are shared. To identify these effects, we map QTLs on expression principal components of co-expressed neighboring genes. We discover QTLs and GWAS colocalizations missed by single-gene methods.

Lawrence et al., 2026, The American Journal of Human Genetics *113*, 842–851

April 2, 2026 © 2026 The Authors. Published by Elsevier Inc. on behalf of American Society of Human Genetics.

https://doi.org/10.1016/j.ajhg.2026.02.022

ARTICLE

Focus on single-gene effects limits discovery and interpretation of complex-trait-associated variants

Kathryn A. Lawrence,[1,*] Tamara Gjorgjieva,[1] Daniel Nachun,[2] and Stephen B. Montgomery[1,2,3,*]

Summary

Standard quantitative trait locus (QTL) mapping approaches consider variant effects on a single gene at a time, despite abundant evidence of allelic pleiotropy, where a single variant can affect multiple genes simultaneously. While allelic pleiotropy describes variant effects on both local and distal genes or a mixture of molecular effects on a single gene, here, we specifically investigate allelic expression "proxitropy," where a single variant influences the expression of multiple, neighboring genes. We introduce a multi-gene expression QTL (eQTL) mapping framework—*cis*-principal-component eQTL (*cis*-pc eQTL or pcQTL)—to identify variants associated with shared axes of expression variation across a cluster of neighboring genes. We perform pcQTL mapping in 13 GTEx human tissues and discover novel loci undetected by single-gene approaches. In total, we identify an average of 1,396 pcQTLs/tissue, 27% of which were not discovered by single-gene methods. These novel pcQTLs colocalized with an additional 176 genome-wide association study (GWAS) trait-associated variants and increased the number of colocalizations by 33% over single-gene QTL mapping. These findings highlight the idea that moving beyond single-gene-at-a-time approaches toward multi-gene methods can offer a more comprehensive view of gene regulation and complex-trait-associated variation.

Introduction

Co-expression of nearby genes is a widespread phenomenon. Empirically, across human tissues in GTEx, 13%–53% of genes have expression correlated with their neighbor.[1] This observed correlation can result from several biological mechanisms—for instance, transcription factors co-regulating multiple genes in *trans*, shared proximal regulatory elements such as promoters and enhancers co-regulating multiple genes in *cis*, genes sharing a local chromatin state or epigenetic marks, etc.—or technical artifacts.[2–5] Previous work has also revealed abundant allelic pleiotropy, where one variant associates with the molecular phenotypes of multiple nearby genes.[1,6] Combined, these observations indicate that a proportion of non-coding genetic effects do not act on only one causal gene.

However, current standard approaches to understanding the effects of genetic variation still focus on one-variant-one-gene mapping approaches, despite observations of co-expression and of sharing of *cis*-regulatory mechanisms among neighboring genes.[7] For instance, the widely utilized expression quantitative trait locus (eQTL) framework considers only one gene at a time, where the expression of a single gene is regressed on a genetic variant, resulting in an estimate of that genetic variant's linear effect on gene expression.[8] We hypothesize that QTL methods jointly considering neighboring genes (*cis* multi-gene QTL mapping) will improve our ability to detect and interpret the impact of common genetic variation on gene expression.

To overcome the limitations of single-gene analyses, we introduce a multi-gene QTL mapping approach—*cis*-principal-component QTLs (*cis*-pc eQTLs, or pcQTLs for brevity)—to jointly analyze clusters of neighboring, co-expressed genes across 13 human tissues from GTEx. Using this approach, we discover novel genetic effects missed by single-gene analyses. We further demonstrate improvements to colocalization with genome-wide association study (GWAS) hits, uncovering 33% additional trait-associated genetic variants missed by the traditional single-gene eQTL approach. Our results demonstrate that jointly analyzing neighboring, co-expressed genes leverages shared regulatory architecture and allelic proxitropy, improving our ability to detect and interpret genetic effects on gene expression and complex human traits.

Methods

Processing of GTEx RNA-seq data

Normalized expression data from GTEx v.8 for adipose (subcutaneous), adipose (visceral omentum), tibial artery, cultured fibroblasts, esophagus (mucosa), esophagus (muscularis), lung, skeletal muscle, tibial nerve, skin (not sun exposed), skin (sun exposed), thyroid, and whole blood were downloaded from the GTEx portal (https://storage.googleapis.com/adult-gtex/bulk-qtl/v8/single-tissue-cis-qtl/GTEx_Analysis_v8_eQTL_expression_matrices.tar).[9] Tissues were chosen based on having the largest sample sizes (number of RNA sequencing [RNA-seq] samples > 400).

Expression data were then residualized on the same covariates that were used in the standard GTEx eQTL pipeline

[1]Department of Genetics, Stanford University School of Medicine, Stanford, CA 94305, USA; [2]Department of Pathology, Stanford University School of Medicine, Stanford, CA 94305, USA; [3]Department of Biomedical Data Science, Stanford University School of Medicine, Stanford, CA 94305, USA
*Correspondence: klawren@stanford.edu (K.A.L.), smontgom@stanford.edu (S.B.M.)
https://doi.org/10.1016/j.ajhg.2026.02.022.
© 2026 The Authors. Published by Elsevier Inc. on behalf of American Society of Human Genetics.
This is an open access article under the CC BY license (http://creativecommons.org/licenses/by/4.0/).

(https://storage.googleapis.com/adult-gtex/bulk-qtl/v8/single-tissue-cis-qtl/GTEx_Analysis_v8_eQTL_covariates.tar.gz): 60 probabilistic estimation of expression residuals (PEER) factors, the top 5 genotype principal components (PCs), sequencing platform, sequencing protocol, and sex.[10]

Calling clusters of neighboring, correlated genes from RNA-seq data

To call clusters, a stepwise, iterative "sliding window" approach was used to identify stretches of neighboring, co-expressed genes ("gene clusters"). This was done for each tissue individually. Only genes with an expression level higher than the GTEx eQTL expression threshold for that tissue were considered (>0.1 transcripts per million [TPM] in at least 20% of samples and ≥6 reads in at least 20% of samples).[10]

First, for each chromosome, the Spearman's (scipy v.1.10) correlation between normalized and residualized gene expression for each pair of genes was calculated. Positive and negative correlations were both considered. We identified clusters where more than 70% of correlations between pairs of genes within the cluster were significant, such that in a cluster with n genes and m significant correlations, we require $0.7 * C(n,2) \leq m$.

Correlations were considered significant at $p < 0.05$ after Bonferroni correction for the total number of pairs of genes on the chromosome, $C(N,2)$, where N is the total number of genes on the chromosome. This is an overly conservative threshold, as in practice our maximum cluster size ensures that we do not consider pairs of genes that are more than the maximum cluster size apart. To find these clusters, we started with a maximum cluster size of $n = 50$ genes and continued until a minimum cluster size of $n = 2$ genes. Algorithmically, assuming N genes total on the chromosome $G_0, \ldots, G_N$, for each cluster size of n, we did the following:

(1) We checked whether the first n neighboring genes on the chromosome, G_0–G_n, were sufficiently correlated and did not belong to any existing clusters.
  a. If so, we recorded G_0–G_n as a cluster.
(2) Sliding window 1 along the chromosome, we checked if the next n neighboring genes on the chromosome, G_1–G_{n+1}, were sufficiently correlated and did not belong to any existing clusters.
  a. If so, we recorded G_1–G_{n+1} as a cluster.
(3) We continued with G_1–G_{n+2}, G_3–G_{n+3}, …, G_{N-n}–G_N. At each step, we moved the window of considered genes over by one and then checked whether the genes were sufficiently correlated and did not belong to any existing clusters.
  a. If so, we recorded the genes as a cluster.
(4) After reaching the end of the chromosome, we reduced the cluster size from n genes to $n - 1$ genes and then restarted at step 1. We repeated this process until the minimum window size ($n = 2$) was reached.

This approach captured genes in the largest possible cluster with significant within-cluster correlations. As false positive correlations due to cross-mappable reads were a concern, we compared the number of QTLs mapped for clusters with and without any genes containing cross-mappable 75-mers (Figure S1).[11] As the QTL discovery rate was not statistically different between clusters with and without cross-mappable and non-cross-mappable genes, we continued to consider cross-mappable clusters for further analysis.

Cluster enrichments for annotations

In order to evaluate whether clusters were enriched for particular annotations (such as "paralogs" or "shared GO terms"), we calculated cluster enrichments for all correlated clusters of 2, 3, 4, or 5 genes against a background of null clusters. Null clusters were all sets of 2, 3, 4, or 5 neighboring genes in each tissue that were not part of a correlated cluster in that tissue, sampled at a rate to match the relative distributions of 2, 3, 4, or 5 gene clusters in the correlated cluster set. Correlated and null clusters were assigned a boolean label: 0 if the cluster did not belong to an annotation category or 1 if the cluster did belong to an annotation category. Annotation categories are not mutually exclusive. These labels were used as the dependent variable in logistic regression to calculate enrichment odds ratios. The number of genes in the cluster and the log length of the cluster (defined by the outer edges of any transcript in the cluster in bp) were included as covariates. If the expected frequency in any cell of the contingency table was less than 5, enrichment calculations were skipped.

For each correlated and null cluster, the cluster was assigned a label of 1 for the given annotation if it fulfilled the following requirements:

(1) Shared opposite-strand promoter (bidirectional promoter): if, for any pair of genes A/B in the cluster, the 5′ end of any annotated GENCODE v.26 transcript for gene A was within 1,000 bp of the 5′ end of any annotated transcript for gene B and the genes in the pair were on opposite strands.[12]
(2) Shared same-strand promoter: if, for any pair of genes A/B in the cluster, the 5′ end of any annotated GENCODE v.26 transcript for gene A was within 1,000 bp of the 5′ end of any annotated transcript for gene B and the genes in the pair were on the same strand.[12]
(3) Overlapping opposite strand: if, for any pair of genes in the cluster, the genes overlapped, were on opposite strands, and did not share a promoter.
(4) Overlapping same strand: if, for any pair of genes in the cluster, the genes overlapped, were on the same strand, and did not share a promoter.
(5) Enhancer: if any pair of genes in the cluster shared a "genic" or an "intergenic" class enhancer with a score of >0.1 in ABC enhancer-gene predictions, for any cell type.[13] ABC enhancer-gene connections were downloaded from the Engriez lab website (https://mitra.stanford.edu/engreitz/oak/public/Nasser2021/AllPredictions.AvgHiC.ABC0.015.minus150.ForABCPaperV3.txt.gz) and converted to hg38 with liftOver (default settings).[14]
(6) Paralogs: if any pair of genes in the cluster were listed as paralogs. Paralog information was obtained from the BioMart webtool for Ensembl 97 (https://www.ensembl.org/info/data/biomart/index.html).[15]
(7) Shared Gene Ontology (GO) term: if any pair of genes in the cluster shared a biological process (BP) GO term. GO terms for each gene were obtained from the BioMart webtool for Ensembl 97 (https://www.ensembl.org/info/data/biomart/index.html).[15]
(8) Cross CCCTC-binding factor (CTCF) peak: if a CTCF peak fell within the window defined by the outer edges of any

transcript in the cluster. ENTEx CTCF chromatin immunoprecipitation (ChIP)-seq peaks from tissue-matched GTEx samples were used (https://www.encodeproject.org/entex-matrix/?type=Experiment&status=released&internal_tags=ENTEx).[16] Specific file IDs are given on GitHub https://github.com/kal26/pcqtls/blob/main/references/ctcf_matched_gtex.txt).

(9) Cross transcriptionally associated domain (TAD) boundary: if the edge of a TAD fell within the window defined by the outer edges of any transcript in the cluster. TAD boundaries calculated from Hi-C data in GM12878 with a directionality index (DI) at a 10 kb resolution were downloaded from TADKB (http://dna.cs.miami.edu/TADKB/)[17] and converted to hg38 with liftOver (default settings).[14]

Cluster PCs

For each cluster of genes, principal-component analysis (PCA) (sklearn v.1.3.2, default settings) was used to find shared axes of expression variance. PCs were constructed as a linear combination of the normalized, residualized expression of the genes in the cluster.

PC-normalized shared variance

To find the shared variance explained by the primary PC (PC1), given a cluster of *n* genes, we first summed the variance explained for each gene in the cluster by PC1:

$$\sum_{i=1}^{n} \hat{l}_i^{\,2},$$

where the variance explained by PC1 for gene *i* is the squared loading $\hat{l}_i^{\,2}$ for PC1 onto that gene. We then subtracted 1 and normalized to cluster size so that shared variance was

$$\frac{\sum_{i=1}^{n} \hat{l}_i^{\,2} - 1}{n - 1} * 100.$$

This rescales the value so that regardless of cluster size, if the expression of all genes is uncorrelated, the normalized shared variance is 0%, and if the expression of all genes is perfectly correlated, the normalized shared variance is 100%.

Discovery and fine-mapping of pcQTLs and eQTLs

SuSiE (susieR v.0.12.35)[18] (https://stephenslab.github.io/susieR/) was used for discovery and fine-mapping of independent loci and their 95% credible sets of variants. SuSiE was run with default parameters (default uniform prior across variants, L = 10, purity filter min_abs_corr = 0.5) and applied to individual-level genotype and expression or PC data. Only 3 single-gene expression phenotypes and 1 PC phenotype yielded the maximum of 10 credible sets, indicating that setting L = 10 was sufficient to capture the number of independent loci underlying our single-gene expression and PC phenotypes (Figure S2).

In each tissue independently, SuSiE was run on the following input phenotypes:

(1) eQTL mapping: the expression of each gene in each cluster.
(2) pcQTL mapping: each of the PCs derived from the expression of the genes in the cluster.

SuSiE was run on each PC and expression phenotype as defined above for all clusters, without pre-filtering for single-gene expression and PC phenotype significance to avoid possible thresholding effects that a false discovery rate (FDR) filter could introduce.

For both eQTL and pcQTL mapping, we considered all SNPs with a minor-allele frequency (MAF) of 0.05 or more within the variant window defined by ±1 Mb around the union (min start to max end) of the collapsed gene annotation locations of all genes in the cluster. This ensured that both eQTL and pcQTL mappings were applied to the same set of variants. Variant call files of genotype data were obtained from dbGaP (dbGaP: phs000424.v8) based on the GRCh38/hg38 reference.

Fine-mapping calibration

To assess the calibration of pcQTL and eQTL fine-mapping, we performed a genotype-shuffling null analysis in fibroblasts. We analyzed phenotypes derived from 861 gene clusters (1,872 PC-based cluster expression phenotypes and 1,872 single-gene expression phenotypes). For each phenotype, we generated a null dataset by randomly permuting the individual-to-genotype matching while keeping phenotypes fixed, removing true genotype-phenotype associations but preserving genotype correlation structure and phenotype distributions. We then ran SuSiE fine-mapping on the shuffled-null data using the same workflow and parameters as in the real-genotype analyses (Figure S3).

Phenotype-level permutation-based FDR

TensorQTL (https://github.com/broadinstitute/tensorqtl)[19] was run with default settings in *cis* mode to generate permutation-based empirical *p* values and *q* values, enabling phenotype-level FDR estimation. We calculated phenotype-level FDR for three conditions.

(1) Single-gene eQTLs individually.
(2) pcQTLs individually.
(3) Both single-gene eQTLs and pcQTLs together.

Significant single-gene expression and PC phenotypes at a 5% FDR are those with $q < 0.05$.

QTL-to-transcription start site distance

Closest gene (cluster) distance was calculated as the minimum distance from any lead variant in the credible set group to the transcription start site (TSS) of any cluster gene transcript. Note that for eQTLs, this may not be the eGene on which the eQTL was mapped. The closest gene (eGene) distance was calculated as the minimum distance from any lead variant in the credible set group to the TSS of any cluster gene that was an eGene for an eQTL credible set in the group. For genes on the positive strand, the distance was calculated as variant position − TSS, and for genes on the negative strand, the distance was calculated as TSS − variant position. The TSS of a transcript was the start, if the transcript was on the positive strand, or the end, if the transcript was on the negative strand. All transcripts associated with a gene were used.

Posterior inclusion probability-weighted $\log_2$aFC

TensorQTL (https://github.com/broadinstitute/tensorqtl)[19] was run with default settings in *cis_nominal* mode to get summary statistics (stats) for all variant-phenotype pairs. These summary stats were used to calculate the log2 allelic fold change ($\log_2$(aFC)). To

investigate the extent to which a given pcQTL or eQTL credible set influenced each gene within a target cluster individually, we calculated the posterior inclusion probability (PIP)-weighted $\log_2$(aFC).[20]

An estimate of the effect of a given variant i on an individual gene A is given by the $log_2\left(aFC_i^A\right)$ for that variant. To estimate the effect of a credible set of j variants, $i = 1, \ldots, j$, on gene A, we multiplied the $log_2\left(aFC_i^A\right)$ for each variant in the credible set by the PIP probability PIP_i, then summed those PIP-weighted effects and divided by the sum of the PIP probabilities in the credible set:

$$\log_2\left(\mathrm{aFC}^A\right) = \frac{\sum_{i=1}^{j} PIP_i * log_2\left(\mathrm{aFC}_i^A\right)}{\sum_{i=1}^{j} PIP_i}.$$

To get the marginal effect that a credible set discovered as a pcQTL had on the expression of gene A, we used the credible set of variants and PIP probabilities from the pcQTL fine-mapping and the$\log_2$(aFC) for gene A. To get the marginal effect that a credible set discovered as an eQTL had on the expression of gene A, we used the credible set of variants and PIP probabilities from the eQTL fine-mapping and the $\log_2$(aFC) for gene A. This allowed us to compare the effects of pcQTL credible sets and eQTL credible sets on gene expression for each gene in a cluster.

To compare regulatory effect magnitudes for credible sets, we used maximum absolute values of $|\log_2(\mathrm{aFC})|$ across all genes in a cluster.

To quantify how concentrated or distributed the regulatory effects were across the gene cluster, we calculated the coefficient of variation (CV) of absolute $|\log_2(\mathrm{aFC})|$ values. A CV of 0 indicates perfectly equal effects, while higher values indicate increasingly concentrated effects on fewer genes.

PIP-weighted variant annotations

Ensembl Variant Effect Predictor (Ensembl VEP v.114)[21] was used to annotate each variant with a likely function. Rather than assigning a boolean label to each credible set for each annotation, we calculated an annotation probability for each credible set and annotation. The credible set annotation probability was a PIP-weighed sum over the boolean indicator for each variant in each annotation. These probabilities were used as the dependent variable in logistic regression to calculate enrichment odds ratios.

The candidate *cis*-regulatory elements (cCREs) used for credible set overlap were in matched EN-TEx tissues (https://downloads.wenglab.org/cCRE_decoration.matrix.1.gz).[22]

Colocalization of pcQTL, eQTL, and GWAS hits

We used coloc.SuSiE (v.5)[18,23] to colocalize pcQTL, eQTL, and GWAS signals. GWAS summary stats from a publicly available resource of 114 GWASs (for 74 distinct traits, including cardiometabolic, hematologic, neuropsychiatric, and anthropometric features from the UKBB and GIANT) harmonized and imputed to GTEx variants were used, summary stats are available on Zenodo (Zenodo: 3629742).[24] Linkage disequilibrium (LD) reference matrices for GWAS summary data were constructed using GTEx samples with PLINK (v.1.90b7.7, default settings).[25]

In each tissue independently, for each cluster, all eQTL, pcQTL, and GWAS credible sets were colocalized with all other eQTL, pcQTL, and GWAS credible sets. We considered a GWAS hit linked to at least one single-gene eQTL in a given tissue if the GWAS hit had a posterior probability of colocalization greater than 0.75 ($PP_{H4} \geq 0.75$) for its colocalization with any single-gene eQTL. We considered a GWAS hit colocalized with a pcQTL but not with any single-gene eQTL in a given tissue if, for at least one pcQTL, $PP_{H4} \geq 0.75$ but for all single-gene eQTL, $PP_{H4} < 0.75$. To calculate the number of unique GWAS hits across tissues colocalized by eQTLs or by pcQTLs, we collapsed hits by GWAS variant-trait pairs. Specifically, if the same GWAS variant for the same trait colocalized with eQTLs or by pcQTL in multiple tissues, it was counted only once. However, if the same variant was associated with multiple GWAS traits, each variant-trait pair was counted separately.

Creation of credible set groups

A custom script (https://github.com/kal26/pcqtls/blob/60f0d295d11b58bdf4035b4bbf8493828d09dd7f/workflow/scripts/group_signals.py) was used to create credible set groups. In each tissue independently, an undirected graph was constructed with each credible set as a node and an edge connecting two nodes if the probability of colocalization between those credible sets was greater than 0.75 ($PP_{H4} \geq 0.75$). Credible set groups were the set of connected components of the graph. Credible set groups were classified as “pcQTL only” if they contained only pcQTL credible sets, “eQTL only” if they contained only eQTL credible sets, or “both” if they contained both eQTL and pcQTL credible sets. “Both” credible set groups can contain eQTLs for one or more genes in the cluster.

Results

Transcriptome-wide identification of clusters of co-expressed neighboring genes

We started by identifying clusters of co-expressed neighboring genes (gene clusters) across human tissues using GTEx data. We focused on 13 tissues with large sample sizes ($N > 400$), as these are best powered for eQTL analyses. As gene expression data often have global structured variance due to technical and known biological covariates, latent factor correction using tools such as surrogate variable analysis,[26] global PCA, or PEER[27] are often employed to improve power to discover *cis*-regulatory effects.[8] We found that residualization of latent factors (60 PEER factors) also improved detection of co-expression of neighboring genes in GTEx data. While the un-residualized data showed frequent long-range correlations (median gene-gene distance of 40 Mb), after residualization, significant correlations are at the smaller scale of shared *cis*-regulation (median gene-gene distance of 290 kb) (Figure S4). This suggests that latent factor correction, by removing long-range interactions driven by global technical and biological covariates, increases the detection power for co-expression at a localized scale, thereby highlighting significant correlations driven by shared *cis*-regulatory mechanisms.

We performed a transcriptome-wide analysis to identify clusters of nearby correlated genes; we did this for each tissue independently (methods). This resulted in 787–1,138 clusters across tissues—a total of 12,022 clusters—with

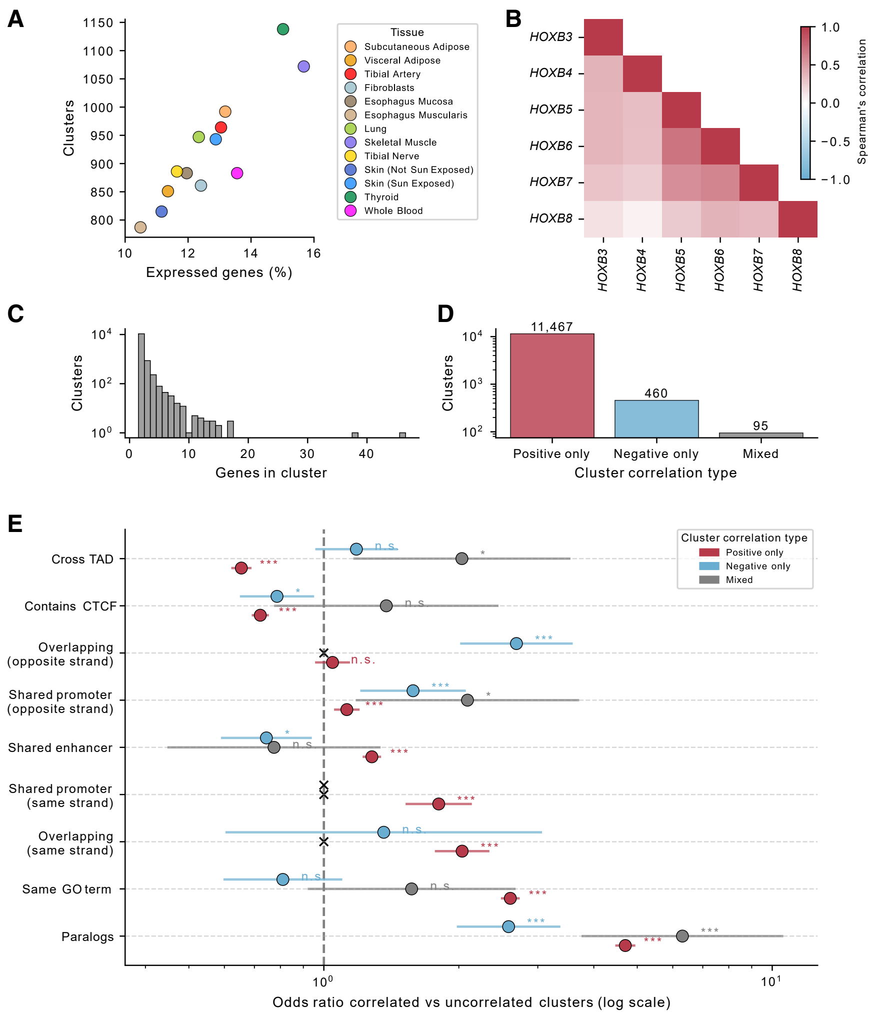

Figure 1. Discovery of correlated neighboring gene clusters
(A) The number of clusters in each tissue vs. the percentage of all expressed genes in the tissue that are in a cluster.
(B) Example heatmap of a cluster of six *HOXB* genes with correlated expression in esophagus muscularis, colored by Spearman's correlation coefficient.
(C) Distribution of the number of genes per cluster.
(D) Number of clusters with genes that are only positively correlated to one another, only negatively correlated, or have a mix of positive and negative correlation.
(E) Enrichment of correlated clusters vs. uncorrelated null clusters (methods), split for clusters with all positive correlation, all negative correlation, or a mix of positive and negative correlation. Categories where the expected frequency was less than 5 were skipped. Error bars represent 95% confidence intervals on odds ratios for logistic regression (X: skipped, ns $p > 0.05$, *$0.05 > p > 10^{-2}$, **$10^{-2} > p > 10^{-3}$, and ***$p < 10^{-3}$).

between 10.5% and 15.7% of expressed genes in each tissue belonging to a cluster (Figures 1A, S5, and S6). The majority of clusters (89.2%) were pairs, 7.2% had 3 genes, and 3.6% had 4 or more genes (Figure 1C). We observed that genes are more frequently positively correlated than negatively correlated, although 4.6% of all clusters across tissues ($n = 555$) contained at least one pair of genes with a significant negative correlation (Figure 1D). Large clusters often represent known functionally related groups of genes, such as the Hox cluster of 6 genes on chromosome 17 or the Keratin gene cluster (with 38 genes) on chromosome 17 (Figures 1B and S6C). We further found that positively correlated gene clusters are enriched for similar biological functions and regulatory architecture. They are more likely to include paralogs, belong to the same GO term, and have shared enhancers and shared promoters (both bidirectional promoters and same-strand promoters), but they are less likely to cross a TAD boundary or contain a CTCF site. Negatively correlated clusters are depleted for shared enhancers and enriched for pairs of genes with opposite strands that overlap or share a bidirectional promoter (Figure 1E).

A multi-gene QTL framework leverages common variation to detect shared genetic effects

To allow us to jointly consider gene clusters in QTL analysis, we first calculated cluster PCs from the normalized gene expression data for each gene cluster (methods). As expected for PCA on pre-selected correlated variables, variables have some proportion of shared variance. To summarize the degree to which gene clusters' expression variance is shared, we calculated the average variance explained across genes in each cluster by PC1, normalized by cluster size such that a cluster with completely correlated gene expression for all genes would have a shared variance of 100% and a cluster with completely uncorrelated expression would have a shared variance of 0%. Clusters have significantly higher ($p < 10^{-10}$) shared variance (36.6%) than non-correlated neighbor gene pairs (7.6%) (Figures 2A and S7). We then use PCs as the dependent variable in QTL mapping (pcQTL). This pcQTL framework allows us to estimate the effect of genetic variants on a shared axis of expression variance across the gene cluster.

We mapped and fine-mapped pcQTLs with SuSiE, using all PCs from each gene cluster as input phenotypes (PC1, PC2, …, PC(n) for an n-gene cluster). Across tissues, we identified 920–1,997 pcQTL credible sets per tissue (Figures 2B, S2, and S8). For comparison, we ran SuSiE analogously using the expression of each gene within a cluster as the phenotype. A permutation-based, phenotype-level FDR analysis confirmed that any PC or single-gene

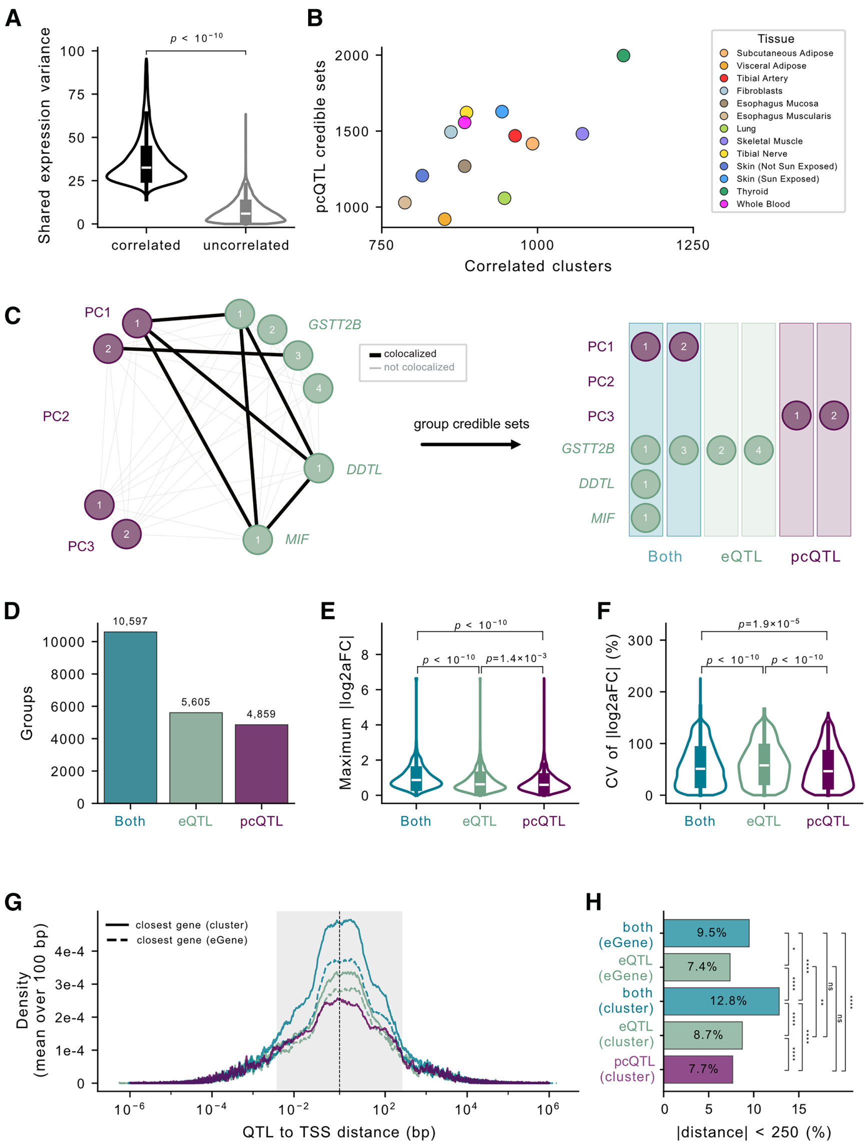

Figure 2. Novel pcQTL discovery

(A) Normalized shared variance (methods) explained by PC1 for correlated clusters vs. neighboring genes not called as clusters in fibroblasts; the p value is from a two-sample t test.
(B) Number of pcQTL credible sets vs. number of gene clusters across tissues.
(C) Example of credible set groups from a three-gene cluster. Nodes are credible sets, and edges are colored according to colocalization between the credible sets.
(D) Number of credible set groups of each type across all clusters.
(E) The maximum PIP-weighted $|\log_2(\text{aFC})|$ for any single gene in the cluster; p values are from a two-sample t test.
(F) The CV of PIP-weighted $|\log_2(\text{aFC})|$ across genes in the cluster (quantifying how concentrated vs. distributed the effect is); p values are from a two-sample t test.
(G) For each credible set group, the minimum credible set lead variant-TSS distance to any gene in the cluster, and for credible set groups containing an eQTL credible set, the minimum credible set lead variant-TSS distance to any eGene in the cluster (gene with a significant eQTL credible set in the group). The gray shaded area is $|\text{distance}| < 250$ bp. Lines are colored by credible set group type.
(H) Proportion of lead variants with $|\text{distance}| < 250$ bp for credible set group-type and distance-type categories in (G). $p$values are from Fisher's exact test (ns $p > 0.05$, *$0.05 > p > 10^{-2}$, **$10^{-2} > p > 10^{-3}$, and ***$p < 10^{-3}$).

expression phenotype with one or more mapped credible sets was significant at a 5% FDR (Figure S9).

In order to identify how many of the pcQTLs are novel, we colocalized each pcQTL credible set to every eQTL credible set for each cluster (Figure 2C). We found that 98% of credible set groups with two or more eGenes were also tagged by a pcQTL (Figures S10 and S11). pcQTL credible sets that did not colocalize to any eQTL credible sets for any gene in the cluster were considered novel. Across tissues, we found 4,859 novel pcQTLs (26.7% of all pcQTL credible sets) that were not discovered by any single-gene eQTL analysis (Figures 2D and S8).

To investigate why the pcQTL approach identifies novel signals, we calculated the $\log_2(\text{aFC})$ effect of each pcQTL credible set on the expression of each gene within its cluster (methods). This gives us an estimate of the effect of a given QTL on the expression of each gene individually. Compared to eQTLs, we found that novel pcQTLs from PC1 signals had a smaller effect on any single gene in their cluster ($p = 1.4 \times 10^{-3}$) (Figure 2E) but that effects were more distributed across genes ($p < 10^{-10}$) (Figures 2F and S12), indicating that, as expected, PC1s summarized smaller-size effects distributed across all genes in their correlated cluster.

Multi-gene pcQTLs colocalize with new GWAS hits

Most trait-associated variants in GWASs lie in non-coding regions and are thought to affect traits by regulating gene expression, yet identified eQTLs and sQTLs only colocalize with a small percentage of GWAS hits (47% linked to target genes in GTEx across all tissues).[24] We sought to investigate whether our newly discovered pcQTLs can help explain additional GWAS hits. To do this, we colocalized our pcQTLs with GWAS hits for 74 traits, including cardiometabolic, hematologic, neuropsychiatric, and anthropometric features from the UKBB and GIANT.[24] For comparison, we also colocalized GWAS hits and eQTLs for each gene in each cluster. Using single-gene eQTL mapping alone, 2,033 GWAS hits could be linked to at least one single-gene eQTL in a cluster in a given tissue (representing 792 unique

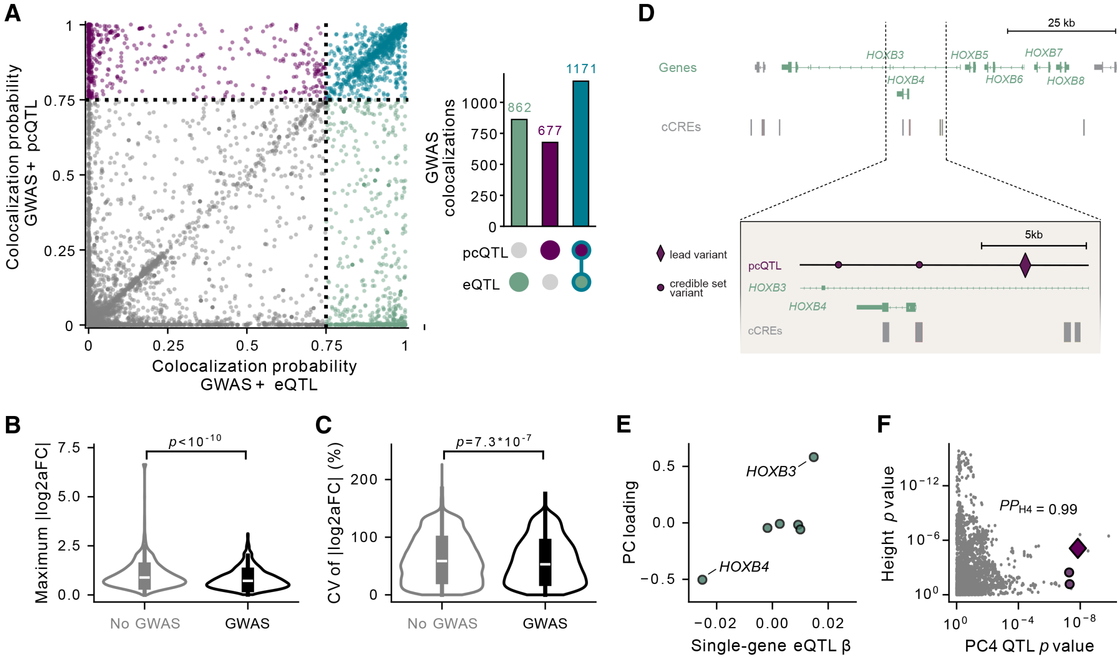

Figure 3. pcQTL colocalization with complex-trait-associated variants

(A) Maximum posterior probability of a shared causal variant (PP_{H4}) underlying the GWAS hit and any pcQTL credible set for the cluster vs. for any eQTL credible set in the cluster. $PP_{H4} = 0.75$ is shown as a dotted line. UpSet plot (right): total GWAS colocalizations across all clusters and tissues.

(B) For all credible set groups, the maximum PIP-weighted $|\log_2(\text{aFC})|$ for any single gene in the cluster, split by whether or not the credible set group colocalizes with a GWAS hit; the p value is from a two-sample t test.

(C) For all credible set groups, the CV of PIP-weighted $|\log_2(\text{aFC})|$ across genes in the cluster (quantifying how concentrated vs. distributed the effect is), split by whether or not the credible set group colocalizes with a GWAS hit; the p value is from a two-sample t test.

(D) *HOXB3*, *HOXB4*, *HOXB5*, *HOXB6*, *HOXB7*, and *HOXB8* gene cluster and cCRE regulatory elements in esophagus muscularis; close-up of region with fine-mapped novel PC4 pcQTL credible set.

(E) Marginal effect of pcQTL on each eGene vs. PC4 loading onto each eGene.

(F) Nominal p values for height GWAS and PC4. Posterior probability of colocalization between the GWAS hit and the pcQTL credible set PP_{H4} is 0.99.

GWAS hits across tissues). With multi-gene mapping on cluster PCs, an additional 677 GWAS hits were colocalized with a pcQTL but not with any single-gene eQTLs with a cluster in a given tissue (representing 176 unique GWAS hits) (Figures 3A and S13; Table S1). This represents a 33% increase in colocalizations (22% increase in unique GWAS hits) compared with single-gene eQTLs alone.

Consistent with evidence that large-effect QTL variants are less likely to colocalize with GWAS hits because stronger regulatory effects at crucial genes tend to be purged by negative selection,[28] we found that larger-effect QTLs in our analysis were less likely to colocalize with GWAS hits ($p < 10^{-10}$) (Figure 3B). Previous work has demonstrated that GWAS hits tend to have lower allele frequencies and relative depletion near gene TSSs relative to single-gene eQTLs[13]; pcQTLs also share these properties (Figures 2G, 2H, and S14). QTLs with smaller effects on any single gene but with effects distributed across multiple genes (quantified by lower CV $|\log_2(\text{aFC})|$; methods) showed higher rates of colocalization ($p = 7.3 \times 10^{-7}$; Figure 3C) and were more likely to be pleiotropic ($p = 0.023$; Figure S15). These weak, multi-gene effects are precisely the type of signal that pcQTL mapping can capture more effectively.

An example of one such novel pcQTL credible set colocalization illustrates how PCs can boost power by summarizing distributed effects across genes. The colocalization is between PC4 for a 6-gene *HOXB* cluster (*HOXB3*, *HOXB4*, *HOXB5*, *HOXB6*, *HOXB7*, and *HOXB8*) in esophagus muscularis tissue and a GWAS hit for height (Figure 3D). Although the *HOXB* transcription factors play a well-established role in development[29] and single-gene eQTL analysis maps an eQTL for *HOXB3*, this single-gene *HOXB3* eQTL did not colocalize with the height GWAS hit ($PP_{H4} = 4.7 \times 10^{-5}$). When we instead used PC4 for pcQTL mapping, we detected a novel locus that colocalized with the GWAS hit ($PP_{H4} = 0.99$) (Figure 3F).

Based on PC loadings, PC4 primarily captured inverse variation between *HOXB3* and *HOXB4* (Figure 3E). The two genes are positively correlated (Figure 1B), but this positive correlation is summarized in the first 3 PCs, allowing PC4 to capture a subtler inverse effect. Fine-mapping established a three-variant credible set for the pcQTL (Figure 3D), and while the credible set variants have below-threshold nominal p values for some genes

individually, none reach significance on their own (Figure S16). One variant of the credible set overlaps a cCRE region in esophagus muscularis (Figure 3D), and notably, enhancer-promoter links from the ABC model indicate that the regulatory element acts as both the promoter of *HOXB4* and an enhancer for *HOXB3*, the same two genes that load onto PC4. Multi-gene pcQTL analysis can reveal this kind of joint regulatory effect, which would be missed by single-gene analysis, providing potential insights into loci that manifest as coordinated changes in gene expression relationships rather than strong individual gene effects.

A second novel pcQTL colocalization further demonstrates how pcQTL analysis can reveal loci missed by single-gene eQTLs. A pcQTL from PC2 for a two-gene cluster of interleukin 18 (IL-18) receptor complex genes (*IL18RAP* and *IL18R1*) in sun-exposed skin tissue colocalizes with a pleiotropic GWAS hit for eczema ($PP_{H4} = 0.87$), dermatitis ($PP_{H4} = 0.87$), lymphocyte count ($PP_{H4} = 0.78$), and inflammatory bowel disease (IBD) ($PP_{H4} = 0.76$) (Figure S17). The IL-18 cytokine is a pro-inflammatory factor, previously implicated in a wide range of inflammatory skin disorders, with elevated IL-18 levels observed in patients with eczema, dermatitis, or IBD.[30–32] As in the previous example, single-gene analysis maps individual eQTLs, one for *IL18RAP* and one for *IL18R1*, but neither colocalizes with the inflammatory disease GWAS signals (max $PP_{H4} = 7.6 \times 10^{-4}$; Figure S17).

The novel pcQTL fine-maps to an 87-variant credible set in strong LD (LD r^2: 0.86–1.0), with variants that overlap the *IL18RAP* promoter and five ABC enhancer elements linked to both *IL18R1* and *IL18RAP*. The complex-trait associations may result from only a single causal variant or from multiple causal variants on the haplotype,[33] but only by considering the variants' combined effect on both genes are we able to map the pcQTLs. These examples highlight how complex-trait associations can emerge from coordinated effects on co-regulated genes—effects that would be overlooked when genes are analyzed in isolation but become detectable through multi-gene approaches such as pcQTLs.

Discussion

eQTL studies have focused on single-gene-at-a-time analyses despite evidence of molecular pleiotropy, where variants impact the molecular function of multiple genes. To detect such pleiotropic variants, previous work has primarily focused on mapping single-gene eQTLs and reporting instances where a variant is independently associated with multiple genes rather than leveraging shared effects.[1,6] We demonstrate that when we move from considering genes as discrete functional units to jointly considering multiple, neighboring genes, we detect novel QTLs and new colocalizations with complex-trait-associated variation.

Because multi-gene regulatory effects can operate through diverse causal mechanisms—affecting one gene or multiple genes simultaneously or creating gene-gene interactions—an additional layer of complexity exists for the interpretation of pcQTLs. These complexities can be hidden when analyzing single-gene eQTLs when each gene is considered in isolation. We expect that using a *cis*-multi-gene approach will clarify the existence of more complex mechanisms, presenting opportunities for expanding experimental validation and functional follow-up approaches when studying genetic risk factors.

In our study, pcQTLs represent a proof-of-concept methodology for *cis*-multi-gene eQTL mapping to uncover shared local gene regulation. This approach builds on a wide range of previous work where grouping of related phenotypes has been a versatile approach to uncover novel signals, such as the grouping of GWAS traits to leverage phenotypic pleiotropy,[34,35] the grouping of spatially correlated epigenomic signals,[36,37] or the grouping of co-expressed genes genome wide to capture *trans*-network and pathway effects.[38–41] By focusing specifically on neighboring genes, pcQTLs enrich for shared *cis*-regulation. Further work will be required to determine which clustering methods and statistical tools best capture shared *cis*-regulation, and different aspects of multi-gene QTL discovery may require different strategies. However, the dramatic increase in discoveries and complex-trait-associated variation even with a methodologically straightforward approach such as pcQTLs highlights that leveraging shared *cis*-regulation and subsequent co-expression of neighboring genes is an important aspect of QTL mapping that can elucidate new trait and disease biology.

Combined, this approach recognizes the complexity of gene regulation, leveraging the expression correlations among nearby genes to capture novel signals of molecular pleiotropy. However, as molecular pleiotropy can describe both local and distal effects, we classify variants with effects on multiple, proximal genes as having molecular proxitropy. We expect that future studies will benefit from moving from a classical genetics view of a causal variant impacting a single causal gene to one integrating the complexity of local regulation.

Data and code availability

The accession number for the correlated gene clusters, PC and expression inputs for QTL mapping, eQTL and pcQTL summary stats, eQTL and pcQTL SuSiE credible sets, QTL-QTL and QTL-GWAS colocalizations, and credible set groups reported in this paper is [Zenodo]: 18320563.

Scripts used to call clusters, pipelines for data processing, links to all annotation files, and notebooks to generate all figures are available at https://github.com/kal26/pcqtls.

Acknowledgments

We thank the donors and their families for their generous gifts of biospecimens to the GTEx research project. The GTEx project was

supported by the Common Fund of the Office of the Director of the National Institutes of Health (NIH) (http://commonfund.nih.gov/GTEx). Additional funds were provided by the National Cancer Institute (NCI); the National Human Genome Research Institute (NHGRI); the National Heart, Lung, and Blood Institute (NHLBI); the National Institute on Drug Abuse (NIDA); the National Institute of Mental Health (NIMH); and the National Institute of Neurological Disorders and Stroke (NINDS). This research was supported by NIH grants R01MH12524, U01AG072573, and U01HG012069 to S.B.M. K.A.L. is supported by the Stanford Genome Training Program (SGTP; NIH/NHGRI T32HG000044). T.G. is supported by the Knight-Hennessy Scholars fellowship. The funders had no role in the study design, data collection and analysis, decision to publish, or preparation of the manuscript.

Author contributions

S.B.M., T.G., D.N., and K.A.L conceived and designed the study. T.G. designed the cluster-calling algorithm. K.A.L. performed the remainder of the analyses. S.B.M., T.G., D.N., and K.A.L. wrote the manuscript.

Declaration of interests

S.B.M. is on the scientific advisory board of MyOme and PhiTech.

Declaration of generative AI and AI-assisted technologies in the writing process

During the preparation of this work, the authors used OpenAI-o4 and Claude-4 to improve language clarity and figure aesthetics. After using this tool/service, the authors reviewed and edited the content as needed and take full responsibility for the content of the publication.

Supplemental information

Supplemental information can be found online at https://doi.org/10.1016/j.ajhg.2026.02.022.

Web resources

Zenodo, https://zenodo.org/records; dbGaP, https://dbgap.ncbi.nlm.nih.gov/home/

Received: June 20, 2025
Accepted: February 26, 2026
Published: March 23, 2026

References

1. Ribeiro, D.M., Rubinacci, S., Ramisch, A., Hofmeister, R.J., Dermitzakis, E.T., and Delaneau, O. (2021). The molecular basis, genetic control and pleiotropic effects of local gene coexpression. Nat. Commun. *12*, 4842. https://doi.org/10.1038/s41467-021-25129-x.
2. Delaneau, O., Zazhytska, M., Borel, C., Giannuzzi, G., Rey, G., Howald, C., Kumar, S., Ongen, H., Popadin, K., Marbach, D., et al. (2019). Chromatin three-dimensional interactions mediate genetic effects on gene expression. Science *364*, eaat8266. https://doi.org/10.1126/science.aat8266.
3. Long, H.S., Greenaway, S., Powell, G., Mallon, A.-M., Lindgren, C.M., and Simon, M.M. (2022). Making sense of the linear genome, gene function and TADs. Epigenetics Chromatin *15*, 4. https://doi.org/10.1186/s13072-022-00436-9.
4. Duggal, G., Wang, H., and Kingsford, C. (2014). Higher-order chromatin domains link eQTLs with the expression of far-away genes. Nucleic Acids Res. *42*, 87–96. https://doi.org/10.1093/nar/gkt857.
5. Soler-Oliva, M.E., Guerrero-Martínez, J.A., Bachetti, V., and Reyes, J.C. (2017). Analysis of the relationship between coexpression domains and chromatin 3D organization. PLoS Comput. Biol. *13*, e1005708. https://doi.org/10.1371/journal.pcbi.1005708.
6. Brown, A.A., Fernandez-Tajes, J.J., Hong, M.G., Brorsson, C.A., Koivula, R.W., Davtian, D., Dupuis, T., Sartori, A., Michalettou, T.-D., Forgie, I.M., et al. (2023). Genetic analysis of blood molecular phenotypes reveals common properties in the regulatory networks affecting complex traits. Nat. Commun. *14*, 5062. https://doi.org/10.1038/s41467-023-40569-3.
7. Tambets, R., Kolde, A., Kolberg, P., Love, M.I., and Alasoo, K. (2024). Extensive co-regulation of neighboring genes complicates the use of eQTLs in target gene prioritization. HGG Adv. *5*, 100348. https://doi.org/10.1016/j.xhgg.2024.100348.
8. Aguet, F., Alasoo, K., Li, Y.I., Battle, A., Im, H.K., Montgomery, S.B., and Lappalainen, T. (2023). Molecular quantitative trait loci. Nat. Rev. Methods Primers *3*, 4. https://doi.org/10.1038/s43586-022-00188-6.
9. Lonsdale, J., Thomas, J., Salvatore, M., Phillips, R., Lo, E., Shad, S., Hasz, R., Walters, G., Garcia, F., Young, N., et al. (2013). The Genotype-Tissue Expression (GTEx) project. Nat. Genet. *45*, 580–585. https://doi.org/10.1038/ng.2653.
10. GTEx Consortium (2020). The GTEx Consortium atlas of genetic regulatory effects across human tissues. Science *369*, 1318–1330. https://doi.org/10.1126/science.aaz1776.
11. Saha, A., and Battle, A. (2018). False positives in trans-eQTL and co-expression analyses arising from RNA-sequencing alignment errors. F1000Res. *7*, 1860. https://doi.org/10.12688/f1000research.17145.2.
12. Mudge, J.M., Carbonell-Sala, S., Diekhans, M., Martinez, J.G., Hunt, T., Jungreis, I., Loveland, J.E., Arnan, C., Barnes, I., Bennett, R., et al. (2025). GENCODE 2025: reference gene annotation for human and mouse. Nucleic Acids Res. *53*, D966–D975. https://doi.org/10.1093/nar/gkae1078.
13. Nasser, J., Bergman, D.T., Fulco, C.P., Guckelberger, P., Doughty, B.R., Patwardhan, T.A., Jones, T.R., Nguyen, T.H., Ulirsch, J.C., Lekschas, F., et al. (2021). Genome-wide enhancer maps link risk variants to disease genes. Nature *593*, 238–243. https://doi.org/10.1038/s41586-021-03446-x.
14. Perez, G., Barber, G.P., Benet-Pages, A., Casper, J., Clawson, H., Diekhans, M., Fischer, C., Gonzalez, J.N., Hinrichs, A.S., Lee, C.M., et al. (2025). The UCSC Genome Browser database: 2025 update. Nucleic Acids Res. *53*, D1243–D1249. https://doi.org/10.1093/nar/gkae974.
15. Dyer, S.C., Austine-Orimoloye, O., Azov, A.G., Barba, M., Barnes, I., Barrera-Enriquez, V.P., Becker, A., Bennett, R., Beracochea, M., Berry, A., et al. (2025). Ensembl 2025. Nucleic Acids Res. *53*, D948–D957. https://doi.org/10.1093/nar/gkae1071.
16. Rozowsky, J., Gao, J., Borsari, B., Yang, Y.T., Galeev, T., Gürsoy, G., Epstein, C.B., Xiong, K., Xu, J., Li, T., et al. (2023). The EN-TEx resource of multi-tissue personal epigenomes &

variant-impact models. Cell *186*, 1493–1511.e40. https://doi.org/10.1016/j.cell.2023.02.018.

17. Liu, T., Porter, J., Zhao, C., Zhu, H., Wang, N., Sun, Z., Mo, Y.-Y., and Wang, Z. (2019). TADKB: Family classification and a knowledge base of topologically associating domains. BMC Genom. *20*, 217. https://doi.org/10.1186/s12864-019-5551-2.
18. Wang, G., Sarkar, A., Carbonetto, P., and Stephens, M. (2020). A Simple New Approach to Variable Selection in Regression, with Application to Genetic Fine Mapping. J. R. Stat. Soc. Series B Stat. Methodol. *82*, 1273–1300. https://doi.org/10.1111/rssb.12388.
19. Taylor-Weiner, A., Aguet, F., Haradhvala, N.J., Gosai, S., Anand, S., Kim, J., Ardlie, K., Van Allen, E.M., and Getz, G. (2019). Scaling computational genomics to millions of individuals with GPUs. Genome Biol. *20*, 228. https://doi.org/10.1186/s13059-019-1836-7.
20. Mohammadi, P., Castel, S.E., Brown, A.A., and Lappalainen, T. (2017). Quantifying the regulatory effect size of cis-acting genetic variation using allelic fold change. Genome Res. *27*, 1872–1884. https://doi.org/10.1101/gr.216747.116.
21. McLaren, W., Gil, L., Hunt, S.E., Riat, H.S., Ritchie, G.R.S., Thormann, A., Flicek, P., and Cunningham, F. (2016). The Ensembl Variant Effect Predictor. Genome Biol. *17*, 122. https://doi.org/10.1186/s13059-016-0974-4.
22. ENCODE Project Consortium, Moore, J.E., Purcaro, M.J., Pratt, H.E., Epstein, C.B., Shoresh, N., Adrian, J., Kawli, T., Davis, C.A., Dobin, A., et al. (2020). Expanded encyclopaedias of DNA elements in the human and mouse genomes. Nature *583*, 699–710. https://doi.org/10.1038/s41586-020-2493-4.
23. Zou, Y., Carbonetto, P., Wang, G., and Stephens, M. (2022). Fine-mapping from summary data with the "Sum of Single Effects" model. PLoS Genet. *18*, e1010299. https://doi.org/10.1371/journal.pgen.1010299.
24. Barbeira, A.N., Bonazzola, R., Gamazon, E.R., Liang, Y., Park, Y., Kim-Hellmuth, S., Wang, G., Jiang, Z., Zhou, D., Hormozdiari, F., et al. (2021). Exploiting the GTEx resources to decipher the mechanisms at GWAS loci. Genome Biol. *22*, 49. https://doi.org/10.1186/s13059-020-02252-4.
25. Purcell, S., Neale, B., Todd-Brown, K., Thomas, L., Ferreira, M.A.R., Bender, D., Maller, J., Sklar, P., de Bakker, P.I.W., Daly, M.J., and Sham, P.C. (2007). PLINK: A Tool Set for Whole-Genome Association and Population-Based Linkage Analyses. Am. J. Hum. Genet. *81*, 559–575. https://doi.org/10.1086/519795.
26. Leek, J.T., and Storey, J.D. (2007). Capturing Heterogeneity in Gene Expression Studies by Surrogate Variable Analysis. PLoS Genet. *3*, 1724–1735. https://doi.org/10.1371/journal.pgen.0030161.
27. Stegle, O., Parts, L., Piipari, M., Winn, J., and Durbin, R. (2012). Using probabilistic estimation of expression residuals (PEER) to obtain increased power and interpretability of gene expression analyses. Nat. Protoc. *7*, 500–507. https://doi.org/10.1038/nprot.2011.457.
28. Mostafavi, H., Spence, J.P., Naqvi, S., and Pritchard, J.K. (2023). Systematic differences in discovery of genetic effects on gene expression and complex traits. Nat. Genet. *55*, 1866–1875. https://doi.org/10.1038/s41588-023-01529-1.
29. Hubert, K.A., and Wellik, D.M. (2023). Hox genes in development and beyond. Dev. Camb. Engl. *150*, dev192476. https://doi.org/10.1242/dev.192476.
30. Bähler, L., Schärli, S., Luther, F., Bertschi, N.L., Skabytska, Y., Roediger, B., and Schlapbach, C. (2025). IL-18 in atopic dermatitis—a multifaceted driver of skin inflammation. J. Allergy Clin. Immunol. *0*. https://doi.org/10.1016/j.jaci.2025.07.025.
31. Wu, J., Zhang, X., Wu, D., Jin, O., and Gu, J. (2023). Evaluation of causal associations between interleukin-18 levels and immune-mediated inflammatory diseases: a Mendelian randomization study. BMC Med. Genomics *16*, 306. https://doi.org/10.1186/s12920-023-01744-z.
32. Hu, Y., Wang, J., Zhang, H., Xie, H., Song, W., Jiang, Q., Zhao, N., and He, S. (2017). Enhanced Expression of IL-18 and IL-18BP in Plasma of Patients with Eczema: Altered Expression of IL-18BP and IL-18 Receptor on Mast Cells. Mediators Inflamm. *2017*, 3090782. https://doi.org/10.1155/2017/3090782.
33. Abell, N.S., DeGorter, M.K., Gloudemans, M.J., Greenwald, E., Smith, K.S., He, Z., and Montgomery, S.B. (2022). Multiple causal variants underlie genetic associations in humans. Science *375*, 1247–1254. https://doi.org/10.1126/science.abj5117.
34. Okamoto, J., Yin, X., Ryan, B., Chiou, J., Luca, F., Pique-Regi, R., Im, H.K., Morrison, J., Burant, C., Fauman, E.B., et al. (2024). Probabilistic Fine-mapping of Putative Causal Genes. Preprint at bioRxiv. https://doi.org/10.1101/2024.10.27.620482.
35. Zou, Y., Carbonetto, P., Xie, D., Wang, G., and Stephens, M. (2026). Fast and flexible joint fine-mapping of multiple traits via the Sum of Single Effects model. Nat. Genet. *58*, 454–462. https://doi.org/10.1038/s41588-025-02486-7.
36. Eulalio, T., Sun, M.W., Gevaert, O., Greicius, M.D., Montine, T.J., Nachun, D., and Montgomery, S.B. (2025). regionalpcs improve discovery of DNA methylation associations with complex traits. Nat. Commun. *16*, 368. https://doi.org/10.1038/s41467-024-55698-6.
37. Waszak, S.M., Delaneau, O., Gschwind, A.R., Kilpinen, H., Raghav, S.K., Witwicki, R.M., Orioli, A., Wiederkehr, M., Panousis, N.I., Yurovsky, A., et al. (2015). Population Variation and Genetic Control of Modular Chromatin Architecture in Humans. Cell *162*, 1039–1050. https://doi.org/10.1016/j.cell.2015.08.001.
38. Li, S., Schmid, K.T., de Vries, D.H., Korshevniuk, M., Losert, C., Oelen, R., van Blokland, I.V., BIOS Consortium sc-eQTLgen Consortium, Groot, H.E., Swertz, M.A., et al. (2023). Identification of genetic variants that impact gene co-expression relationships using large-scale single-cell data. Genome Biol. *24*, 80. https://doi.org/10.1186/s13059-023-02897-x.
39. Kolberg, L., Kerimov, N., Peterson, H., and Alasoo, K. (2020). Co-expression analysis reveals interpretable gene modules controlled by trans-acting genetic variants. eLife *9*, e58705. https://doi.org/10.7554/eLife.58705.
40. Wang, L., Babushkin, N., Liu, Z., and Liu, X. (2024). Trans-eQTL mapping in gene sets identifies network effects of genetic variants. Cell Genom. *4*, 100538. https://doi.org/10.1016/j.xgen.2024.100538.
41. Zhou, X., and Cai, X. (2021). Joint eQTL mapping and inference of gene regulatory network improves power of detecting both cis- and trans-eQTLs. Bioinformatics *38*, 149–156. https://doi.org/10.1093/bioinformatics/btab609.

The American Journal of Human Genetics, Volume *113*

Supplemental information

Focus on single-gene effects limits discovery and interpretation of complex-trait-associated variants

Kathryn A. Lawrence, Tamara Gjorgjieva, Daniel Nachun, and Stephen B. Montgomery

Supplemental Figures

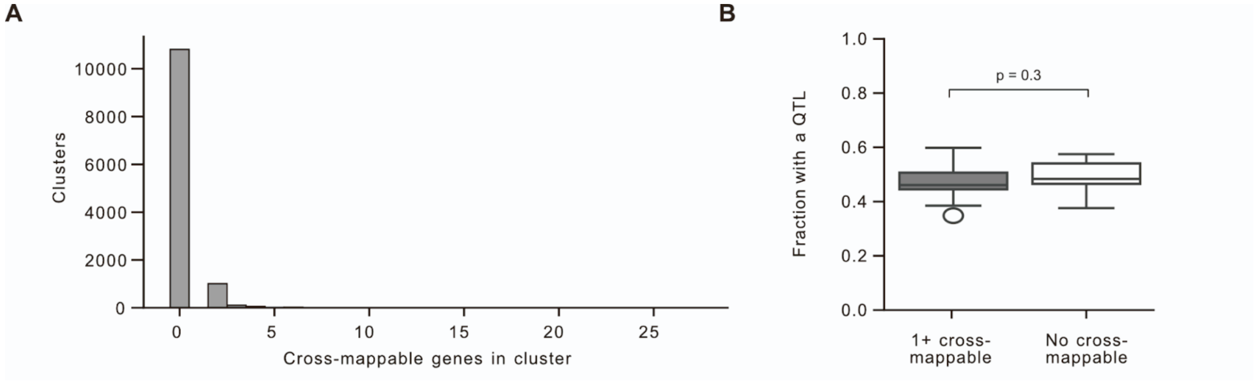

Figure S1: Cross-mappability of clusters.
(A) Histogram of the number of genes in each cluster with more than 100 cross-mappable 75-mers.
(B) Fraction of gene clusters with at least one significant QTL for clusters with at least one cross-mappable gene pair or with no cross mappable gene pairs; the *p* value is from a two-sample *t* test.

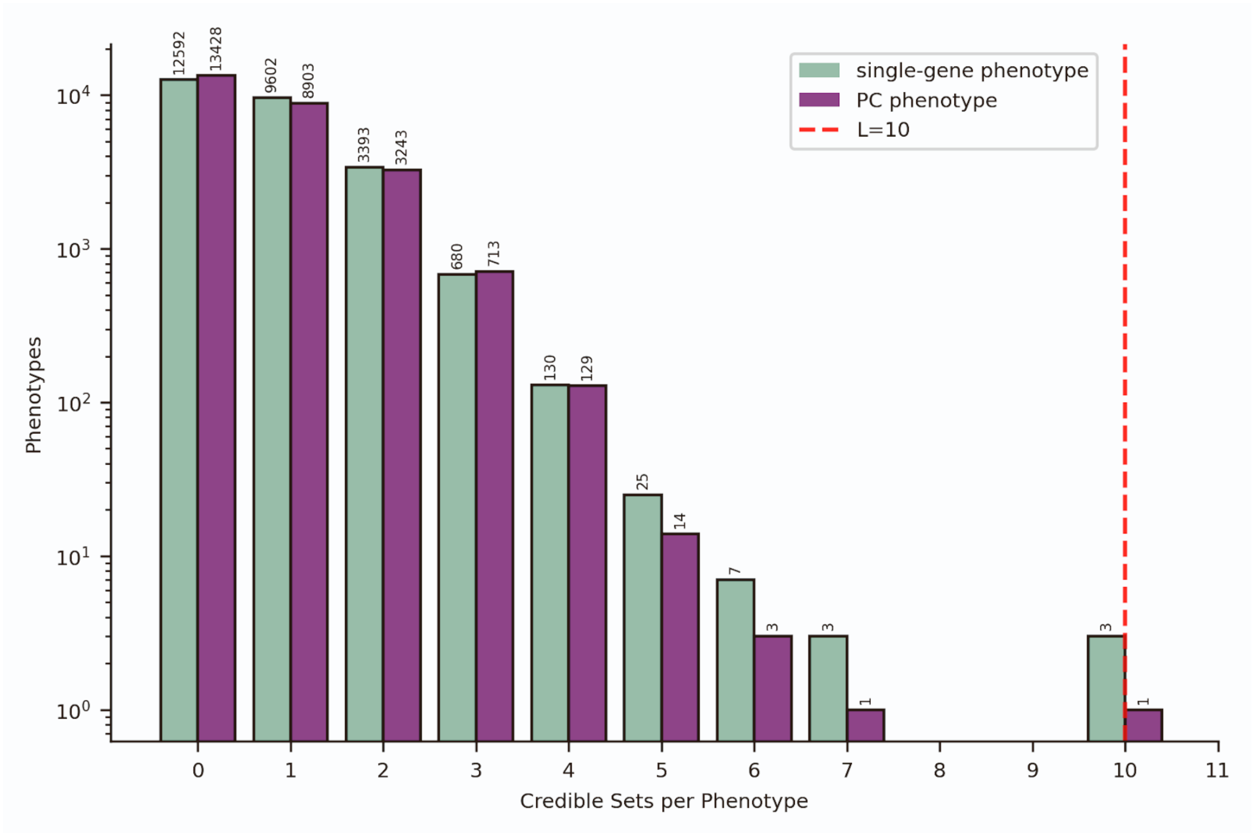

Figure S2: Credible sets per phenotype.
Number of credible sets mapped per PC phenotype (pcQTL) and expression phenotype (eQTL). The dotted red line shows 10 credible sets, the maximum number of credible sets possible for a single phenotype with SuSiE run with L = 10.

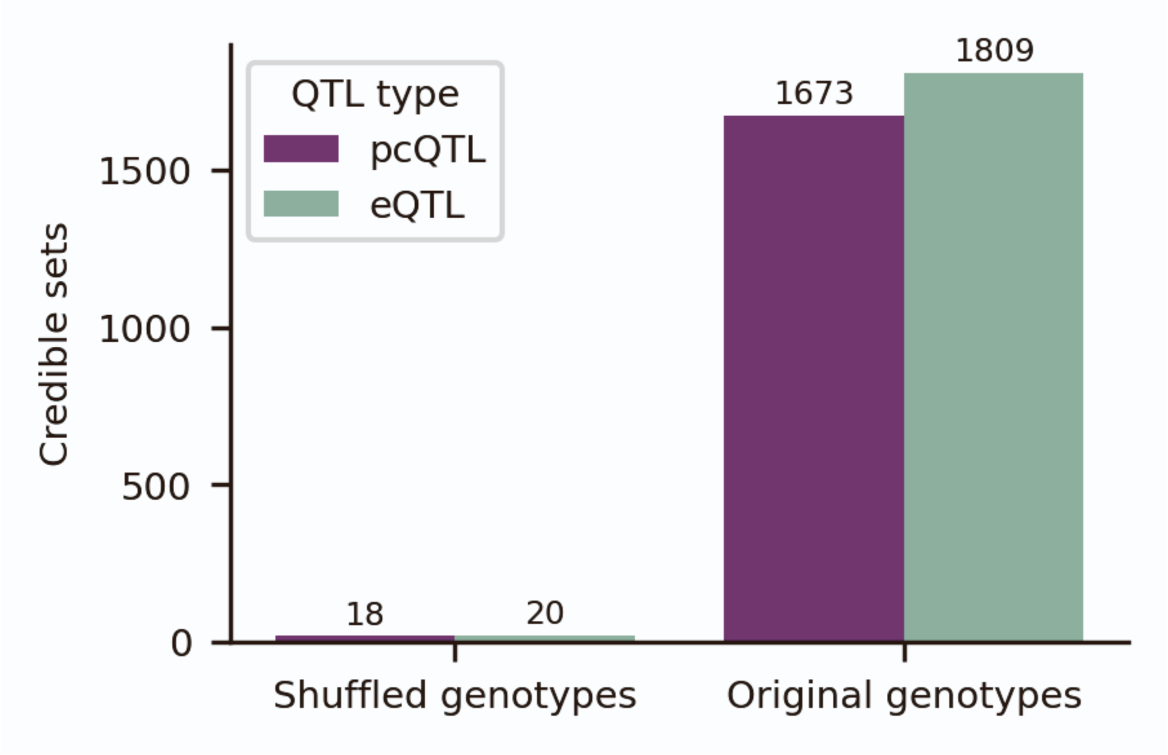

Figure S3: pcQTL mapping null with shuffled genotypes.
For each single-gene expression and PC phenotype for clusters in fibroblast tissue, the genotypes at each variant position were shuffled. SuSiE was then run with the shuffled genotype and with the real genotypes to map pcQTLs and eQTLs. PC phenotypes were not significantly more likely to map false positive pcQTLs with the shuffled genotype than single-gene expression phenotypes were to map false positive eQTLs with the shuffled genotype and eGenes; Fisher's exact p = 1.0.

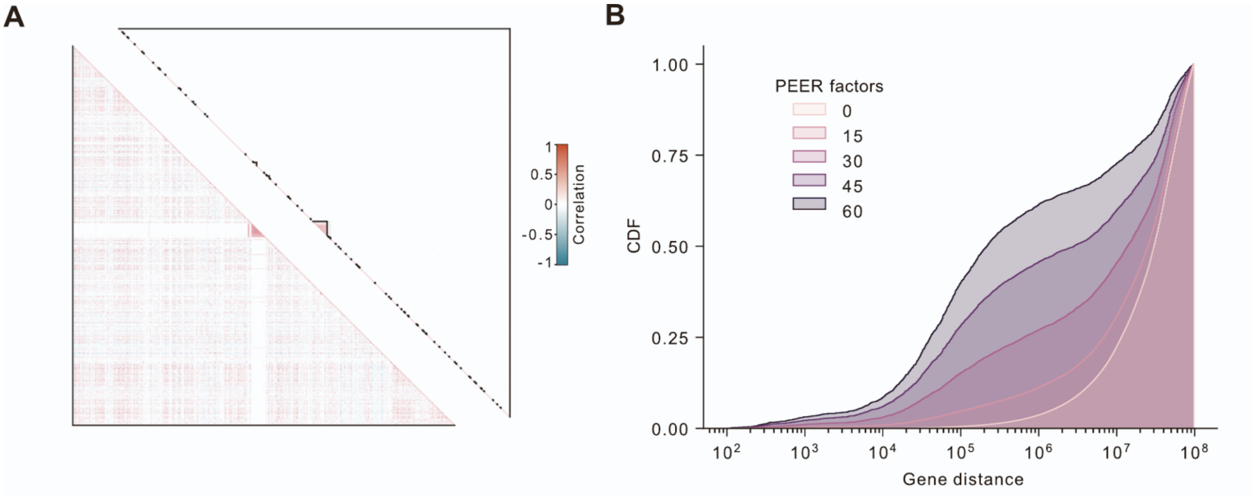

Figure S4: PEER factor residualization's effect on correlation.

(A) Correlation matrix of gene expression in chromosome 17 for sun exposed skin; Spearman's correlation before (lower) and after (upper) residualization with 60 PEER factors. Correlations with Bernoulli-corrected $p < 0.05$ are shown. Clusters are outlined in black on the upper matrix.

(B) CDF of pairwise gene distances of genes with significantly correlated gene expression profiles after residualization with 0, 15, 30, 45, and 60 PEER factors.

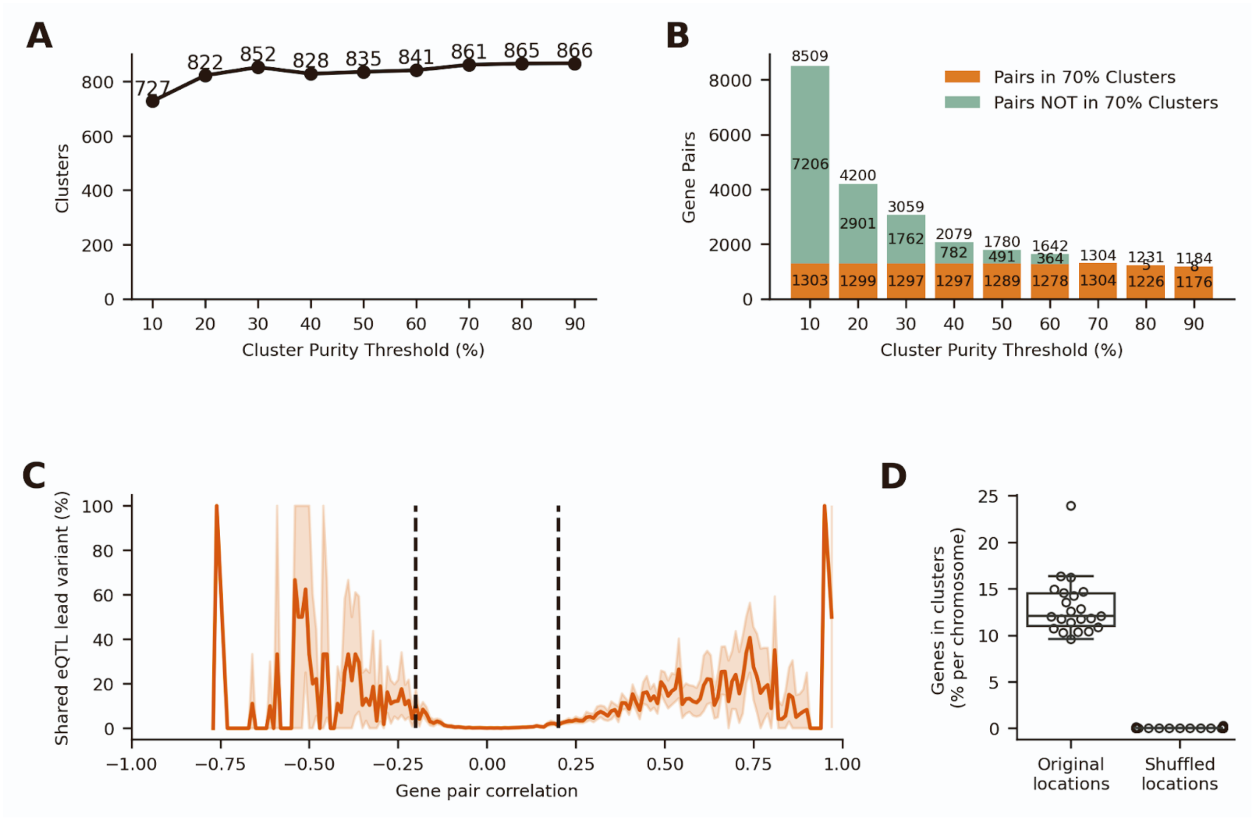

Figure S5: Clustering sensitivity to hyperparameters.

(A) Number of clusters in fibroblasts vs clustering purity thresholds from 10–90%.

(B) Gene pairs in fibroblasts clusters vs clustering purity thresholds from 10–90%, colored by whether the gene-pair was included in clusters at a 70% purity threshold used in this analysis.

(C) Fraction of gene-pairs with a shared single-gene eQTL lead variant vs gene-pair correlation for all neighboring gene pairs. The dotted lines represent the mean correlation cutoffs used for our clusters.

(D) Fraction genes in clusters called on expression data for fibroblasts with relative gene locations shuffled. Each point is a chromosome.

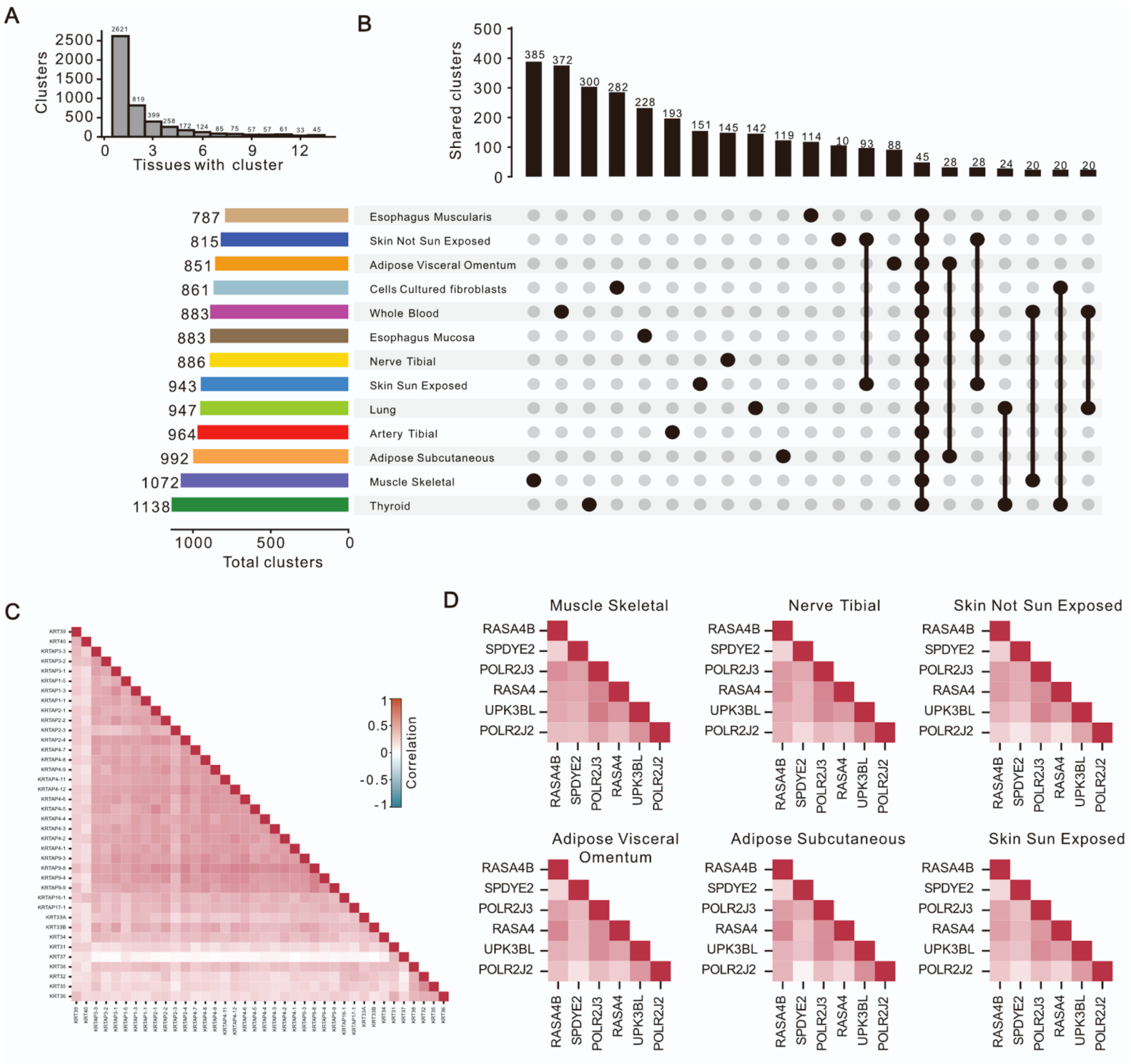

Figure S6: Tissue specificity of clusters.

(A) Histogram of the number of tissues a cluster is in.

(B) Upset plot detail of which tissues a cluster is shared across for all tissue combinations with 20 or more clusters.

(C) A tissue-specific thirty-eight gene cluster on chromosome 17 from sun-exposed skin gene expression. Color is Spearman's correlation of expression.

(D) A six gene cluster on chromosome 6, shared across 6 tissues. Color is Spearman's correlation of expression.

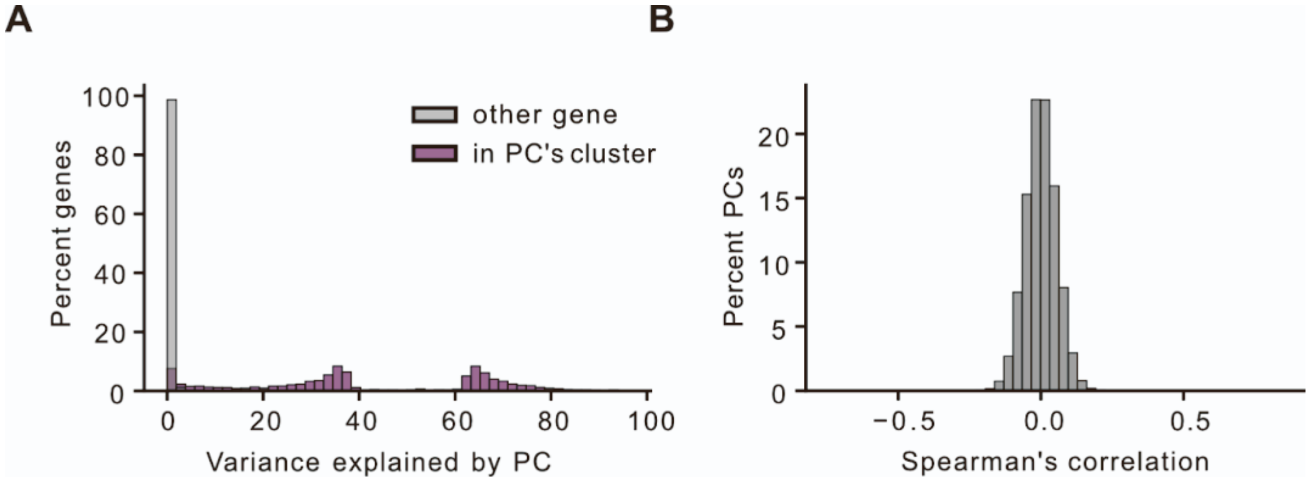

Figure S7: Local PC properties.
(A) Variance explained by each PC phenotype for all genes split by whether or not the gene is in the cluster whose expression was used to calculate the PC.
(B) Distribution of Spearman's correlation from each PC to all other PCs for all fibroblast PCs.

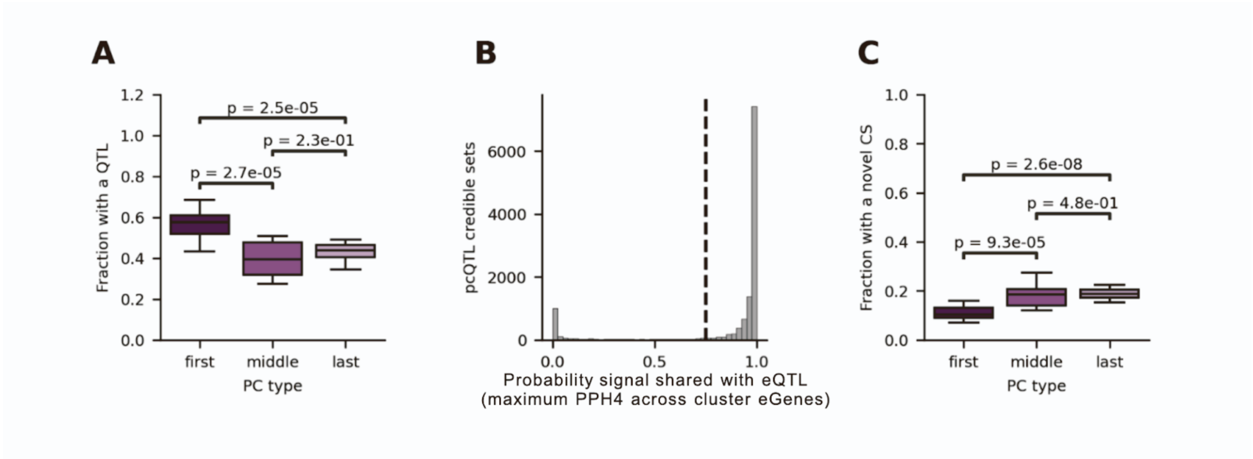

Figure S8: pcQTL discoveries by PC order.

(A) The fraction of PC phenotypes across tissues with at least one significant QTL split into the primary (first) PC, the last PC, and all other PCs (middle); p values are from a two-sample t test.

(B) Distribution of the maximum PP_{H4} colocalization probability for each pcQTL credible set with any cluster eQTL credible set. Dotted line PP_{H4} = 0.75.

(C) The fraction of PC phenotypes across tissues with at least one novel significant QTL split into the primary (first) PC, the last PC, and all other PCs (middle); p values are from a two sample t test.

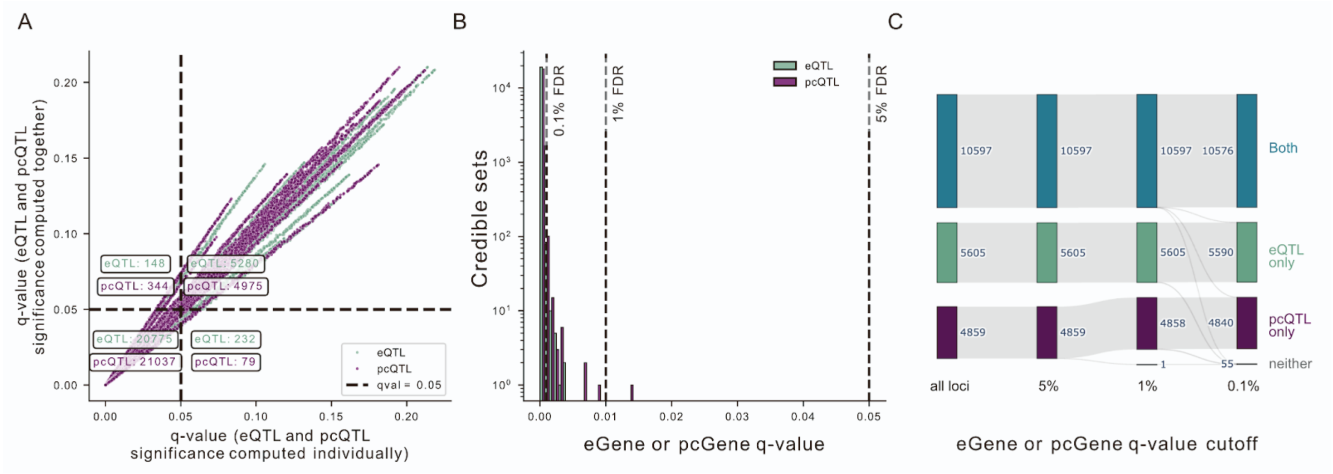

Figure S9: Phenotype filtering by FDR prior to fine-mapping.
(A) *q* values from permutation mode of tensorQTL for single-gene expression phenotypes and PC phenotypes each run together, vs each run individually. Boxes give the number of single-gene expression and PC phenotypes in each quadrant, with significance cutoffs of $q < 0.05$, a 5% FDR.
(B) For each single-gene expression or PC phenotype with a significant fine-mapped SuSiE credible set, the distribution of tensorQTL permutation *q* values from combined mapping on single-gene expression and PC phenotypes together. Dotted lines represent *q* values for 5%, 1% and 0.1% FDR cutoffs. All single-gene expression and PC phenotypes with a credible set would pass 5% FDR. All single-gene expression phenotypes and all but one PC phenotype with a credible set would pass 1% FDR.
(C) Discovery of credible-set-groups at various combined *q* values thresholds in a two-step QTL mapping process. At each *q* values cutoff, we subset to only credible sets mapped on single-gene expression and PC phenotypes with $q <$ cutoff. Then, pcQTL and eQTL credible sets are again grouped by colocalizations and the groups are classified according to whether the contain only pcQTL credible sets, only eQTL credible sets, both eQTL and pcQTL credible sets, or no longer contain any credible sets.

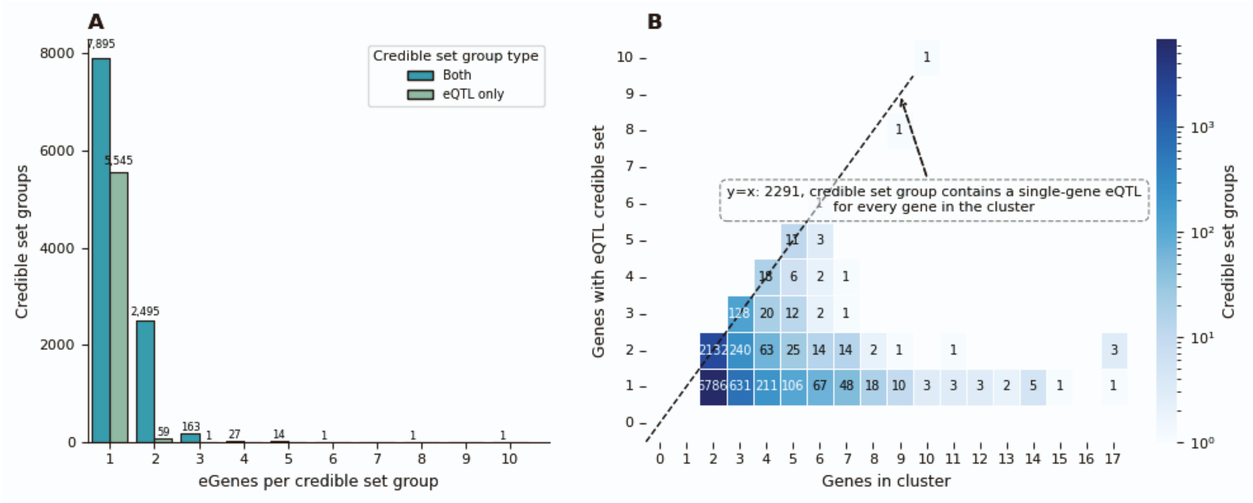

Figure S10: eGene colocalization.

(A) Number of eGenes colocalized in each credible set group split by whether the group is eQTL only or both a pcQTL and eQTL.

(B) Distribution of the number of genes in a cluster vs the number of genes that are single-gene eQTLs for credible set groups that contain both eQTLs and pcQTLs. 2,291 credible set groups contain a single-gene eQTL for every gene in the cluster.

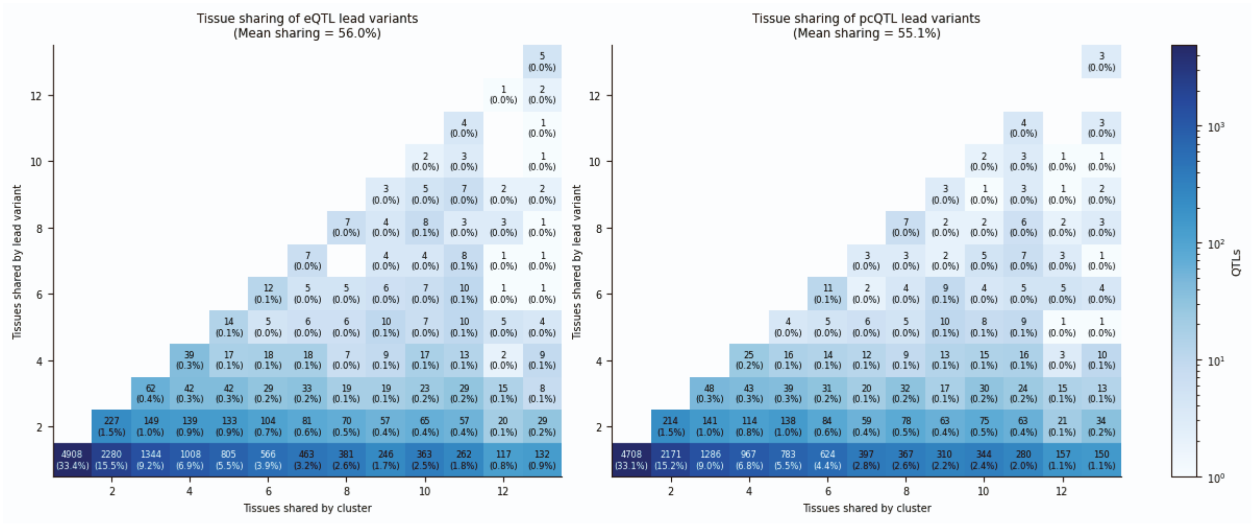

Figure S11: Lead variant sharing.

For eQTL lead variants (left) and pcQTL lead variants (right), the number of tissues that share the lead variant vs the number of tissues in which the QTL's cluster was mapped. For each lead variant, percent sharing is computed as the number of tissues with the shared lead variant divided by the number of tissues with the shared cluster multiplied by 100. The mean sharing is the mean percent sharing across lead variants. pcQTLs are slightly, but statistically significantly, more tissue specific, with lower sharing; Mann-Whitney *U* test $p = 0.011$.

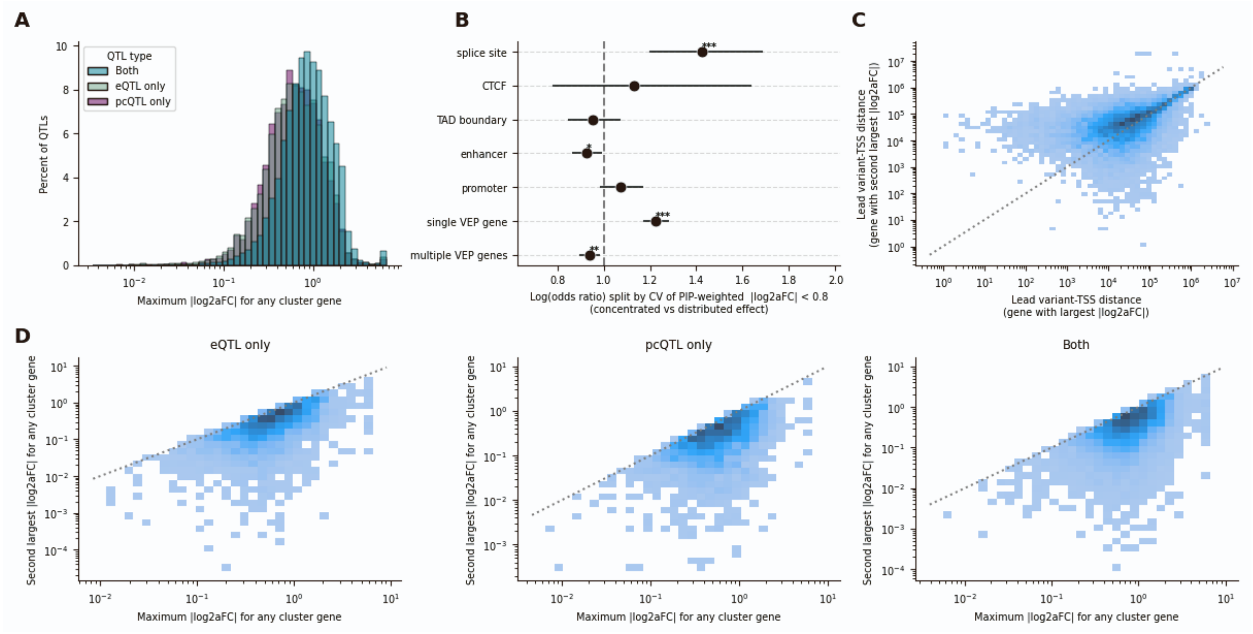

Figure S12: Distribution of single-gene $\log_2$aFC effects.

(A) Maximum PIP-weighted $|\log_2\text{aFC}|$ for any gene in a cluster, split by if the QTL is discovered as both a pcQTL and eQTL, an eQTL only, or a pcQTL only. Maximum $|\log_2\text{aFC}|$ for credible sets discovered as pcQTLs only or eQTLs only were not significantly different ($p = 0.333$) from each other. Maximum $|\log_2\text{aFC}|$ for credible sets discovered as both pcQTLs and eQTLs were significantly higher than those discovered as pcQTLs only ($p < 10^{-10}$) or eQTLs only ($p < 10^{-10}$); p values are from Mann-Whitney U test.

(B) Annotation enrichments for PIP-weighted variant effect predictor categories for QTL credible sets split by if the CV of PIP-weighted $|\log_2\text{aFC}| < 0.8$. Error bars are 95% confidence intervals on odds ratios for logistic regression (*$0.05 > p > 10^{-2}$, **$10^{-2} > p > 10^{-3}$, and ***$p < 10^{-3}$).

(C) The distance from the lead variant of the QTL credible set to the TSS of the gene with the second largest $|\log_2\text{aFC}|$ vs the distance from the lead variant to the TSS of the gene with the largest $|\log_2\text{aFC}|$ gene.

(D) The second largest PIP-weighted $|\log_2\text{aFC}|$ for each QTL on any eGene in a cluster vs the largest PIP-weighted $|\log_2\text{aFC}|$ on any eGene in a cluster, split by if the QTL is discovered as both a pcQTL and eQTL, an eQTL only, or a pcQTL only.

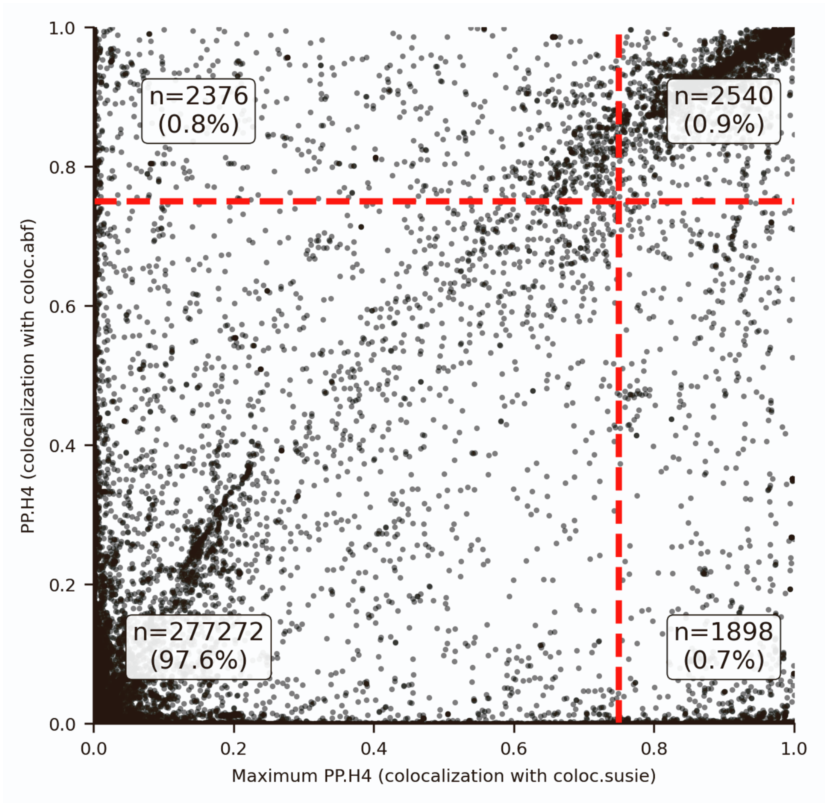

Figure S13: Single-causal variant colocalization.
For each single-gene expression phenotype or PC phenotype and GWAS trait, posterior probability of colocalization (PP_{H4}) between GWAS and QTL phenotypes assuming a single-causal variant (coloc.abf) vs maximum PP_{H4} over all credible sets for colocalization with run SuSiE, allowing for multiple causal variants (coloc.susie). Dotted line PP_{H4} = 0.75. Boxes show the number and percent of GWAS-phenotype pairs in each quadrant.

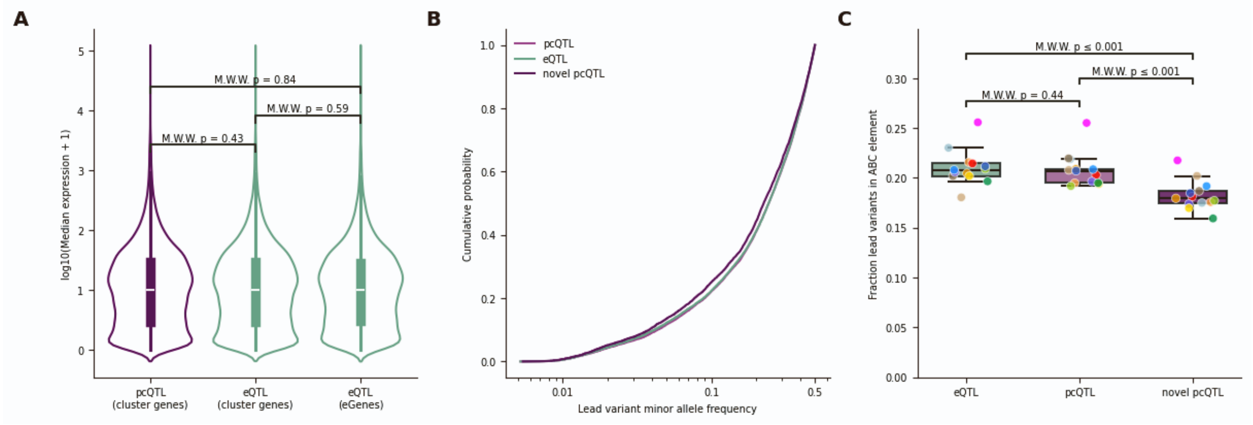

Figure S14: Properties of pcQTLs and eQTLs.

(A) In each tissue, the distribution of median expression of all genes in a cluster that had a significant pcQTL, the distribution of median expression of all genes in a cluster that had a significant eQTL, and the distribution of median expression of eGenes from clusters that had a significant eQTL; *p* values are from Mann-Whitney *U* test.

(B) Minor allele frequencies of lead variants for QTL credible sets discovered as pcQTLs or eQTLs are not significantly different ($p = 0.164$). Minor allele frequencies for novel pcQTL lead variants are lower than eQTL lead variants ($p = 1.9*10^{-4}$), and all pcQTL lead variants ($p = 1.3*10^{-6}$); *p* values are from Mann-Whitney *U* test.

(C) For each lead variant for a QTL credible set, the fraction that falls within an ABC “genic” or “intergenic” regulatory element. eQTL is all eQTL credible set lead variants, pcQTL is all pcQTL credible set lead variants, novel pcQTL is lead variants from pcQTL credible sets that did not colocalize with any eQTL credible sets; *p* values are from Mann-Whitney *U* test.

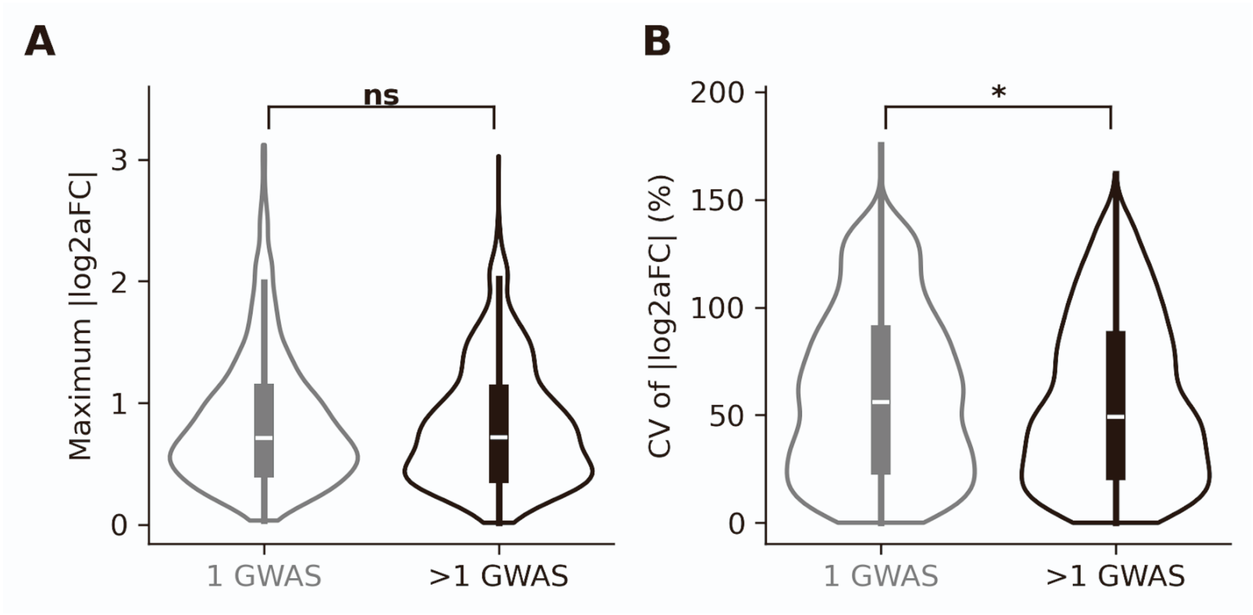

Figure S15: GWAS pleiotropy.
(A) For all credible set groups that colocalize with at least one GWAS hit, the maximum PIP-weighted $|\log_2 aFC|$ for any single gene in the cluster, split by whether or not the credible set group colocalizes with more than one GWAS; two-sample *t* test *p* = 0.38.
(B) For all credible set groups that colocalize with at least one GWAS hit, the CV of PIP-weighted $|\log_2 aFC|$ across genes in the cluster (quantifying how concentrated vs distributed the effect is) split by whether or not the credible set group colocalizes with multiple GWAS hits; two-sample *t* test *p* = 0.02.

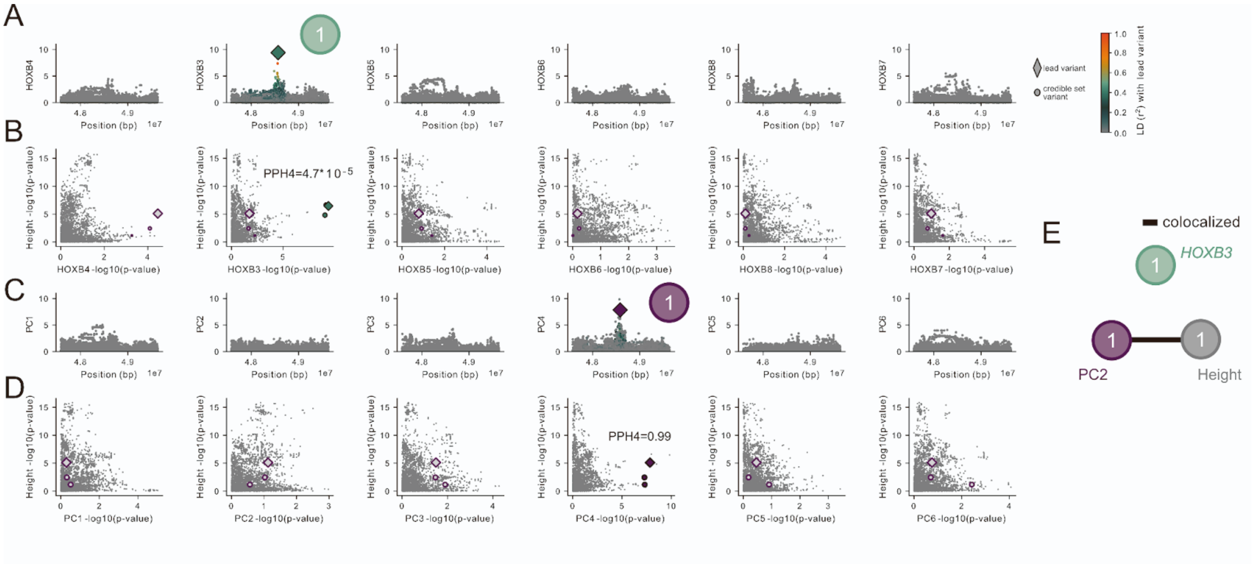

Figure S16: Height colocalization for a *HOXB* cluster eQTL and pcQTL.

(A) QTL nominal *p* values for each gene's expression vs genomic position. If the phenotype has any significant credible sets, credible set variants are highlighted and colors given by variant LD with the credible-set lead variant.

(B) GWAS variant nominal *p* values vs QTL nominal *p* values for each gene's expression. If the phenotype has any significant credible sets, credible set variants are highlighted filled diamonds/circles and the highest PP_{H4} (probability of colocalization) is given between QTL credible set and any GWAS credible sets. The novel pcQTL credible set is highlighted with outlined diamonds/circles.

(C) Same as (A), but for each PC phenotype.

(D) Same as (B), but for each PC phenotype.

(E) Colocalizations between credible sets in the cluster, colocalized is $PP_{H4} > 0.75$.

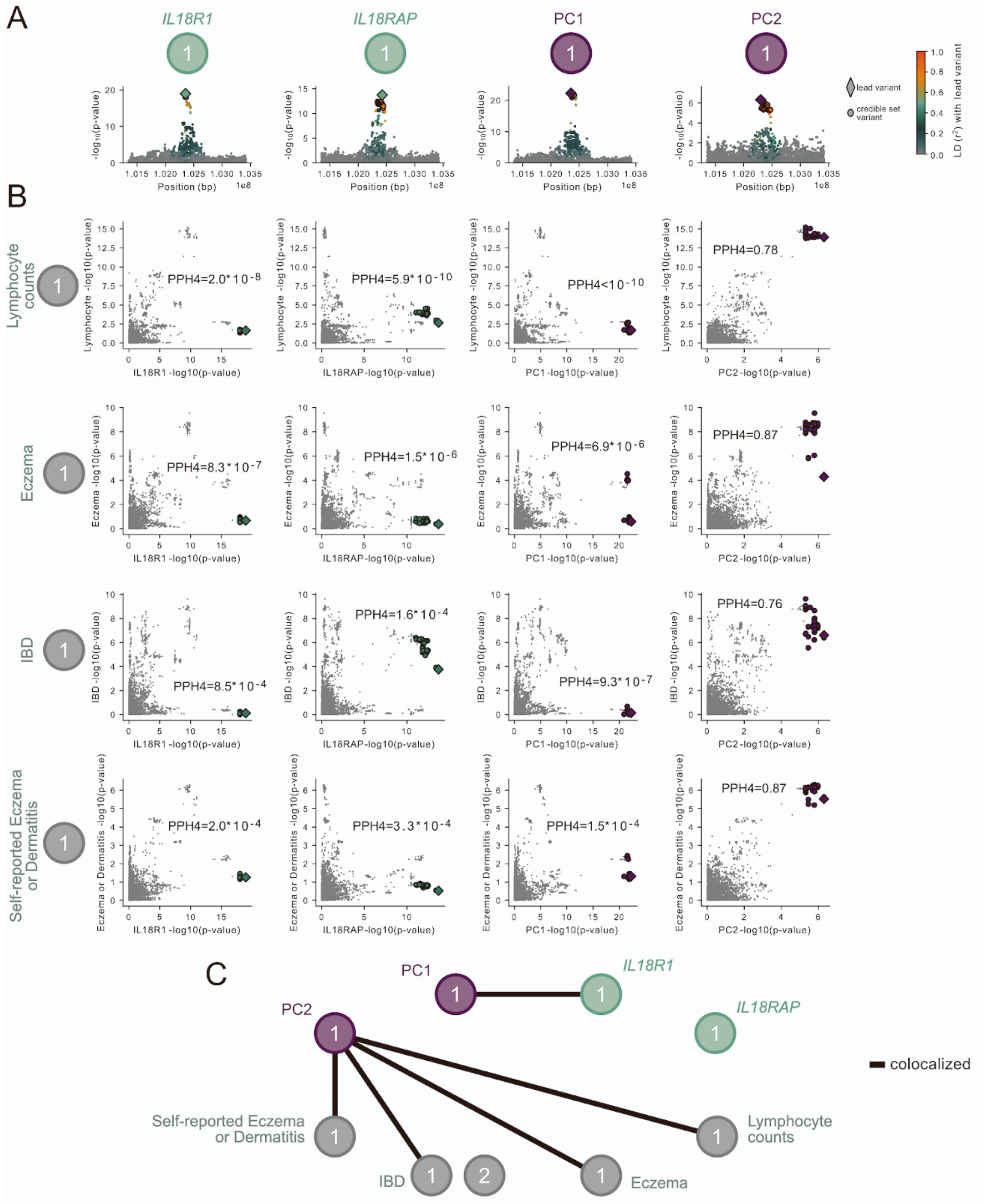

Figure S17: Lymphocyte count, eczema, IBD, and dermatitis colocalization for *IL18R1* and *IL19RAP* eQTL and pcQTL.

(A) QTL nominal *p* values for each gene's expression and each PC vs genomic position. If the phenotype has any significant credible sets, a plot is shown for each credible set, with credible set variants highlighted and colors given by variant LD with the credible-set lead variant.

(B) GWAS variant nominal p-values vs QTL nominal *p* values for each gene's expression and each PC, with credible set variants highlighted and the highest probability of colocalization (PP_{H4}) between any QTL phenotype credible set and any GWAS credible set.

(C) Colocalizations between credible sets in the cluster, colocalized is $PP_{H4} > 0.75$.

Table S1: GWAS colocalizations across tissues and traits. The total number of GWAS hits near clusters is the number of unique GWAS lead variants fine-mapped within 1MB of a gene cluster. The total number of GWAS hits near clusters per tissue is the number of unique GWAS lead variants fine-mapped within 1MB of a gene cluster in each tissue. The number of GWAS hits colocalized per GWAS trait in each tissue is given for eQTLs and for novel pcQTLs (pcQTLs not colocalized by an eQTL).
